# Italian hospitals on the web: a cross-sectional analysis of official websites

**DOI:** 10.1186/1472-6947-10-17

**Published:** 2010-04-01

**Authors:** Giovanni Maifredi, Grazia Orizio, Maura Bressanelli, Serena Domenighini, Cinzia Gasparotti, Eleonora Perini, Luigi Caimi, Peter J Schulz, Umberto Gelatti

**Affiliations:** 1 Section of Hygiene, Epidemiology and Public Health, Department of Experimental and Applied Medicine, University of Brescia, Brescia, Italy; 2 "Quality and Technology Assessment, Governance and Communication Strategies in Health Systems" Study and Research Centre, University of Brescia, Brescia, Italy; 3 Institute of Communication and Care, University of Lugano, Lugano, Switzerland

## Abstract

**Background:**

Although the use of the Internet for health purposes has increased steadily in the last decade, only a few studies have explored the information provided by the websites of health institutions and no studies on the on-line activities of Italian hospitals have been performed to date. The aim of this study was to explore the characteristics of the contents and the user-orientation of Italian hospital websites.

**Methods:**

The cross-sectional analysis considered all the Italian hospitals with a working website between December 2008 and February 2009. The websites were coded using an *ad hoc *Codebook, comprising eighty-nine items divided into five sections: technical characteristics, hospital information and facilities, medical services, interactive on-line services and external activities. We calculated a website evaluation score, on the basis of the items satisfied, to compare private (PrHs) and public hospitals, the latter divided into ones with their own website (PubHs-1) and ones with a section on the website of their Local Health Authority (PubHs-2). Lastly, a descriptive analysis of each item was carried out.

**Results:**

Out of the 1265 hospitals in Italy, we found that 419 of the 652 public hospitals (64.3%) and 344 of the 613 PrHs (56.1%) had a working website (p = 0.01). The mean website evaluation score was 41.9 for PubHs-1, 21.2 for PubHs-2 and 30.8 for PrHs (p < 0.001).

Only 5 hospitals out of 763 (< 1%) provided specific clinical performance indicators, such as the nosocomial infection rate or the surgical mortality rates. Regarding interactive on-line services, although nearly 80% of both public and private hospitals enabled users to communicate on-line, less than 18% allowed the reservation of medical services, and only 8 websites (1%) provided a health-care forum.

**Conclusions:**

A high percentage of hospitals did not provide an official website and the majority of the websites found had several limitations. Very few hospitals provided information to increase the credibility of the hospital and user confidence in the institution. This study suggests that Italian hospital websites are more a source of information on admissions and services than a means of communication between user and hospital.

## Background

Up-to-date statistics on Internet usage have revealed that Internet penetration rates among the population has reached 74.4% in North America and 48.8% in Europe, with impressive growth in recent years [1]. Besides, the use of the Internet for health purposes has steadily increased and it is now an important source of health information for patients and their families [2]. It has been estimated that, worldwide, about 4.5% of searches on the web are health related [3]. A survey conducted in the US clearly shows that using the Internet as a health information resource remains the prevalent health-related activity of Internet users, much more frequent than the purchase of medicine or the participation in online support groups [4]. Studies in the US have found that 56% to 80% of Internet users have looked for health information online, including details of doctors and hospitals [5,6], whereas the percentage of Internet health users in Europe ranges from 32% to 71%, with vast differences between countries and in terms of user age, gender and standard of education [7].

Italian statistics show that 42% of women and 34% of men have looked for health information on the Internet [8], a percentage very similar to those observed in other south European countries, such as Greece and Portugal [7]. In Italy, too, women use the Internet for health purposes more frequently than men [8], as has already been observed in other studies, especially among young people [4,7,9].

The potential public health impact of Internet use for health purposes has already been considered as both a public health threat and an advantage for users. Patients have direct access to information on diseases and treatments and they can even purchase online healthcare services, such as drugs, genetic tests and medical devices, thus bypassing the health professional, leading to possible risks [10-12]. Conversely, the potential of innovative communication tools for health care organizations in terms of public relations has already been reported and it represents an outstanding means of communication between users and institutions [13].

In addition, although the Internet is currently used more as a supplement to ordinary health services than a replacement of them, doctors should be aware that patients will increasingly ask for e-health services in the near future [14].

On their websites, health institutions such as hospitals can provide a large amount of information on the services they offer and how to access them, drawing the users' attention to themselves [15]. Patients can act as customers and decide to turn to the more attractive hospitals, hence a well-structured website can be the most effective way to gain patient confidence [16].

Recent advances in web technologies and user interfaces have greatly changed the web applications and in many cases transformed the way users interact with them [17]. The new generation of Internet devices and services can be very useful in facilitating participation and information sharing across a vast number of users [18], thus supporting personal involvement in hospital activities.

The aim of this study was to explore the information provided by the official websites of Italian hospitals and the possibility of interaction and communication between users and institutions via the Internet. To this end a cross-sectional study of all Italian hospital websites was conducted, and their technical characteristics and contents were analysed.

## Methods

### Study base

A list of all Italian hospitals was obtained from the Italian Ministry of Health (IMH) website updated to 1st January 2008 (see additional file 1). The hospitals were identified by an unambiguous code; records presenting the same identified code were considered only once, as they represent different administrative centres for the same hospital. We therefore included 1265 hospitals, meaning every Italian hospital, from the original 1570 records present in the IMH file.

### Website identification and selection

The study was carried out in two stages. First, in November 2008, we searched for official hospital websites using the Google search engine, entering the hospital name and town as keywords. The first 30 references were analysed and websites not found using Google were actively searched for using a common services search engine (http://www.paginegialle.it/, Italian yellow pages), entering the name, town and address of the hospitals.

The Italian National Health Service (INHS) was established in 1978 on the British NHS model and subsequently underwent a major reform in the 1990s that introduced a quasi-market system, regionalization and managerialism [19]. Regionalism implies that jurisdiction over health-care issues is devolved at a regional level and for this reason we decided to present the results in Table 1 stratified by region. The quasi-market system introduced competition between public and accredited private providers [19]. Italian hospitals are therefore divided by the IMH into public and private ones. The majority of public hospitals are under the direct management of their Local Health Authority (LHA), and the websites of these hospitals are, in most cases, pages on the LHA website rather than a separate website. For this category of hospital we searched directly in the corresponding LHA website if the first two methods of website identification failed.

**Table 1:** Number and percentage of hospitals with a working website (WW)

Region	Public hospitals		Private hospitals		P value	Total	
	
	WWs/Tot	% WWs	WWs/Tot	% WWs		WWs/Tot	% WWs
Abruzzo	9/22	40.9	8/13	61.5	0.24	17/35	48.6
Basilicata	5/10	50.0	0/1	0.0	0.34	5/11	45.5
Calabria	5/37	13.5	13/39	33.3	0.04	18/76	23.7
Campania	32/55	58.2	33/71	46.5	0.19	65/126	51.6
Emilia-Romagna	25/27	92.6	33/48	68.8	0.02	58/75	77.3
Friuli Venezia Giulia	13/16	81.3	4/5	80.0	0.95	17/21	81.0
Lazio	50/78	64.1	56/99	56.6	0.31	106/177	59.9
Liguria	12/18	66.7	5/10	50.0	0.39	17/28	60.7
Lombardy	58/61	95.1	64/82	78.0	0.01	122/143	85.3
Marches	10/33	30.3	7/13	53.8	0.14	17/46	37.0
Molise	2/7	28.6	1/3	33.3	0.88	3/10	30.0
Piedmont	28/40	70.0	25/51	49.0	0.04	53/91	58.2
Puglia	20/38	52.6	14/36	38.9	0.24	34/74	45.9
Sardinia	29/32	90.6	5/12	41.7	0.01	34/44	77.3
Sicily	49/70	70.0	32/65	49.2	0.01	81/135	60.0
Tuscany	22/42	52.4	18/31	58.1	0.63	40/73	54.8
Trentino-Alto Adige	15/16	93.8	8/11	72.7	0.13	23/27	85.2
Umbria	8/11	72.7	4/5	80.0	0.76	12/16	75.0
Aosta Valley	0/1	0.0	0/0	0.0	-	0/1	0.0
Veneto	27/38	71.1	14/18	77.8	0.60	41/56	73.2
Italy	419/652	64.3	344/613	56.1	0.01	763/1265	60.3

In order not to rule out this category of public hospital, these pages were included in the study, even if they did not have a proper website. However, we only included hospitals whose pages were clearly identified as single sections of the LHA websites and only information from these pages was taken into account.

Hospitals were classified into two groups according to their website: hospitals with an available and working website, defined as a working website (WW), and hospitals with a website that was not in operation or not available.

We therefore identified three categories of hospital with a WW: 1) Public hospitals with their own website, defined as public hospitals type 1 (PubHs-1); 2) Public hospitals with a section on the website of their LHA, defined as public hospitals type 2 (PubHs-2); 3) Private hospitals (PrHs), all of them with their own websites. We proceeded with an analysis of the websites of these three categories of hospitals by coding them.

### Coding of working websites

The second stage of the study concerned WW coding. Between December 2008 and February 2009, five raters (MG, GC, PE, BM, DS) independently coded an equal number of websites, randomly assigned on the basis of the geographical region and category of the hospital, using an *ad hoc *Codebook (additional file 2) drawn up according to the Content Analysis Method [20].

The Codebook was first tested in a preliminary study that included a random sample of 40 hospitals. Subsequently, concordance among raters was analysed for 20 randomly chosen websites and a good agreement was observed (Cohen's Kappa statistic calculated for each website, among all raters, ranging from 0.69 to 0.88, with a median value of 0.80).

In designing the Codebook, significant elements that emerged from previous studies [15,21,22] and attributes concerning website user-orientation were both taken into account, as described in detail at the end of this sub-section. We focused more on the characteristics of the website contents than on informatics quality aspects.

The Codebook consisted of 89 items divided into five sections, focusing on different contents.

1) Technical contents: 19 items, including the presence of a site map and internal search engine, and the certification of accessibility to people with disabilities provided by the Web Accessibility Initiative (WAI) [23]. We recorded whether the websites provided W3C-css [24] and W3C-html [25] certifications to assess whether the websites were in line with these technical informatics standards. We therefore looked for the presence of the Health On the Net (HON) foundation's logo as evidence of the reliability and credibility of the medical information provided by the site [26].

2) Hospital information and facilities: 22 items concerning general information, such as the history of the hospital, its location and ways of reaching the hospital, and contact details of the public relations office.

3) Medical services: since a hospital website is a major source of information on admissions and services, we examined 25 items concerning hospital admission, discharge and everyday life during the hospitalization period and information about the doctors employed at the hospitals.

4) Interactive on-line services: we investigated hospital use of the Internet using 10 items, such as the availability of on-line reservations, being able to communicate with the hospital via the Internet or e-mail, and the presence of a health-related forum.

5) External activities: this section of the Codebook concerns 13 items, such as being able to obtain health information, job opportunities and a list of conferences organized by the hospital.

The structure of the Codebook was based on the study by Mira and coll [15], although some items were not taken into account and many were introduced as new ones. Some items concerning the transparency of the website were taken from the "Quality Principles for Cultural Websites: a handbook" by the Minerva Working Group 5 [22] and we introduced some quality items that emerged from a systematic review of empirical studies assessing the quality of health information on the Web [21]. We also introduced some new items in the "Admission and medical services" section, i.e. hospital quality indicators and items regarding the waiting list, because we were interested in studying the degree of user-orientation of the hospital websites and in the "Interactive on-line services" section, such as the possibility of signing up for a newsletter and the presence of a health-related forum, in order to study the degree of interaction between hospitals and users.

### Website evaluation score

The Codebook was used to score each website, by identifying the presence or absence of the 89 items (1 = item found, 0 = item not found). We could therefore calculate a score for each website, defined as the website evaluation score, expressed as the percentage of items found over the total number of items, in order to compare the amount of information provided by each category of hospital, PubH-1, PubH-2 and PrH.

We first calculated a mean website evaluation score for each category of hospital, and subsequently we calculated a mean website evaluation score for each of the five sections of the Codebook to identify the strengths and weaknesses of each category of hospital.

In addition, a descriptive analysis item by item was conducted to assess the specific items for which the hospital websites failed and succeeded.

### Statistical analysis

The findings were analysed using a descriptive and a quantitative approach.

Table 1 shows the percentage of WWs for public and private hospitals stratified by region. Table 2 sets out the mean website evaluation score for each of the five sections of the Codebook and considers the category of hospital as an independent variable and the sectional mean website evaluation score as a dependent variable.

**Table 2:** Mean website evaluation score for each section of the Codebook, by category of hospital

	No. of items per section	Public hospitals type 1 (PubHs-1)#	Public hospitals type 2 (PubHs-2)#	Private hospitals (PrHs)	p* value
			
		mean percentage of items found			
1. Technical items	19	49.1 (5.3-89.5)	29.8 (10.5-63.1)	40.4 (10.5-73.7)	<0.001
2. Hospital information and facilities	22	50.0 (0-86.3)	22.6 (0-59.1)	35.6 (0-72.7)	<0.001
3. Admissions and medical services	25	40.6 (0-76.0)	26.0 (0-60.0)	32.3 (0-76.0)	<0.001
4. Interactive on-line services	10	23.6 (0-90.0)	10.3 (0-60.0)	25.4 (0-80.0)	<0.001
5. External activities	13	32.4 (0-92.3)	3.6 (0-69.2)	8.2 (0-61.5)	<0.001

All	89	41.9 (7.9-72.7)	21.2 (4.5-42.0)	30.8 (5.7-61.3)	<0.001

# PubHs-1: Public hospitals with their own website; PubHs-2: Public hospitals with a section on the website of their LHA

* Kruskal-Wallis one-way analysis of variance.

Tables 3, 4, 5, 6 and 7 present each individual item (dependent variable) for the category of hospital (independent variable).

**Table 3:** Percentage of hospitals presenting the specified item: technical items

Number and description of the item as it appears in the Codebook	Public hospitals type 1 (PubHs-1)# N = 198	Public hospitals type 2 (PubHs-2)# N = 221	Private hospitals (PrHs) N = 344	p* value	p* value PubH-1 vs PrHs
			
	% of hospitals reporting the item				
1. Site name appears on browser title bar	94.4	67.0	94.2	<0.001	0.91
2. Active part of the site appears on the browser title bar	44.9	27.0	34.9	0.01	0.02
3. Name of the hospital at the head of the website	97.5	53.8	96.8	<0.001	0.65
4. Hospital logo at the head of the website	91.4	3.6	90.7	<0.001	0.78
5. Any animation or visual displays can be bypassed	50.0	0	40.0	0.07	0.89
6. Access to the website in foreign languages	14.7	2.3	11.3	<0.001	0.26
7. Website map available	47.5	7.2	17.4	<0.001	<0.001
8. Website searcher available	61.6	4.5	27.9	<0.001	<0.001
9. Date of last website update	20.2	6.3	9.6	<0.001	<0.001
10. Website has HON (Health On the Net) foundation code certification	4.5	0	0	<0.001	<0.001
11. Website has certification of accessibility to people with disabilities (at least W3C WAI-A logo)	7.6	7.7	1.2	<0.001	<0.001
12. Website has certification of Cascading Style Sheets validation (W3C CSS logo)	13.6	15.8	3.5	<0.001	<0.001
13. Website has certification of Markup Validation Service (W3C HTML logo)	14.1	15.8	3.5	<0.001	<0.001
14. Website has certification of accessibility to people with disabilities provided by the Italian authority on informatics in the public administration	0	0.9	0	<0.001	-
15. Links with other useful websites provided (hospitals, scientific associations, institutions)	68.2	12.2	38.4	<0.001	<0.001
16. General disclaimers provided	15.7	7.7	9.3	0.02	0.03
17. Copyright notice	36.9	28.5	37.2	0.08	0.93
18. Treatment of surfer personal data statement	21.2	18.5	17.2	0.50	0.24
19. Website pages can be printed	83.8	90.5	80.5	0.01	0.37

# PubHs-1: Public hospitals with their own website; PubHs-2: Public hospitals with a section on the website of their LHA

* Chi-square test or Fisher exact test, when appropriate

**Table 4:** Percentage of hospitals presenting the specified item: hospital information and facilities

Number and description of the item as it appears in the Codebook	Public hospitals type 1 (PubHs-1)# N = 198	Public hospitals type 2 (PubHs-2)# N = 221	Private hospitals (PrHs) N = 344	p* value	p* value PubH-1 Vs PrHs
			
	% of hospitals reporting the item				
20. Hospital history	70.2	12.2	54.1	<0.001	<0.001
21. Contact details on the homepage or available at a click: hospital postal address	86.4	81.0	92.2	<0.001	0.03
22. Contact details on the homepage or available at a click: telephone and/or fax number	84.3	88.7	91.6	0.04	0.01
23. Contact details on the homepage or available at a click: e-mail address	67.2	30.8	79.4	<0.001	0.01
24. Contact details on the homepage or available at a click: VAT number	40.9	12.7	42.7	<0.001	0.68
25. Statement of purpose	64.1	7.2	53.5	<0.001	0.02
26. ISO certification on the homepage	13.6	0.9	23.0	<0.001	0.01
27. Organisation chart	68.7	19.9	30.8	<0.001	<0.001
28. Information regarding patient privacy	40.4	8.6	25.9	<0.001	<0.001
29. Ways of reaching the hospital: car, public transport	83.8	32.6	75.3	<0.001	0.02
30. Map of the hospital	41.9	11.8	12.2	<0.001	<0.001
31. Virtual visit to the hospital	11.1	0.5	18.3	<0.001	0.03
32. Public relations office: work hours	58.6	29.0	16.0	<0.001	<0.001
33. Public relations office: location	53.5	23.5	7.6	<0.001	<0.001
34. Public relations office: telephone and/or fax number	69.7	48.2	16.9	<0.001	<0.001
35. Public relations office: e-mail address	57.1	24.0	12.8	<0.001	<0.001
36. Services charter	58.1	7.7	34.0	<0.001	<0.001
37. Patient's rights and obligations	39.4	11.8	26.7	<0.001	0.01
38. Results of surveys regarding patient satisfaction are provided	3.5	2.7	1.7	0.42	0.20
39. Information for General Practitioners is provided	3.0	0.5	0.6	0.02	0.02
40. Information for foreigners is provided	19.2	8.1	2.3	<0.001	<0.001
41. Complementary services: press, cafeteria, television, telephone	66.7	34.8	66.3	<0.001	0.92

# PubHs-1: Public hospitals with their own website; PubHs-2: Public hospitals with a section on the website of their LHA

* Chi-square test or Fisher exact test, when appropriate

**Table 5:** Percentage of hospitals presenting the specified item: hospitalization and medical services

Number and description of the item as it appears in the Codebook	Public hospitals type 1 (PubHs-1)# N = 198	Public hospitals type 2 (PubHs-2)# N = 221	Private hospitals(PrHs) N = 344	p* value	p* value PubH-1 vs PrHs
			
	% of hospitals reporting the item				
42. Admission guide: different types of admissions are disclosed	71.7	45.2	72.1	<0.001	0.93
43. Admission guide: information and rules to be followed on admission	68.7	31.7	62.2	<0.001	0.13
44. Admission guide: information and rules to be followed during the hospital stay	55.1	19.5	43.9	<0.001	0.01
45. Admission guide: information and rules to be followed on discharge	48.5	16.7	40.1	<0.001	0.06
46. Admission guide: information and rules to be followed regarding visits by relatives	66.7	43.0	59.6	<0.001	0.10
47. Admission guide: information and procedure for obtaining a copy of the medical documentation	65.2	47.1	43.0	<0.001	<0.001
48. Details of how to pay prescription charges or fees	44.4	33.0	17.4	<0.001	<0.001
49. Departments or units providing user services: complete list	89.9	93.7	84.9	0.01	0.10
50. Departments or units providing user services: location	63.1	43.9	24.1	<0.001	<0.001
51. Departments or units providing user services: telephone and/or fax number and/or e-mail address	70.7	87.8	30.2	<0.001	<0.001
52. Detailed list of outpatient hospital services available (consultation, diagnostic services)	78.3	71.0	79.9	0.04	0.65
53. Number of hospital beds disclosed	51.5	24.9	66.0	<0.001	0.01
54. Waiting list disclosed	24.2	9.5	13.4	<0.001	0.01
55. Date of last monitoring of the waiting list disclosed	17.7	4.1	6.1	<0.001	<0.001
56. Hospital report of the number of admissions in the previous year	10.6	3.2	6.7	0.01	0.11
57. Doctors' curricula disclosed	12.1	1.4	8.7	<0.001	0.20
58. Hospital quality indicator: nosocomial infection rate disclosed	0	0	0.3	0.5	0.45
59. Hospital quality indicator: inpatient mortality rate disclosed	0.5	0	0.6	0.5	0.91
60. Hospital quality indicator: surgical mortality rate disclosed	0	0	0.6	0.3	0.28
61. Hospital quality indicator: others	11.6	1.4	13.4	<0.001	0.55
62. List of employed doctors in alphabetical order	9.6	0	4.1	<0.001	0.01
63. List of employed doctors by specialisation	47.5	31.7	45.6	<0.001	0.68
64. Information about private consultations/services and fees	69.2	28.5	63.4	<0.001	0.17
65. List of consultations/services with fees available	27.8	9.5	19.5	<0.001	0.03
66. Cost of consultations/services with fees available	10.1	4.5	1.5	<0.001	<0.001

# PubHs-1: Public hospitals with their own website; PubHs-2: Public hospitals with a section on the website of their LHA

* Chi-square test or Fisher exact test, when appropriate

**Table 6:** Percentage of hospitals presenting the specified item: interactive on-line services

Number and description of the item as it appears in the Codebook	Public hospitals type 1 (PubHs-1)# N = 198	Public hospitals type 2 (PubHs-2)# N = 221	Private hospitals(PrHs) N = 344	p* value	p* value PubH-1 vs PrHs
			
	% of hospitals reporting the item				
67. Appointments for consultation via the Internet	15.1	1.4	18.6	<0.001	0.31
68. Appointments for services/admission via the Internet	14.7	1.4	18.0	<0.001	0.31
69. Other facilities available via the Internet (e.g. documentation)	2.0	0.5	4.4	0.015	0.15
70. Appointments for consultation/services/admission via the Internet: link on the homepage	11.6	1.4	16.6	<0.001	0.12
71. Possibility to communicate with the hospital via the Internet or e-mail	79.8	48.4	85.2	<0.001	0.11
72. Possibility to ask a specialist a health- related question via the Internet or e-mail	6.1	0	8.1	<0.001	0.37
73. Information request form via the Internet or e-mail	75.2	47.1	82.8	<0.001	0.03
74. Suggestions/complaints form via the Internet or e-mail	17.2	2.7	10.2	<0.001	0.02
75. Possibility to sign up for a newsletter	13.1	0.5	8.4	<0.001	0.08
76. A health-related forum is present	1.5	0	1.5	0.20	0.95

# PubHs-1: Public hospitals with their own website; PubHs-2: Public hospitals with a section on the website of their LHA

* Chi-square test or Fisher exact test when appropriate

**Table 7:** Percentage of hospitals presenting the specified item: external activities

Number and description of the item as it appears in the Codebook	Public hospitals type 1 (PubHs-1)# N = 198	Public hospitals type 2 (PubHs-2)# N = 221	Private hospitals (PrHs) N = 344	p* value	p* value PubH-1 vs PrHs
			
	% of hospitals reporting the item				
77. Possibility to read online or to download health-care booklets	19.2	7.2	10.8	0.01	0.01
78. Medical glossary available	4.6	0.5	0.6	<0.001	0.01
79. Scientific studies that the hospital promotes or is involved in	36.9	3.7	8.7	<0.001	<0.001
80. Undergraduate or postgraduate courses that are held at the hospital	44.9	1.8	13.1	<0.001	<0.001
81. Presence of a library	28.8	1.4	1.7	<0.001	<0.001
82. Schedule of activities that take place at the hospital: courses, congresses and conferences	65.1	3.2	26.5	<0.001	<0.001
83. Publication of the hospital itself	26.8	0.5	8.1	<0.001	<0.001
84. Details of job opportunities at the hospital	63.1	6.3	17.4	<0.001	<0.001
85. Associations that work at the hospital: voluntary associations	46.0	11.8	5.5	<0.001	<0.001
86. Associations that work at the hospital: patient associations	27.3	1.8	1.4	<0.001	<0.001
87. Associations that work at the hospital: associations for the defence of patients' rights	17.2	7.2	0.9	<0.001	<0.001
88. Information on how to make a donation to the hospital	20.2	0	4.1	<0.001	<0.001
89. The hospital in the media: press review	20.7	0.9	7.8	<0.001	<0.001

# PubHs-1: Public hospitals with their own website; PubHs-2: Public hospitals with a section on the website of their LHA

* Chi-square test or Fisher exact test, when appropriate

The Kruskal-Wallis one-way analysis of variance and the Wilcoxon rank-sum test were used to investigate differences in the distribution of the scores calculated between and within hospital categories. The chi-square test and the Fisher exact test, when appropriate, were used for categorical variables. In the analysis of the distribution of each item in the three categories of hospital, both the p value calculated for the comparison of the three categories of hospital and the p value calculated for PubHs-1 versus PrHs are disclosed.

We rejected the null hypothesis below a p value of 0.05. All the analyses were conducted using the Stata statistical software package (version 10.0, Stata Corporation, College Station, Texas).

## Results

### Working websites of public hospitals and PrHs

The Italian National Health Service (INHS) comprises 1265 hospitals, as found in the IMH file, with vast differences in the total number and the proportion of public hospitals and PrHs between the 20 Italian regions. Table 1 shows a list of Italian regions with the corresponding number of hospitals, the percentage of WWs for public and private hospitals and the total number of hospitals.

We found that 419 of the 652 public hospitals (64.3%) and 344 of the 613 PrHs (56.1%) had a WW (p = 0.01). Six regions out of 20 (Calabria, Emilia-Romagna, Lombardy, Piedmont, Sardinia and Sicily) showed a statistically significant difference between public and private hospitals in terms of the percentage of WWs and only in one case, the Calabria region, was the proportion higher among PrHs.

### Mean website evaluation score per section of the Codebook

We calculated a mean website evaluation score for the five sections of the Codebook as shown in Table 2 (dependent variable), distributed by category of hospital (independent variable).

PubHs-1 scored better than the other categories in all sections except for "interactive on-line services". Indeed, in this section, PrHs scored 25.4, compared to 23.6 for PubHs-1, although the difference between these two categories was not statistically significant (p = 0.36). PubHs-2 provided the smallest amount of information in all the sections and their mean score ranged from 29.8 for "website technical items" to 3.6 for "external activities".

### Descriptive analysis of the items included in the 5 sections of the Codebook

A descriptive analysis, item by item for each section of the Codebook, is given in Tables 3, 4, 5, 6 and 7.

For each category of hospital, PubH-1, PubH-2 and PrH (independent variable), we assessed the percentage of hospital websites containing and not containing the single items (dependent variable) in the Codebook.

#### Section 1: technical items

Table 3 shows the percentage of hospital websites reporting the technical items.

The presence of different quality certification was assessed by identifying the corresponding logo on the home page (items 10-14). HON foundation certification (item 10) was present in only 4.5% of PubHs-1 and in no hospitals in the other two categories.

Certification of accessibility to people with disabilities (at least W3C WAI-A) (item 11) was present in less than 8% of the hospitals whereas certification of accessibility to people with disabilities by the Italian Authority on informatics in the public administration (item 14) was only present in two PubHs-2 (0.9%) and in none of the hospitals in the other two categories.

Two items concerned the accountability of the websites: the date of the last website update (item 9) was poorly reported, in 20.2% (40/198), 6.3% (14/221) and 9.6% (33/344) of the three categories (p < 0.001); no differences were found for the treatment of the surfers' personal data (item 18), present for nearly 20% of the hospitals.

#### Section 2: information and facilities

Table 4 details the 22 items on hospital information and facilities in section 2 of the Codebook.

All the hospitals gave a high percentage of contact details (items 21-24), more than 80%, but significantly more PrHs provided an address, telephone and/or fax number and e-mail address compared to PubHs-1 and PubHs-2.

Items 32-35 concerned the presence of a public relations office: more than 50% of PubHs-1 provided this information, whereas less than 17% of PrHs did so (p < 0.001).

Three items, 36-38, concerned the transparency of the hospital towards its users: in particular, the results of patient satisfaction surveys were provided by less than 4% of the hospitals, with no significant differences among categories.

#### Section 3: admissions and medical services

Table 5 shows the 25 items included in section 3 of the Codebook, concerning admissions and medical services.

The first six items, 42-47, concerned the availability of information regarding admissions, such as information and rules to be followed before, during and after the hospital stay. The different types of admission were disclosed in about 70% of PubHs-1 and PrHs, and in nearly 50% of PubHs-2 (item 42). A complete list of departments or units providing user services (item 49) was present in at least 85% of the hospitals, and a list of detailed outpatient hospital services, including consultation and diagnostic services, was present in at least 70% of the hospitals, both with no significant differences between private hospitals and PubHs-1.

Few hospitals disclosed the waiting list (item 54), 24.2% (48/198) of PubHs-1, 9.5% (21/221) of PubHs-2 and 13.4% (46/344) of PrHs (p = 0.01), and an even lower percentage gave the date on which the waiting list was last monitored, 17.7% (35/198), 4.1% (9/221) and 6.1% (21/344), respectively (p < 0.001).

The doctors' curricula were disclosed in only 12.2% (24/198), 1.4% (3/221) and 8.7% (30/344) of the three categories of hospital, respectively (p < 0.001). Information about hospital performance indicators was even rarer: only one hospital provided the nosocomial infection rate, two hospitals the inpatient mortality rate and three hospitals the surgical mortality rate, less than 1% of all the hospitals.

No difference was observed between PubHs-1 and PrHs with regard to information about private consultations or services (item 64), available in 69.2% (137/198) and 63.4% (218/344) of cases. Instead, some differences were found when we considered the cost of consultation or services available with fees: 10% of PubHs-1 (20/198) and only 1.5% of PrHs (5/344) provided this information (item 66, p < 0.001).

#### Section 4: interactive on-line services

This section comprised ten items, as shown in Table 6.

We established whether the Italian hospital websites allowed reservations via the Internet or e-mail: about 15% of PubHs-1 and 18% of PrHs accepted appointments for consultations or for services and admissions via the Internet (items 67 and 68). A higher percentage of hospitals offered the possibility to communicate (item 71), around 80% for both public hospitals and PrHs, or to obtain information about the hospital via the Internet or e-mail (item 73), 75.2% of PubHs-1 (149/198) and 82.8% of PrHs (285/344), p = 0.03. Very few hospitals, 6.1% of PubHs-1 (12/198), 8.1% of PrHs (28/344) and none of the PubHs-2, enabled users to ask a specialist a health-related question via the Internet or e-mail (item 72), and only 8 websites out of 763 (0.01%) - 5 PrHs (1.5%) and 3 PubHs-1 (1.5%) - provided a health-related forum (item 76).

#### Section 5: external activities

The last section of the Codebook regards the external activities of the hospitals and comprises 13 items, as shown in Table 7.

In this section PubHs-1 presented the highest percentage for all the items, the difference between the three categories of hospital and between PubHs-1 and PrHs being statistically significant.

The possibility of reading online or downloading health-care booklets (item 77) was present in 19.2% of PubHs-1 (38/198), 7.2% of PubHs-2 (16/221) and 10.8% of PrHs (37/344). As regards information about associations that work at the hospital, details of voluntary associations were present in 46.0% (91/198), 11.8% (26/221) and 5.5% (19/344) (item 85), patient associations in 27.3% (54/198), 1.8% (4/221) and 1.4% (5/344) (item 86) and associations for the defence of patients' rights in 17.2% (34/198), 7.2% (16/221) and 0.9% (3/344) (item 87).

## Discussion

This study examined the websites of all the Italian hospitals and documented that nearly the 40% of them do not have an official website. This percentage seems quite low considering that Norem, in a study of Norwegian hospital websites conducted in 2002, found 80% of websites available and this percentage is likely to have increased in recent years [16]. It looks as if many hospitals do not consider the web an important way of keeping in touch with their patients or enhancing visibility to potential users, although use of the Internet for health purposes has increased steadily in the last few years and this trend is likely to continue in the near future [5-8].

We found a significantly higher percentage of websites among public hospitals compared to private hospitals. Moreover, PubHs-1 scored better than PrHs, and PubHs-2 provided the least information. This could partly be due to the fact that PubHs-1 are very often the main hospitals in the town, with the highest number of beds, and about 10% of them are university hospitals.

This trend was confirmed when we calculated the score for each of the five sections of the Codebook, with the exception of the section concerning interactive on-line services, where PrHs scored the same as PubHs-1.

Regarding the technical characteristics, less than 15% of the hospital websites were available in a foreign language, English in most cases. This percentage appears very low, considering tourism, immigration and patients coming from other countries for health reasons, as has already been pointed out [16].

Very few hospitals, less than 7%, provided certification of website accessibility to people with disabilities. This finding is quite disappointing, especially because public institutions and private institutions providing services of public interest, such as health services, should have websites that are accessible to people with a disability.

Websites should provide the date of the last update for credibility reasons. In our study it was rarely reported, in less than 20% of PubHs-1 and 10% of PrHs. A study that compared the websites of top Spanish, American and British hospitals [27] found 12% of them disclosed the date of the last update, a result very similar to that reported by Kind (10%) [28], whereas Norwegian hospitals reached 40% [16].

As regards items concerning hospital information, contact details were provided by the great majority of hospitals but other items concerning a trust-based relationship between user and institution were much less likely to be reported. Only 19 out of 763 (2.5%) hospitals with a WW reported the results of surveys regarding patient satisfaction, there being no differences between PubHs-1, PubHs-2 and PrHs. Moreover, waiting lists were disclosed in a quarter of PubHs-1 and 13% of PrHs, and only 5 hospitals out of 763, less than 1%, provided specific clinical quality indicators, such as the nosocomial infection rate or inpatient and surgical mortality rates.

Information about consultations or services with fees was provided by more than 60% of PrHs and PubHs-1, but the costs of consultations and services with fees were only reported in 10% and 1.5%, respectively.

With regard to interactive on-line services, the results suggest that the Internet is used by hospitals as a means of communication, but real interaction between users and the institution appears far from being achieved. Indeed, the possibility of communicating via the Internet or e-mail was available in about 80% of PrHs and PubHs-1, but a form for asking a specialist health-related questions was available in less than 10% of the hospitals. A health-related forum was present for only 3 out of 198 PubHs-1 and 5 out of 344 PrHs, less then 1.5%. The use of new-generation Internet devices appears to be very limited on Italian hospital websites, which are more a source of information, from institution to users, than a way of participating and interacting with the institution's activities.

With regard to the results of the external activities of the websites, it is not surprising that PubHs-1 scored better than the rest. Compared to the other categories of hospital, PubHs-1 are closer to the academic and scientific environment, undergraduate or postgraduate courses being held in nearly half of them. They seem to be more integrated with the area they serve since voluntary and patient associations are present on many of their websites.

As regards the limitations of the study, many websites may have changed during the three-month survey as they are updated very quickly, or some hospitals without website may have gone on line.

We stratified the data on the basis of region and category of hospital (public versus private) mainly because the INHS is based on regionalization and competition between public and accredited private providers. It was not possible to stratify the results based on the number of hospital beds since this information was not available from an institutional source and few hospitals declared it on their websites. Other variables, however, such as specialized or general hospitals and university hospitals, are relevant for cross-sectional analysis and could be considered in future research.

Several considerations can be made regarding to the validity of the study, in terms of Codebook and intercoder reliability. As anticipated in the method section, the Codebook drawn up for this study attempted to integrate the items previously proposed by researchers studying hospital websites with new issues, especially the investigation of website user-orientation. We tested the Codebook in a preliminary study of 40 hospitals websites in order to assess its completeness in evaluating the characteristics of the website contents rather than informatics quality aspects. Again with regard to the study limitations, there are many ways to study a website and different criteria have already been proposed [15,16,21,22,27]. It is therefore possible that a different evaluation scale could have changed the results. However, the differences among the three hospital categories are consistent within all the sections of the Codebook, which suggests its internal coherence and face validity.

Five raters coded an equal number of websites to deal with the large number of hospitals found. The concordance study revealed a very good agreement among raters, hence a meeting to settle disagreements between raters preceded the final coding of the websites, although a certain variability in the coding cannot be ruled out.

As a methodological consideration, we used a descriptive approach since as far as we know there is no benchmark research in Italy in this field. This study can be therefore be considered a baseline survey at this time in the history of consumer health informatics literature in Italy.

Moreover, the proposed Codebook may provide a useful prototype for baseline surveys in countries where similar hospital-Internet diffusion evaluations have not occurred. It is difficult to generalize the study's findings to other countries because Italy has a unique health care organizational infrastructure and Internet usage patterns differ internationally. However, the Internet's dynamic nature suggests it is important to assess its use and potential by hospitals and medical care organizations in diverse nations. In this regard, the Codebook of this study may be adaptable for use in other countries regardless of their level of Internet and e-health acceptance, including ones where hospitals use Web 2.0 tools and ones where public access to the Internet and health information technology is emerging.

## Conclusions

This is the first study to examine the websites of all Italian hospitals. A high percentage of hospitals did not provide an official website; compared to private hospitals, public hospitals were more likely to have a website and more information. We found a greatly varying scenario, with a few excellent websites and others with poor quality and very scant information. These results suggest that the majority of Italian hospital websites have several limitations.

Very few hospitals provided information likely to increase the credibility of the hospital and user confidence in the institutions, as the results of surveys regarding patient satisfaction and clinical quality indicators point out.

These indicators, such as standardised hospital mortality rate, are increasingly being used to compare hospital performance and to satisfy public demand for transparency. The UK National Health Service [29] and the French Ministry of Health give examples that provide these data in clear and easy-to-use databases [30].

It would appear that Italian hospitals, even leading ones, are not interested in promoting their performance, even if they detail the medical services they offer. This could be due to the fact that patients are still using other methods for choosing hospitals; advice from friends and relatives, word of mouth and the general practitioner still seem to play a major role in choosing the best place to be treated. This study also indicates that Italian hospital websites remain more a source of information on admissions and services than an interactive platform for communication between users and hospitals, since interactive tools, such as a health-related forum and the possibility to sign up for a newsletter, are virtually absent.

Public health institutions should not ignore these emerging means of communication, especially in terms of health promotion. As there is a huge amount of health information available outside institutional channels, hospitals, like other health institutions, should provide reliable information via their websites and promote contents that can get ahead of potentially harmful advertising [31].

## Abbreviations

LHA: Local Health Authority; INHS: Italian National Health Service; PubHs-1: Public hospitals with their own website; PubHs-2: Public hospitals with a section on the website of their Local Health Authority; PrHs: Private hospitals.

## Competing interests

The authors declare that they have no competing interests.

## Authors' contributions

GM developed the study design, conducted the statistical analysis and drafted the manuscript. GO assisted with data interpretation and drafted the manuscript.  MB, SD, CG, EP were involved in data acquisition and assisted with data interpretation. LC and PJS reviewed the manuscript critically and gave final approval of the version to be published.  UG developed the study design, supervised the study and reviewed the manuscript critically. All the authors read and approved the final manuscript.

## Pre-publication history

The pre-publication history for this paper can be accessed here:

http://www.biomedcentral.com/1472-6947/10/17/prepub

## Supplementary Material

Additional file 1 **List of all Italian hospitals updated to 1st January 2008**. The list of all Italian hospitals has been obtained from the Italian Ministry of Health website (modified as regards the column headings). The original file is available at: http://www.ministerosalute.it/servizio/documenti/INDIRIZZO_STRUTTURE_1_gennaio_2008.xls. Last accessed 2 July 2009. Click here for file

Additional file 2 **Codebook**. List of the eighty-nine items included in the study. Click here for file
