# Supplementary material for: Italian hospitals on the web: a cross-sectional analysis of official websites
Source: BMC Med Inform Decis Mak. 2010 Apr 1;10:17. doi: 10.1186/1472-6947-10-17 (PMC2868065; doi:10.1186/1472-6947-10-17)
Supplement: Additional file 1 — List of all Italian hospitals updated to 1st January 2008. The list of all Italian hospitals has been obtained from the Italian Ministry of Health website (modified as regards the column headings). The original file is available at: http://www.ministerosalute.it/servizio/documenti/INDIRIZZO_STRUTTURE_1_gennaio_2008.xls. Last accessed 2 July 2009. [file 1472-6947-10-17-S1.PDF]

Appendix 1: List of all Italian hospitals updated to 1st January 2008 as obtained from the Italian Ministry of Health website (modified by the Authors)

Available at:

http://www.ministerosalute.it/servizio/documenti/INDIRIZZO_STRUTTURE_1_gennaio_2008.xls

Last accessed 2 July 2009

N	Regional code	Region	Hospital code (unambiguous code)	Name of the hospital	Address	Post code	Town	Public or private hospital
1	010	PIEMONTE	10007	OSPEDALE MARTINI	VIA TOFANE	10100	TORINO	PUBLIC
2	010	PIEMONTE	10010	OSPEDALE OFTALMICO	VIA JUVARRA FILIPPO	10100	TORINO	PUBLIC
3	010	PIEMONTE	10012	OSPEDALE EVANGELICO VALDESE	VIA PELLICO SILVIO	10100	TORINO	PUBLIC
4	010	PIEMONTE	10601	CLINICA PINNA PINTOR	VIA VESPUCCI AMERIGO	10100	TORINO	PRIVATE
5	010	PIEMONTE	10603	CASA DI CURA SUORE DOMENICANE	VIA VILLA DELLA REGINA	10100	TORINO	PRIVATE
6	010	PIEMONTE	10604	CASA DI CURA "SEDES SAPIENTIAE"	VIA BIDONE GIORGIO	10100	TORINO	PRIVATE
7	010	PIEMONTE	10610	CASA DI CURA FORNACA DISESSANT	CORSO VITTORIO EMANUELE II	10100	TORINO	PRIVATE
8	010	PIEMONTE	10612	CASA DI CURA KOELLIKER OSPEDALINO	CORSO FERRARIS GALILEO	10100	TORINO	PRIVATE
9	010	PIEMONTE	10653	PROMEA S.P.A.	VIA MENABREA FEDERICO	10100	TORINO	PRIVATE
10	010	PIEMONTE	10655	CASA DI CURA CELLINI	VIA CELLINI BENVENUTO	10100	TORINO	PRIVATE
11	010	PIEMONTE	10662	MOD CENTRO MEDICO CHIRURGICO	CORSO D'AZEGLIO MASSIMO	10126	TORINO	PRIVATE
12	010	PIEMONTE	10664	MEDICENTER S.R.L.	VIA FREIDOUR	10139	TORINO	PRIVATE
13	010	PIEMONTE	10893	PRESIDIO SANITARIO SAN CAMILLO	STRADA COMUNALE SANTA MARGHERITA	10100	TORINO	PUBLIC
14	010	PIEMONTE	10003	OSPEDALE MARIA VITTORIA	VIA CIBRARIO LUIGI	10144	TORINO	PUBLIC
15	010	PIEMONTE	10011	TORINO NORD EMERGENZA SAN GIOVANNI BOSCO	PIAZZA DEL DONATORE DI SANGUE	10154	TORINO	PUBLIC
16	010	PIEMONTE	10013	OSPEDALE AMEDEO DI SAVOIA	CORSO SVIZZERA	10149	TORINO	PUBLIC
17	010	PIEMONTE	10607	CASA DI CURA MAJOR	VIA SANTA GIULIA	10100	TORINO	PRIVATE
18	010	PIEMONTE	10611	VILLA MARIA PIA HOSPITAL	STRADA COMUNALE DI MONGRENO	10132	TORINO	PRIVATE
19	010	PIEMONTE	10613	CASA DI CURA VILLA CRISTINA	STRADA DELLE VALLETTE	10100	TORINO	PRIVATE
20	010	PIEMONTE	10890	PRESIDIO SANITARIO AUSILIATRICE - FONDAZ	VIA PEYRON AMEDEO	10100	TORINO	PUBLIC
21	010	PIEMONTE	10892	PRESIDIO SANITARIO GRADENIGO	CORSO REGINA MARGHERITA	10100	TORINO	PUBLIC
22	010	PIEMONTE	10897	PRESIDIO SANITARIO OSPEDALE COTTOLENGO	VIA SAN GIUSEPPE BENEDETTO COTTOLEN(	10100	TORINO	PUBLIC
23	010	PIEMONTE	10030	OSPEDALE CIVILE "E.AGNELLI"	VIA BRIGATA CAGLIARI	10064	PINEROLO	PUBLIC
24	010	PIEMONTE	10079	OSPEDALI RIUNITI - A.S.L. 5 - COLLEGNO	VIA RIVALTA	10098	RIVOLI	PUBLIC
25	010	PIEMONTE	10079	OSPEDALI RIUNITI - A.S.L. 5 - COLLEGNO	VIA RIVALTA	10098	RIVOLI	PUBLIC
26	010	PIEMONTE	10079	OSPEDALI RIUNITI - A.S.L. 5 - COLLEGNO	VIA RIVALTA	10098	RIVOLI	PUBLIC
27	010	PIEMONTE	10079	OSPEDALI RIUNITI - A.S.L. 5 - COLLEGNO	VIA RIVALTA	10098	RIVOLI	PUBLIC
28	010	PIEMONTE	10079	OSPEDALI RIUNITI - A.S.L. 5 - COLLEGNO	VIA RIVALTA	10098	RIVOLI	PUBLIC
29	010	PIEMONTE	10614	CASA DI CURA VILLE AUGUSTA - S.R.L.	VIA PINEROLO SUSA	10090	BRUINO	PRIVATE
30	010	PIEMONTE	10615	CASA DI CURA VILLA PATRIZIA - S.R.L.	REGIONE GIORDA	10045	PIOSSASCO	PRIVATE
31	010	PIEMONTE	10621	CASA DI CURA VILLA SERENA - S.P.A.	VIA MAGENTA	10045	PIOSSASCO	PRIVATE
32	010	PIEMONTE	10623	VILLA PAPA GIOVANNI XXIII	VIA SAN GILLIO	10044	PIANEZZA	PRIVATE
33	010	PIEMONTE	10646	CASA DI CURA VILLA IRIS SRL	VIA PAVESE CESARE	10044	PIANEZZA	PRIVATE
34	010	PIEMONTE	10651	CASA DI CURA MADONNA DEI BOSCHI	VIA RAGAZZI DEL ' 99	10090	BUTTIGLIERA ALTA	PRIVATE
35	010	PIEMONTE	10663	MEDICAL SERVICE S.A.S	VIA MARTIRI DEL XXI	10064	PINEROLO	PRIVATE
36	010	PIEMONTE	10017	OSPEDALE CIVICO CHIVASSO	CORSO FERRARIS GALILEO	10034	CHIVASSO	PUBLIC
37	010	PIEMONTE	10019	PRESIDI OSPED. RIUNITI A.S.L. 6 CIRIIE'	VIA BATTITORE	10073	CIRIE`	PUBLIC
38	010	PIEMONTE	10019	PRESIDI OSPED. RIUNITI A.S.L. 6 CIRIIE'	VIA BATTITORE	10073	CIRIE`	PUBLIC
39	010	PIEMONTE	10023	OSPEDALI RIUNITI DEL CANAVESE	PIAZZA DELLA CREDENZA	10015	IVREA	PUBLIC
40	010	PIEMONTE	10023	OSPEDALI RIUNITI DEL CANAVESE	PIAZZA DELLA CREDENZA	10015	IVREA	PUBLIC
41	010	PIEMONTE	10023	OSPEDALI RIUNITI DEL CANAVESE	PIAZZA DELLA CREDENZA	10015	IVREA	PUBLIC
42	010	PIEMONTE	10616	CASA DI CURA CLINICA EPOREDIESE	VIA DELLA CASTIGLIA	10015	IVREA	PRIVATE

43	010	PIEMONTE	10617	VILLE "TURINA AMIONE"	PIAZZA VITTIME DI BOLOGNA	10077	SAN MAURIZIO CANAVESE	PRIVATE
44	010	PIEMONTE	10619	LA BERTALAZONA CASA DI CURA	VIA BERTALAZONE	10077	SAN MAURIZIO CANAVESE	PRIVATE
45	010	PIEMONTE	10622	VILLA IDA	VIA CHALLANT AYMONE	10074	LANZO TORINESE	PRIVATE
46	010	PIEMONTE	10644	VILLA GRAZIA	STRADA POLIGONO	10070	SAN CARLO CANAVESE	PRIVATE
47	010	PIEMONTE	10891	BEATA VERGINE CONSOLATA	VIA FATEBENEFRATELLI	10077	SAN MAURIZIO CANAVESE	PUBLIC
48	010	PIEMONTE	10004	OSPEDALI RIUNITI A.S.L. 8	VIA DE MARIA	10023	CHIERI	PUBLIC
49	010	PIEMONTE	10004	OSPEDALI RIUNITI A.S.L. 8	VIA DE MARIA	10023	CHIERI	PUBLIC
50	010	PIEMONTE	10004	OSPEDALI RIUNITI A.S.L. 8	VIA DE MARIA	10023	CHIERI	PUBLIC
51	010	PIEMONTE	10004	OSPEDALI RIUNITI A.S.L. 8	VIA DE MARIA	10023	CHIERI	PUBLIC
52	010	PIEMONTE	10618	CASA DI CURA E RIPOSO S. LUCA S.P.A.	STRADA DELLA VETTA	10020	PECETTO TORINESE	PRIVATE
53	010	PIEMONTE	10620	CASA DI CURA VILLA DI SALUTE	VIALE DELLA RESISTENZA	10028	TROFARELLO	PRIVATE
54	010	PIEMONTE	10648	CASA DI CURA VILLA ADRIANA	VIA ROBIOLA	10020	ARIGNANO	PRIVATE
55	010	PIEMONTE	10661	LISA S.R.L. AMBULATORIO POLISPECIALISTIC	VIA RACCONIGI	10022	CARMAGNOLA	PRIVATE
56	010	PIEMONTE	10009	OSPEDALE UNICO DEL VERCELLESE	CORSO ABBIATE MARIO	13100	VERCELLI	PUBLIC
57	010	PIEMONTE	10009	OSPEDALE UNICO DEL VERCELLESE	CORSO ABBIATE MARIO	13100	VERCELLI	PUBLIC
58	010	PIEMONTE	10009	OSPEDALE UNICO DEL VERCELLESE	CORSO ABBIATE MARIO	13100	VERCELLI	PUBLIC
59	010	PIEMONTE	10626	POL.DI MONZA CASA C.P. PRESIDIO.S. RITA	VIALE DELLA AERONAUTICA	13100	VERCELLI	PRIVATE
60	010	PIEMONTE	10657	CASA DI CURA CENTRO R.R.F. MONS. LUIGI N	VIA MARTIRI DELLA LIBERTA'	13040	MONCRIVELLO	PRIVATE
61	010	PIEMONTE	10085	OSP.DEGLI INFERMI DI BIELLA	VIA CARACCIO RODOLFO	13900	BIELLA	PUBLIC
62	010	PIEMONTE	10627	CASA DI CURA SAN GIORGIO	VIA GATTINARA	13886	VIVERONE	PRIVATE
63	010	PIEMONTE	10656	LA VIALARDA SPA	VIA RAMELLA GERMANIN ERIBERTO	13900	BIELLA	PRIVATE
64	010	PIEMONTE	10006	OSPEDALI RIUNITI ASL 13 NOVARA	VIALE ZOPPIS	28021	BORGOMANERO	PUBLIC
65	010	PIEMONTE	10006	OSPEDALI RIUNITI ASL 13 NOVARA	VIALE ZOPPIS	28021	BORGOMANERO	PUBLIC
66	010	PIEMONTE	10628	CASA DI CURA S.GAUDENZIO	VIA BOTTINI ENRICO	28100	NOVARA	PRIVATE
67	010	PIEMONTE	10630	CASA DI CURA S.CARLO DI ARONA	VIA OLEGGIO CASTELLO	28041	ARONA	PRIVATE
68	010	PIEMONTE	10649	CASA DI CURA I CEDRI	LARGO DON GUANELLA	28073	FARA NOVARESE	PRIVATE
69	010	PIEMONTE	10660	CENTRO MEDICO POLISPECIALISTICO AMBULATO	VIA PIAVE	28021	BORGOMANERO	PRIVATE
70	010	PIEMONTE	10005	OSPEDALI RIUNITI A.S.L. 14	VIA MAZZINI GIUSEPPE	28887	OMEGNA	PUBLIC
71	010	PIEMONTE	10005	OSPEDALI RIUNITI A.S.L. 14	VIA MAZZINI GIUSEPPE	28887	OMEGNA	PUBLIC
72	010	PIEMONTE	10005	OSPEDALI RIUNITI A.S.L. 14	VIA MAZZINI GIUSEPPE	28887	OMEGNA	PUBLIC
73	010	PIEMONTE	10629	CASA DI CURA L'EREMO DI MIAZZINA	VIA PER MIAZZINA	28814	CAMBIASCA	PRIVATE
74	010	PIEMONTE	10896	CENTRO ORTOPEDICO DI QUADRANTE	LUNGO LAGO BUOZZI	28887	OMEGNA	PUBLIC
75	010	PIEMONTE	10014	PRESIDIO DI CARAGLIO	VIA VALGRANA	12023	CARAGLIO	PUBLIC
76	010	PIEMONTE	10124	PRESIDIO A.S.L. 16	VIA DELL' OSPEDALE	12084	MONDOVI`	PUBLIC
77	010	PIEMONTE	10124	PRESIDIO A.S.L. 16	VIA DELL' OSPEDALE	12084	MONDOVI`	PUBLIC
78	010	PIEMONTE	10126	OSPEDALI RIUNITI ASL 17	VIA OSPEDALI	12038	SAVIGLIANO	PUBLIC
79	010	PIEMONTE	10126	OSPEDALI RIUNITI ASL 17	VIA OSPEDALI	12038	SAVIGLIANO	PUBLIC
80	010	PIEMONTE	10126	OSPEDALI RIUNITI ASL 17	VIA OSPEDALI	12038	SAVIGLIANO	PUBLIC
81	010	PIEMONTE	10633	ISTITUTO CLIMATICO DI ROBILANTE	STRADA VALLONE FANTINO	12017	ROBILANTE	PRIVATE
82	010	PIEMONTE	10634	CASA DI CURA MONTESERRAT S.R.L	STRADA TETTO SANT'ANTONIO ARADOLO	12011	BORGO SAN DALMAZZO	PRIVATE
83	010	PIEMONTE	10647	CASA DI CURA "STELLA DEL MATTINO"	VIA MELLANA	12012	BOVES	PRIVATE
84	010	PIEMONTE	10659	S.R.L. TERAPEUTICENTER - ISTITUTO MEDICO	STRADA CARMAGNOLA	12030	CARAMAGNA PIEMONTE	PRIVATE
85	010	PIEMONTE	10120	OSP. S.LAZZARO-ALBA E S.SPRITO BRA	VIA BELLI PIERINO	12051	ALBA	PUBLIC
86	010	PIEMONTE	10120	OSP. S.LAZZARO-ALBA E S.SPRITO BRA	VIA BELLI PIERINO	12051	ALBA	PUBLIC
87	010	PIEMONTE	10631	CASA DI CURA SAN MICHELE	STRADA SAN MICHELE	12042	BRA	PRIVATE
88	010	PIEMONTE	10632	CASA DI CURA CITTA' DI BRA	STRADA MONTENERO	12042	BRA	PRIVATE
89	010	PIEMONTE	10635	CASA DI CURA "LA RESIDENZA"	VIA ROMA	12050	RODELLO	PRIVATE
90	010	PIEMONTE	10008	OSPEDALI RIUNITI ASL 19	CORSO DANTE	14100	ASTI	PUBLIC
91	010	PIEMONTE	10008	OSPEDALI RIUNITI ASL 19	CORSO DANTE	14100	ASTI	PUBLIC
92	010	PIEMONTE	10638	CASA DI CURA S.GIUSEPPE SPA	VIA DE GASPERI ALCIDE	14100	ASTI	PRIVATE
93	010	PIEMONTE	10639	CASA DI CURA S. ANNA SPA	VIA AUBERT PIETRO	14100	ASTI	PRIVATE
94	010	PIEMONTE	10654	CENTRO CHIRURGICO GUTTUARI	VIA GUTTUARI	14100	ASTI	PRIVATE
95	010	PIEMONTE	10015	OSPEDALI RIUNITI ASL 21	VIALE GIOLITTI GIOVANNI	15033	CASALE MONFERRATO	PUBLIC
96	010	PIEMONTE	10015	OSPEDALI RIUNITI ASL 21	VIALE GIOLITTI GIOVANNI	15033	CASALE MONFERRATO	PUBLIC
97	010	PIEMONTE	10165	OSPEDALI RIUNITI ASL 22	VIA RAGGIO EDILIO	15067	NOVI LIGURE	PUBLIC
98	010	PIEMONTE	10165	OSPEDALI RIUNITI ASL 22	VIA RAGGIO EDILIO	15067	NOVI LIGURE	PUBLIC
99	010	PIEMONTE	10165	OSPEDALI RIUNITI ASL 22	VIA RAGGIO EDILIO	15067	NOVI LIGURE	PUBLIC
100	010	PIEMONTE	10166	OSPEDALE SS ANTONIO E MARGHERITA	PIAZZA CAVALLOTTI FELICE	15057	TORTONA	PUBLIC
101	010	PIEMONTE	10640	CASA DI CURA SALUS SRL	VIA TROTTI	15100	ALESSANDRIA	PRIVATE
102	010	PIEMONTE	10641	CASA DI CURA VILLA IGEA	STRADA MOIRANO	15011	ACQUI TERME	PRIVATE
103	010	PIEMONTE	10642	CASA DI CURA SANT'ANNA	VIALE CAVALLI D'OLIVOLA	15033	CASALE MONFERRATO	PRIVATE
104	010	PIEMONTE	10643	CASA DI CURA CITTA' DI ALESSANDRIA	VIA BUOZZI BRUNO	15100	ALESSANDRIA	PRIVATE
105	010	PIEMONTE	10901	AZIENDA OSPED.S.GIOVANNI BATTISTA DI TOR	CORSO LAZZARI FRANCESCO DETTO IL BRA	10100	TORINO	PUBLIC

106	010	PIEMONTE	10901	AZIENDA OSPED.S.GIOVANNI BATTISTA DI TOR	CORSO LAZZARI FRANCESCO DETTO IL BRA	10100	TORINO	PUBLIC
107	010	PIEMONTE	10901	AZIENDA OSPED.S.GIOVANNI BATTISTA DI TOR	CORSO LAZZARI FRANCESCO DETTO IL BRA	10100	TORINO	PUBLIC
108	010	PIEMONTE	10902	C.T.O.-C.R.F.-MARIA ADELAIDE	VIA ZURETTI GIANFRANCO	10126	TORINO	PUBLIC
109	010	PIEMONTE	10902	C.T.O.-C.R.F.-MARIA ADELAIDE	VIA ZURETTI GIANFRANCO	10126	TORINO	PUBLIC
110	010	PIEMONTE	10903	AZIENDA OSPEDALIERA O.I.R.M.S.-SANT'ANNA	CORSO SPEZIA	10100	TORINO	PUBLIC
111	010	PIEMONTE	10903	AZIENDA OSPEDALIERA O.I.R.M.S.-SANT'ANNA	CORSO SPEZIA	10100	TORINO	PUBLIC
112	010	PIEMONTE	10904	AZ.SAN.OSPEDALIERA 'S.LUIGI'	REGIONE GONZOLE	10043	ORBASSANO	PUBLIC
113	010	PIEMONTE	10905	AZIENDA OSPED. NOVARA E GALLIATE	CORSO MAZZINI GIUSEPPE	28100	NOVARA	PUBLIC
114	010	PIEMONTE	10905	AZIENDA OSPED. NOVARA E GALLIATE	CORSO MAZZINI GIUSEPPE	28100	NOVARA	PUBLIC
115	010	PIEMONTE	10906	AZ. OSPEDAL. S. CROCE E CARLE	VIA COPPINO MICHELE	12100	CUNEO	PUBLIC
116	010	PIEMONTE	10907	AZ. SS.ANTONIO E BIAGIO E C.ARRIGO	VIA VENEZIA	15100	ALESSANDRIA	PUBLIC
117	010	PIEMONTE	10907	AZ. SS.ANTONIO E BIAGIO E C.ARRIGO	VIA VENEZIA	15100	ALESSANDRIA	PUBLIC
118	010	PIEMONTE	10907	AZ. SS.ANTONIO E BIAGIO E C.ARRIGO	VIA VENEZIA	15100	ALESSANDRIA	PUBLIC
119	010	PIEMONTE	10908	AZIENDA OSPEDALIERA ORDINE MAURIZIANO DI	LARGO TURATI FILIPPO	10128	TORINO	PUBLIC
120	010	PIEMONTE	10908	AZIENDA OSPEDALIERA ORDINE MAURIZIANO DI	LARGO TURATI FILIPPO	10128	TORINO	PUBLIC
121	010	PIEMONTE	10920	CENTRO AUXOLOGICO ITALIANO	VIA CADORNA	28824	OGGEBBIO	PUBLIC
122	010	PIEMONTE	10921	FONDAZIONE SALVATORE MAUGERI	VIA REVISLATE	28010	VERUNO	PUBLIC
123	020	VALLE D`AOSTA	20001	OSPEDALE GENERALE REGIONALE	VIALE GINEVRA 3	11100	AOSTA	PUBLIC
124	030	LOMBARDIA	30143	CLINICHE GAVAZZENI SPA - BERGAMO	VIA M. GAVAZZENI 21	24125	BERGAMO	PRIVATE
125	030	LOMBARDIA	30144	CASA DI CURA BEATO PALAZZOLO - BERGAMO	VIA S.BERNARDINO 56	24122	BERGAMO	PRIVATE
126	030	LOMBARDIA	30145	CASA DI CURA CASTELLI - BERGAMO	VIA MAZZINI 11	24128	BERGAMO	PRIVATE
127	030	LOMBARDIA	30146	CASA DI CURA S. FRANCESCO - BERGAMO	VIA IV NOVEMBRE 7	24128	BERGAMO	PRIVATE
128	030	LOMBARDIA	30147	POLICLINICO SAN MARCO S.R.L.-OSIO SOTTO	CORSO EUROPA 7	24040	OSIO SOTTO	PRIVATE
129	030	LOMBARDIA	30148	POLICLINICO SAN PIETRO S.P.A.	VIA FORLANINI,15	24036	PONTE SAN PIETRO	PRIVATE
130	030	LOMBARDIA	30149	CASA DI CURA QUARENGHI - S.PELLEGRINO T.	VIA SAN CARLO 70	24016	SAN PELLEGRINO TERME	PRIVATE
131	030	LOMBARDIA	30337	VILLA SANT'APOLLONIA S.R.L. - BERGAMO	VIA G. MOTTA 37/39	24123	BERGAMO	PRIVATE
132	030	LOMBARDIA	30345	CENTRO MEDICO DI RIABIL.POLIFUNZ.MULTIF.	VIA FACCANONI,6 - C/O OSP. FACCANONI	24067	SARNICO	PRIVATE
133	030	LOMBARDIA	30350	OSPEDALE S.ISIDORO - TRESCORE B.	VIA OSPEDALE, 34	24069	TRESCORE BALNEARIO	PRIVATE
134	030	LOMBARDIA	30354	U.O.NEFROLOGIA-1-BOLOGNINI-SERIATE	VIA PADERNO, 21	24068	SERIATE	PRIVATE
135	030	LOMBARDIA	30356	ISTITUTO CLINICO HABILITA	VIA BOLOGNA, 1	24040	CISERANO	PRIVATE
136	030	LOMBARDIA	30360	FERB - ONLUS CENTRO ALZHEIMER-GAZZANIGA	VIA MANZONI, 130	24025	GAZZANIGA	PRIVATE
137	030	LOMBARDIA	30361	CASA DI CURA SAN DONATO SRL	VIA CAVOUR, 6/A	24046	OSIO SOTTO	PRIVATE
138	030	LOMBARDIA	30171	OSPEDALE S. ORSOLA F.B.F. - BRESCIA	VIA VITTORIO EMANUELE II, 27	25122	BRESCIA	PUBLIC
139	030	LOMBARDIA	30174	CASA SALUTE MORO GIRELLI FOND.D.GNOCCHI	VIA FRANCESCO CRISPI, 21/24	25121	BRESCIA	PRIVATE
140	030	LOMBARDIA	30175	CASA DI CURA S. CAMILLO - BRESCIA	VIA FILIPPO TURATI N.44	25123	BRESCIA	PRIVATE
141	030	LOMBARDIA	30176	CASA DI CURA S. ANNA - BRESCIA	VIA DEL FRANZONE 31	25127	BRESCIA	PRIVATE
142	030	LOMBARDIA	30177	CASA DI CURA VILLA GEMMA-GARDONE RIVIERA	VIA ZANARDELLI 101	25083	GARDONE RIVIERA	PRIVATE
143	030	LOMBARDIA	30178	IST.CLIN. CITTA' DI BRESCIA SPA-BRESCIA	VIA B. GUALLA, 15	25123	BRESCIA	PRIVATE
144	030	LOMBARDIA	30275	CLINICA S. ROCCO DI FRANCIACORTA - OME	VIA DEI SABBIONI,24	25050	OME	PRIVATE
145	030	LOMBARDIA	30294	DOMUS SALUTIS - BRESCIA	VIA LAZZARETTO N.3	25123	BRESCIA	PRIVATE
146	030	LOMBARDIA	30295	POLIAMBULANZA - BRESCIA	VIA BISSOLATI N.57	25124	BRESCIA	PRIVATE
147	030	LOMBARDIA	30348	CASA DI CURA VILLA BARBARANO	VIA SPIAGGIA D'ORO, 17	25087	SALO`	PRIVATE
148	030	LOMBARDIA	30362	RESIDENZA ANNI AZZURRI	VIA SBERNA, 4/6	25086	REZZATO	PRIVATE
149	030	LOMBARDIA	30366	DOMINATO LEONENSE SANITA'	VIA GARIBALDI, 25	25024	LENO	PRIVATE
150	030	LOMBARDIA	30368	CENTRO RIAB.E.SPALENZA-FOND.DON GNOCCHI	VIA GOLGI, 1	25038	ROVATO	PRIVATE
151	030	LOMBARDIA	30369	CENTRO MEDICO RICHIEDEI - PALAZZOLO S/O	VIA SGRAZZUTTI, 20	25064	PALAZZOLO SULL`OGLIO	PRIVATE
152	030	LOMBARDIA	30029	OSP.SACRA FAMIGLIA - F.B.F. - ERBA	VIA FATEBENEFRATELLI 20	22036	ERBA	PUBLIC
153	030	LOMBARDIA	30030	OSP.GENERALE DI ZONA VALDUCE - COMO	VIA DANTE ALIGHIERI 11	22100	COMO	PUBLIC
154	030	LOMBARDIA	30031	OSP. MORIGGIA PELASCINI - GRAVEDONA	VIA PELASCINI 3	22015	GRAVEDONA	PUBLIC
155	030	LOMBARDIA	30032	IST.CLINICO VILLA APRICA SPA-COMO	VIA CASTELCARNASINO 10	22100	COMO	PRIVATE
156	030	LOMBARDIA	30035	C.O.F. LANZO HOSPITAL S.P.A.	LOCALITA' MONTE CASLE', 5	22024	RAMPONIO VERNA	PRIVATE
157	030	LOMBARDIA	30037	CASA DI CURA VILLA S. BENEDETTO - ALBESE	VIA ROMA 16	22032	ALBESE CON CASSANO	PRIVATE
158	030	LOMBARDIA	30038	CASA DI CURA LE BETULLE-APPIANO GENTILE	VIALE ITALIA 36	22070	APPIANO GENTILE	PRIVATE
159	030	LOMBARDIA	30347	CASA DI CURA VILLA S. GIUSEPPE	VIA VILLA S.GIUSEPPE, 1	22040	ANZANO DEL PARCO	PRIVATE
160	030	LOMBARDIA	30372	CENTRO DIAGNOSTICO SAN NICOLO' - S.R.L.	VIALE INNOCENZO XI, 70	22100	COMO	PRIVATE
161	030	LOMBARDIA	30217	C.C. ANCELLE DELLA CARITA'-CREMONA	VIA ASELLI, 14	26100	CREMONA	PRIVATE
162	030	LOMBARDIA	30218	CASA DI CURA S. CAMILLO - CREMONA	VIA MANTOVA, 113	26100	CREMONA	PRIVATE
163	030	LOMBARDIA	30219	CASA DI CURA FIGLIE DI S.CAMILLO-CREMONA	VIA F. FILZI 56	26100	CREMONA	PRIVATE
164	030	LOMBARDIA	30033	CASA DI CURA BEATO L.TALAMONI-LECCO	VIA SAN NICOLO' 12	23900	LECCO	PRIVATE
165	030	LOMBARDIA	30034	CASA DI CURA G.B. MANGIONI - LECCO	VIA LEONARDO DA VINCI 49	23900	LECCO	PRIVATE
166	030	LOMBARDIA	30284	PRESIDIO DI RIABIL.VILLA BERETTA-COSTA M	VIA NAZARIO SAURO, 17	23845	COSTA MASNAGA	PUBLIC
167	030	LOMBARDIA	30259	CASA DI CURA S. CLEMENTE - MANTOVA	V.LE POMPILIO 65	46100	MANTOVA	PRIVATE
168	030	LOMBARDIA	30353	OSPEDALE CIVILE DI VOLTA MANTOVANA	VIA TONELLO, 5	46049	VOLTA MANTOVANA	PRIVATE

169	030	LOMBARDIA	30355	OSPEDALE DI SUZZARA S.P.A.	VIA G. CANTORE, 14/B	46029	SUZZARA	PRIVATE
170	030	LOMBARDIA	30357	OSP. SAN PELLEGRINO - CASTIGLIONE D/S	VIA GARIBALDI, 65	46043	CASTIGLIONE DELLE STIVIERE	PRIVATE
171	030	LOMBARDIA	30095	CASA DI CURA CITTA' DI MILANO - MILANO	VIA LAMARMORA 10	20122	MILANO	PRIVATE
172	030	LOMBARDIA	30096	CASA DI CURA DEL POLICLINICO - MILANO	VIA G.DEZZA, 48	20144	MILANO	PRIVATE
173	030	LOMBARDIA	30097	CASA DI CURA S. RITA - MILANO	VIA CATALANI 4	20131	MILANO	PRIVATE
174	030	LOMBARDIA	30100	CASA DI CURA IGEA - MILANO	VIA MARCONA 69	20129	MILANO	PRIVATE
175	030	LOMBARDIA	30101	CASA DI CURA VILLA LETIZIA - MILANO	VIA G.DONIZETTI, 10/12	20122	MILANO	PRIVATE
176	030	LOMBARDIA	30103	CASA DI CURA PALAZZOLO-FOND.DON GNOCCHI	VIA DON L.PALAZZOLO 21	20149	MILANO	PRIVATE
177	030	LOMBARDIA	30104	CASA DI CURA S.RAFFAELE TURRO	VIA STAMINA D'ANCONA, 20	20127	MILANO	PRIVATE
178	030	LOMBARDIA	30105	CASA DI CURA S. CAMILLO - MILANO	VIA MAURO MACCHI N. 5	20124	MILANO	PRIVATE
179	030	LOMBARDIA	30110	CASA DI CURA S. GIOVANNI - MILANO	VIA CIVITALI 71	20148	MILANO	PRIVATE
180	030	LOMBARDIA	30112	IST.CLINICO S.AMBROGIO SPA-MILANO	VIA FARAVELLI 16	20149	MILANO	PRIVATE
181	030	LOMBARDIA	30113	CASA DI CURA LA MADONNINA - MILANO	VIA QUADRONNO 29	20122	MILANO	PRIVATE
182	030	LOMBARDIA	30118	EUKOS SPA/CASA DI CURA S.CARLO MI	VIA PIER LOMBARDO, 22	20135	MILANO	PRIVATE
183	030	LOMBARDIA	30119	IST.CLINICO S.SIRO SPA-MILANO	VIA MONREALE 18	20148	MILANO	PRIVATE
184	030	LOMBARDIA	30121	CASA DI CURA S. PIO X - MILANO	VIA FRANCESCO NAVA 31	20159	MILANO	PRIVATE
185	030	LOMBARDIA	30124	ISTITUTO STOMATOLOGICO ITALIANO - MILANO	VIA PACE 21	20122	MILANO	PRIVATE
186	030	LOMBARDIA	30266	CASA DI CURA COLUMBUS - MILANO	VIA BUONARROTI 48	20145	MILANO	PRIVATE
187	030	LOMBARDIA	30269	CASA DI CURA CAPITANIO - MILANO	VIA MERCALLI 30	20122	MILANO	PRIVATE
188	030	LOMBARDIA	30367	OSPEDALE S. GIUSEPPE - MILANO	VIA S. VITTORE 12	15146	MILANO	PUBLIC
189	030	LOMBARDIA	30109	MULTIMEDICA HOLDING SPA - LIMBIATE	VIA F.LLI BANDIERA 3	20051	LIMBIATE	PRIVATE
190	030	LOMBARDIA	30116	CLINICA SAN CARLO - PADERNO DUGNANO	VIA OSPEDALE 21	20037	PADERNO DUGNANO	PRIVATE
191	030	LOMBARDIA	30125	CASA DI CURA AMBROSIANA SPA-CESANO B.	PIAZZA MONS. MONETA 1	20090	CESANO BOSCONE	PRIVATE
192	030	LOMBARDIA	30359	FONDAZIONE EUROPEA DI RICERCA BIOMEDICA	VIA UBOLDO, 19	20063	CERNUSCO SUL NAVIGLIO	PRIVATE
193	030	LOMBARDIA	30363	RESIDENZA ANNI AZZURRI MIRASOLE	VIA BORSELLINO, 6 - LOC. NOVERASCO	20090	OPERA	PRIVATE
194	030	LOMBARDIA	30106	POLICLINICO DI MONZA - CASA DI CURA PRIV	VIA AMATI N.111	20052	MONZA	PRIVATE
195	030	LOMBARDIA	30107	ISTITUTI CLINICI ZUCCHI SPA-MONZA	VIA ZUCCHI 24	20052	MONZA	PRIVATE
196	030	LOMBARDIA	30108	CASA DI CURA ZUCCHI - CARATE BRIANZA	PIAZZA MADONNINA N.1	20048	CARATE BRIANZA	PRIVATE
197	030	LOMBARDIA	30352	POLO GERIATRICO RIABILITATIVO-CINISELLO	VIA DEI LAVORATORI, 133	20092	CINISELLO BALSAMO	PRIVATE
198	030	LOMBARDIA	30196	IST.CLIN.PROF.E.MORELLI	PIAZZA XXIV MAGGIO 13	27100	PAVIA	PRIVATE
199	030	LOMBARDIA	30197	CASA DI CURA VILLA ESPERIA	V.LE DEI SALICI, 35	27056	GODIASCO	PRIVATE
200	030	LOMBARDIA	30198	CASA DI CURA BEATO MATTEO	CSO PAVIA 84	27029	VIGEVANO	PRIVATE
201	030	LOMBARDIA	30199	CASA DI CURA S. MARIA GRAZIE	CSO F.LLI ROSSELLI 59	27058	VOGHERA	PRIVATE
202	030	LOMBARDIA	30201	CASA DI CURA CITTADELLA SOCIALE	VIA AVANZA 8	27037	PIEVE DEL CAIRO	PRIVATE
203	030	LOMBARDIA	30202	IST.DI CURA CITTA'DI PAVIA	VIALE PARCO VECCHIO 27	27100	PAVIA	PRIVATE
204	030	LOMBARDIA	30014	CASA DI CURA LA QUIETE - VARESE	VIA DANTE 20	21100	VARESE	PRIVATE
205	030	LOMBARDIA	30015	CASA DI CURA SANTA MARIA - CASTELLANZA	VIALE PIEMONTE, 70	21053	CASTELLANZA	PRIVATE
206	030	LOMBARDIA	30016	CASA DI CURA VILLA PREZIOSA - LAVENO	VIA BELLORINI, 48	21014	LAVENO-MOMBELLO	PRIVATE
207	030	LOMBARDIA	30017	CASA DI CURA MATER DOMINI - CASTELLANZA	VIA GERENZANO 2	21053	CASTELLANZA	PRIVATE
208	030	LOMBARDIA	30324	CASA DI CURA LE TERRAZZE - CUNARDO	VIA FOSCOLO, 6/B	21035	CUNARDO	PRIVATE
209	030	LOMBARDIA	30364	"LA FONDAZIONE" DI A.BORGHI & C.-BREBBIA	VIA PETRARCA, 33	21026	BREBBIA	PRIVATE
210	030	LOMBARDIA	30365	FONDAZ.PAOLO E TITO F.LLI MOLINA-ONLUS	V.LE LUIGI BORRI, 133	21100	VARESE	PRIVATE
211	030	LOMBARDIA	30371	CENTRO DIAG. TERAP.FOND. BORGHI	VIA PETRARCA, 33	21020	BREBBIA	PRIVATE
212	030	LOMBARDIA	30274	OSPEDALE VALCAMONICA - ESINE	VIA MANZONI	25040	ESINE	PUBLIC
213	030	LOMBARDIA	30274	OSPEDALE VALCAMONICA - ESINE	VIA MANZONI	25040	ESINE	PUBLIC
214	030	LOMBARDIA	30920	C.TRO BRONCOPNEUMOPATIE INRCA-CASATENOVO	VIA MONTEREGIO, 13	23880	CASATENOVO	PUBLIC
215	030	LOMBARDIA	30922	FOND.IRCCS "ISTIT.NAZ.LE TUMORI"MILANO	VIA VENEZIAN, 1	20133	MILANO	PUBLIC
216	030	LOMBARDIA	30923	FOND.IRCCS IST.NAZ.NEUROLOGICO C.BESTA	VIA CELORIA, 11	20133	MILANO	PUBLIC
217	030	LOMBARDIA	30924	OSPEDALE POLICLINICO S. MATTEO - PAVIA	P.LE GOLGI, 19	27100	PAVIA	PUBLIC
218	030	LOMBARDIA	30925	FONDAZ.POLICLINICO,MANGIAGALLI,REG.ELENA	VIA FRANCESCO SFORZA, 28	20122	MILANO	PUBLIC
219	030	LOMBARDIA	30925	FONDAZ.POLICLINICO,MANGIAGALLI,REG.ELENA	VIA FRANCESCO SFORZA, 28	20122	MILANO	PUBLIC
220	030	LOMBARDIA	30930	FOND.S.MAUGERI-CL.LAVORO E RIAB.-TRADATE	VIA RONCACCIO, 16	21049	TRADATE	PUBLIC
221	030	LOMBARDIA	30931	ISTITUTO SCIENTIFICO MEDEA-BOSISIO P.	VIA DON L. MONZA, 20	23842	BOSISIO PARINI	PUBLIC
222	030	LOMBARDIA	30932	FOND.S.MAUGERI-CL.LAV.E RIAB.-LUMEZZANE	VIA MAZZINI, 129	25066	LUMEZZANE	PUBLIC
223	030	LOMBARDIA	30933	FOND.S.MAUGERI-CENTRO MEDICO-CASTEL G.	VIA OSPEDALE, 36	46042	CASTEL GOFFREDO	PUBLIC
224	030	LOMBARDIA	30934	CENTRO CARDIOLOGICO SPA "FOND. MONZINO"	VIA CARLO PAREA, 4	20138	MILANO	PUBLIC
225	030	LOMBARDIA	30935	IRCCS S. RAFFAELE - MILANO	VIA OLGETTINA, 60	20132	MILANO	PUBLIC
226	030	LOMBARDIA	30936	IST.AUXOLOGICO ITALIANO-I.S. S.LUCA-MI	VIA LUDOVICO ARIOSTO, 13	20145	MILANO	PUBLIC
227	030	LOMBARDIA	30936	IST.AUXOLOGICO ITALIANO-I.S. S.LUCA-MI	VIA LUDOVICO ARIOSTO, 13	20145	MILANO	PUBLIC
228	030	LOMBARDIA	30937	IRCCS S.M.NASCENTE-FOND.DON C.GNOCCHI	VIA CAPECELATRO, 66	20148	MILANO	PUBLIC
229	030	LOMBARDIA	30938	FOND.MAUGERI-CENTRO MEDICO DI PAVIA	VIA SALVATORE MAUGERI, 10/10A	27100	PAVIA	PUBLIC
230	030	LOMBARDIA	30939	FOND.IST.NEUROL.C.MONDINO-PAVIA	VIA MONDINO, 2	27100	PAVIA	PUBLIC
231	030	LOMBARDIA	30940	MAUGERI-CENTRO MEDICO MONTESCANO	VIA PER MONTESCANO 31	27040	MONTESCANO	PUBLIC

232	030	LOMBARDIA	30941	ISTITUTO EUROPEO DI ONCOLOGIA-MILANO	VIA RIPAMONTI, 435	20141	MILANO	PUBLIC
233	030	LOMBARDIA	30942	IRCCS S.GIOVANNI DI DIO-FBF- BRESCIA	VIA PILASTRONI, 4	25125	BRESCIA	PUBLIC
234	030	LOMBARDIA	30943	IST. CLIN. HUMANITAS - ROZZANO	VIA MANZONI, 56	20089	ROZZANO	PUBLIC
235	030	LOMBARDIA	30944	FONDAZIONE S. MAUGERI - LISSONE	VIA BERNASCONI, 16	20035	LISSONE	PUBLIC
236	030	LOMBARDIA	30945	ISTITUTO SCIENTIFICO DI PAVIA	VIA BOEZIO 26	27100	PAVIA	PUBLIC
237	030	LOMBARDIA	30946	ISTITUTO ORTOPEDICO GALEAZZI SPA	VIA RICCARDO GALEAZZI, 4	20161	MILANO	PUBLIC
238	030	LOMBARDIA	30947	I.R.C.C.S. POLICLINICO SAN DONATO	VIA MORANDI, 30	20097	SAN DONATO MILANESE	PUBLIC
239	030	LOMBARDIA	30948	MULTIMEDICA HOLDING SPA-SESTO S.G.	VIA MILANESE, 300	20099	SESTO SAN GIOVANNI	PUBLIC
240	030	LOMBARDIA	30951	A.O. OSP.CIRC. FONDAZIONE MACCHI-VARESE	VIALE LUIGI BORRI, 57	21100	VARESE	PUBLIC
241	030	LOMBARDIA	30951	A.O. OSP.CIRC. FONDAZIONE MACCHI-VARESE	VIALE LUIGI BORRI, 57	21100	VARESE	PUBLIC
242	030	LOMBARDIA	30951	A.O. OSP.CIRC. FONDAZIONE MACCHI-VARESE	VIALE LUIGI BORRI, 57	21100	VARESE	PUBLIC
243	030	LOMBARDIA	30951	A.O. OSP.CIRC. FONDAZIONE MACCHI-VARESE	VIALE LUIGI BORRI, 57	21100	VARESE	PUBLIC
244	030	LOMBARDIA	30951	A.O. OSP.CIRC. FONDAZIONE MACCHI-VARESE	VIALE LUIGI BORRI, 57	21100	VARESE	PUBLIC
245	030	LOMBARDIA	30952	A.O. S.ANTONIO ABATE - GALLARATE	L.GO BOITO 2	21013	GALLARATE	PUBLIC
246	030	LOMBARDIA	30952	A.O. S.ANTONIO ABATE - GALLARATE	L.GO BOITO 2	21013	GALLARATE	PUBLIC
247	030	LOMBARDIA	30952	A.O. S.ANTONIO ABATE - GALLARATE	L.GO BOITO 2	21013	GALLARATE	PUBLIC
248	030	LOMBARDIA	30953	A.O. OSPEDALE DI CIRCOLO - BUSTO ARSIZIO	PIAZZALE SOLARO, 3	21052	BUSTO ARSIZIO	PUBLIC
249	030	LOMBARDIA	30953	A.O. OSPEDALE DI CIRCOLO - BUSTO ARSIZIO	PIAZZALE SOLARO, 3	21052	BUSTO ARSIZIO	PUBLIC
250	030	LOMBARDIA	30953	A.O. OSPEDALE DI CIRCOLO - BUSTO ARSIZIO	PIAZZALE SOLARO, 3	21052	BUSTO ARSIZIO	PUBLIC
251	030	LOMBARDIA	30954	A.O. SPEDALI CIVILI - BRESCIA	PIAZZALE SPEDALI CIVILI, 1	25125	BRESCIA	PUBLIC
252	030	LOMBARDIA	30954	A.O. SPEDALI CIVILI - BRESCIA	PIAZZALE SPEDALI CIVILI, 1	25125	BRESCIA	PUBLIC
253	030	LOMBARDIA	30954	A.O. SPEDALI CIVILI - BRESCIA	PIAZZALE SPEDALI CIVILI, 1	25125	BRESCIA	PUBLIC
254	030	LOMBARDIA	30954	A.O. SPEDALI CIVILI - BRESCIA	PIAZZALE SPEDALI CIVILI, 1	25125	BRESCIA	PUBLIC
255	030	LOMBARDIA	30954	A.O. SPEDALI CIVILI - BRESCIA	PIAZZALE SPEDALI CIVILI, 1	25125	BRESCIA	PUBLIC
256	030	LOMBARDIA	30955	A.O. "MELLINO MELLINI" - CHIARI	VIALE MAZZINI, 4	25032	CHIARI	PUBLIC
257	030	LOMBARDIA	30955	A.O. "MELLINO MELLINI" - CHIARI	VIALE MAZZINI, 4	25032	CHIARI	PUBLIC
258	030	LOMBARDIA	30955	A.O. "MELLINO MELLINI" - CHIARI	VIALE MAZZINI, 4	25032	CHIARI	PUBLIC
259	030	LOMBARDIA	30955	A.O. "MELLINO MELLINI" - CHIARI	VIALE MAZZINI, 4	25032	CHIARI	PUBLIC
260	030	LOMBARDIA	30955	A.O. "MELLINO MELLINI" - CHIARI	VIALE MAZZINI, 4	25032	CHIARI	PUBLIC
261	030	LOMBARDIA	30956	A.O. DESENZANO DEL GARDA - DESENZANO G.	LOCALITA' MONTECROCE	25015	DESENZANO DEL GARDA	PUBLIC
262	030	LOMBARDIA	30956	A.O. DESENZANO DEL GARDA - DESENZANO G.	LOCALITA' MONTECROCE	25015	DESENZANO DEL GARDA	PUBLIC
263	030	LOMBARDIA	30956	A.O. DESENZANO DEL GARDA - DESENZANO G.	LOCALITA' MONTECROCE	25015	DESENZANO DEL GARDA	PUBLIC
264	030	LOMBARDIA	30956	A.O. DESENZANO DEL GARDA - DESENZANO G.	LOCALITA' MONTECROCE	25015	DESENZANO DEL GARDA	PUBLIC
265	030	LOMBARDIA	30956	A.O. DESENZANO DEL GARDA - DESENZANO G.	LOCALITA' MONTECROCE	25015	DESENZANO DEL GARDA	PUBLIC
266	030	LOMBARDIA	30956	A.O. DESENZANO DEL GARDA - DESENZANO G.	LOCALITA' MONTECROCE	25015	DESENZANO DEL GARDA	PUBLIC
267	030	LOMBARDIA	30957	A.O. ISTITUTI OSPEDALIERI - CREMONA	LARGO PRIORI, 1	26100	CREMONA	PUBLIC
268	030	LOMBARDIA	30957	A.O. ISTITUTI OSPEDALIERI - CREMONA	LARGO PRIORI, 1	26100	CREMONA	PUBLIC
269	030	LOMBARDIA	30958	A.O. OSPEDALE MAGGIORE - CREMA	VIA GRAMSCI 13	26013	CREMA	PUBLIC
270	030	LOMBARDIA	30958	A.O. OSPEDALE MAGGIORE - CREMA	VIA GRAMSCI 13	26013	CREMA	PUBLIC
271	030	LOMBARDIA	30958	A.O. OSPEDALE MAGGIORE - CREMA	VIA GRAMSCI 13	26013	CREMA	PUBLIC
272	030	LOMBARDIA	30959	A.O. S.ANNA - COMO	VIA NAPOLEONA, 60	22100	COMO	PUBLIC
273	030	LOMBARDIA	30959	A.O. S.ANNA - COMO	VIA NAPOLEONA, 60	22100	COMO	PUBLIC
274	030	LOMBARDIA	30959	A.O. S.ANNA - COMO	VIA NAPOLEONA, 60	22100	COMO	PUBLIC
275	030	LOMBARDIA	30959	A.O. S.ANNA - COMO	VIA NAPOLEONA, 60	22100	COMO	PUBLIC
276	030	LOMBARDIA	30959	A.O. S.ANNA - COMO	VIA NAPOLEONA, 60	22100	COMO	PUBLIC
277	030	LOMBARDIA	30960	A.O. OSPEDALE DI LECCO - LECCO	VIA DELL'EREMO, 9/11	23900	LECCO	PUBLIC
278	030	LOMBARDIA	30960	A.O. OSPEDALE DI LECCO - LECCO	VIA DELL'EREMO, 9/11	23900	LECCO	PUBLIC
279	030	LOMBARDIA	30960	A.O. OSPEDALE DI LECCO - LECCO	VIA DELL'EREMO, 9/11	23900	LECCO	PUBLIC
280	030	LOMBARDIA	30962	A.O. OSPEDALI RIUNITI - BERGAMO	LARGO BAROZZI, 1	24128	BERGAMO	PUBLIC
281	030	LOMBARDIA	30963	A.O. OSP.TREVIGLIO CARAVAGGIO-TREVIGLIO	PIAZZA OSPEDALE 1	24047	TREVIGLIO	PUBLIC
282	030	LOMBARDIA	30963	A.O. OSP.TREVIGLIO CARAVAGGIO-TREVIGLIO	PIAZZA OSPEDALE 1	24047	TREVIGLIO	PUBLIC
283	030	LOMBARDIA	30963	A.O. OSP.TREVIGLIO CARAVAGGIO-TREVIGLIO	PIAZZA OSPEDALE 1	24047	TREVIGLIO	PUBLIC
284	030	LOMBARDIA	30963	A.O. OSP.TREVIGLIO CARAVAGGIO-TREVIGLIO	PIAZZA OSPEDALE 1	24047	TREVIGLIO	PUBLIC
285	030	LOMBARDIA	30964	A.O. "BOLOGNINI" - SERIATE	VIA MARCONI, 38	24068	SERIATE	PUBLIC
286	030	LOMBARDIA	30964	A.O. "BOLOGNINI" - SERIATE	VIA MARCONI, 38	24068	SERIATE	PUBLIC
287	030	LOMBARDIA	30964	A.O. "BOLOGNINI" - SERIATE	VIA MARCONI, 38	24068	SERIATE	PUBLIC
288	030	LOMBARDIA	30964	A.O. "BOLOGNINI" - SERIATE	VIA MARCONI, 38	24068	SERIATE	PUBLIC
289	030	LOMBARDIA	30964	A.O. "BOLOGNINI" - SERIATE	VIA MARCONI, 38	24068	SERIATE	PUBLIC
290	030	LOMBARDIA	30965	A.O. "CARLO POMA" - MANTOVA	VIA ALBERTONI, 1	46100	MANTOVA	PUBLIC
291	030	LOMBARDIA	30965	A.O. "CARLO POMA" - MANTOVA	VIA ALBERTONI, 1	46100	MANTOVA	PUBLIC
292	030	LOMBARDIA	30965	A.O. "CARLO POMA" - MANTOVA	VIA ALBERTONI, 1	46100	MANTOVA	PUBLIC
293	030	LOMBARDIA	30965	A.O. "CARLO POMA" - MANTOVA	VIA ALBERTONI, 1	46100	MANTOVA	PUBLIC
294	030	LOMBARDIA	30966	A.O. "LUIGI SACCO" - MILANO	VIA S. GIOVANNI BATTISTA GRASSI, 74	20157	MILANO	PUBLIC

295	030	LOMBARDIA	30967	A.O. "OSP.NIGUARDA CA'GRANDA"-MILANO	PIAZZA OSP. MAGGIORE,3	20162	MILANO	PUBLIC
296	030	LOMBARDIA	30968	A.O. "IST.CLIN.PERFEZIONAMENTO"-MILANO	VIA DAVERIO, 6	20122	MILANO	PUBLIC
297	030	LOMBARDIA	30968	A.O. "IST.CLIN.PERFEZIONAMENTO"-MILANO	VIA DAVERIO, 6	20122	MILANO	PUBLIC
298	030	LOMBARDIA	30969	A.O. FATEBENEFRATELLI E OFTALMICO-MILANO	CORSO DI PORTA NUOVA, 23	20121	MILANO	PUBLIC
299	030	LOMBARDIA	30969	A.O. FATEBENEFRATELLI E OFTALMICO-MILANO	CORSO DI PORTA NUOVA, 23	20121	MILANO	PUBLIC
300	030	LOMBARDIA	30970	A.O. "SAN PAOLO" - MILANO	VIA A. DI RUDINI', 8	20142	MILANO	PUBLIC
301	030	LOMBARDIA	30971	A.O. IST.ORTOPEDICO GAETANO PINI-MILANO	PIAZZA CARDINAL FERRARI, 1	20122	MILANO	PUBLIC
302	030	LOMBARDIA	30972	A.O. "S.CARLO BORROMEO" - MILANO	VIA PIO II, 3	20153	MILANO	PUBLIC
303	030	LOMBARDIA	30973	A.O. OSPEDALE CIVILE - LEGNANO	CORSO SEMPIONE, 82	20025	LEGNANO	PUBLIC
304	030	LOMBARDIA	30973	A.O. OSPEDALE CIVILE - LEGNANO	CORSO SEMPIONE, 82	20025	LEGNANO	PUBLIC
305	030	LOMBARDIA	30973	A.O. OSPEDALE CIVILE - LEGNANO	CORSO SEMPIONE, 82	20025	LEGNANO	PUBLIC
306	030	LOMBARDIA	30973	A.O. OSPEDALE CIVILE - LEGNANO	CORSO SEMPIONE, 82	20025	LEGNANO	PUBLIC
307	030	LOMBARDIA	30263	PRES.RIABILITATIVO NPI CORBERI-LIMBIATE	VIA MONTE GRAPPA 19	20051	LIMBIATE	PUBLIC
308	030	LOMBARDIA	30974	A.O. "G. SALVINI" - GARBAGNATE	VIALE FORLANINI, 121	20024	GARBAGNATE MILANESE	PUBLIC
309	030	LOMBARDIA	30974	A.O. "G. SALVINI" - GARBAGNATE	VIALE FORLANINI, 121	20024	GARBAGNATE MILANESE	PUBLIC
310	030	LOMBARDIA	30974	A.O. "G. SALVINI" - GARBAGNATE	VIALE FORLANINI, 121	20024	GARBAGNATE MILANESE	PUBLIC
311	030	LOMBARDIA	30974	A.O. "G. SALVINI" - GARBAGNATE	VIALE FORLANINI, 121	20024	GARBAGNATE MILANESE	PUBLIC
312	030	LOMBARDIA	30975	A.O. OSPEDALE DI CIRCOLO - MELEGNANO	VIA PANDINA, 1	20070	VIZZOLO PREDABISSI	PUBLIC
313	030	LOMBARDIA	30975	A.O. OSPEDALE DI CIRCOLO - MELEGNANO	VIA PANDINA, 1	20070	VIZZOLO PREDABISSI	PUBLIC
314	030	LOMBARDIA	30975	A.O. OSPEDALE DI CIRCOLO - MELEGNANO	VIA PANDINA, 1	20070	VIZZOLO PREDABISSI	PUBLIC
315	030	LOMBARDIA	30975	A.O. OSPEDALE DI CIRCOLO - MELEGNANO	VIA PANDINA, 1	20070	VIZZOLO PREDABISSI	PUBLIC
316	030	LOMBARDIA	30975	A.O. OSPEDALE DI CIRCOLO - MELEGNANO	VIA PANDINA, 1	20070	VIZZOLO PREDABISSI	PUBLIC
317	030	LOMBARDIA	30976	A.O. OSPEDALE CIVILE - VIMERCATE	VIA C. BATTISTI,23	20059	VIMERCATE	PUBLIC
318	030	LOMBARDIA	30976	A.O. OSPEDALE CIVILE - VIMERCATE	VIA C. BATTISTI,23	20059	VIMERCATE	PUBLIC
319	030	LOMBARDIA	30976	A.O. OSPEDALE CIVILE - VIMERCATE	VIA C. BATTISTI,23	20059	VIMERCATE	PUBLIC
320	030	LOMBARDIA	30976	A.O. OSPEDALE CIVILE - VIMERCATE	VIA C. BATTISTI,23	20059	VIMERCATE	PUBLIC
321	030	LOMBARDIA	30976	A.O. OSPEDALE CIVILE - VIMERCATE	VIA C. BATTISTI,23	20059	VIMERCATE	PUBLIC
322	030	LOMBARDIA	30976	A.O. OSPEDALE CIVILE - VIMERCATE	VIA C. BATTISTI,23	20059	VIMERCATE	PUBLIC
323	030	LOMBARDIA	30976	A.O. OSPEDALE CIVILE - VIMERCATE	VIA C. BATTISTI,23	20059	VIMERCATE	PUBLIC
324	030	LOMBARDIA	30977	A.O. "S.GERARDO" - MONZA	VIA SOLFERINO, 16	20052	MONZA	PUBLIC
325	030	LOMBARDIA	30977	A.O. "S.GERARDO" - MONZA	VIA SOLFERINO, 16	20052	MONZA	PUBLIC
326	030	LOMBARDIA	30978	A.O. DELLA PROVINCIA DI LODI	PIAZZA OSPEDALE, 10	26900	LODI	PUBLIC
327	030	LOMBARDIA	30978	A.O. DELLA PROVINCIA DI LODI	PIAZZA OSPEDALE, 10	26900	LODI	PUBLIC
328	030	LOMBARDIA	30978	A.O. DELLA PROVINCIA DI LODI	PIAZZA OSPEDALE, 10	26900	LODI	PUBLIC
329	030	LOMBARDIA	30978	A.O. DELLA PROVINCIA DI LODI	PIAZZA OSPEDALE, 10	26900	LODI	PUBLIC
330	030	LOMBARDIA	30979	A.O. DELLA PROVINCIA DI PAVIA	VIALE REPUBBLICA, 34	27100	PAVIA	PUBLIC
331	030	LOMBARDIA	30979	A.O. DELLA PROVINCIA DI PAVIA	VIALE REPUBBLICA, 34	27100	PAVIA	PUBLIC
332	030	LOMBARDIA	30979	A.O. DELLA PROVINCIA DI PAVIA	VIALE REPUBBLICA, 34	27100	PAVIA	PUBLIC
333	030	LOMBARDIA	30979	A.O. DELLA PROVINCIA DI PAVIA	VIALE REPUBBLICA, 34	27100	PAVIA	PUBLIC
334	030	LOMBARDIA	30979	A.O. DELLA PROVINCIA DI PAVIA	VIALE REPUBBLICA, 34	27100	PAVIA	PUBLIC
335	030	LOMBARDIA	30979	A.O. DELLA PROVINCIA DI PAVIA	VIALE REPUBBLICA, 34	27100	PAVIA	PUBLIC
336	030	LOMBARDIA	30979	A.O. DELLA PROVINCIA DI PAVIA	VIALE REPUBBLICA, 34	27100	PAVIA	PUBLIC
337	030	LOMBARDIA	30979	A.O. DELLA PROVINCIA DI PAVIA	VIALE REPUBBLICA, 34	27100	PAVIA	PUBLIC
338	030	LOMBARDIA	30980	A.O. VALTELLINA E VALCHIAVENNA	VIA STELVIO, 25	23100	SONDRIO	PUBLIC
339	030	LOMBARDIA	30980	A.O. VALTELLINA E VALCHIAVENNA	VIA STELVIO, 25	23100	SONDRIO	PUBLIC
340	030	LOMBARDIA	30980	A.O. VALTELLINA E VALCHIAVENNA	VIA STELVIO, 25	23100	SONDRIO	PUBLIC
341	030	LOMBARDIA	30980	A.O. VALTELLINA E VALCHIAVENNA	VIA STELVIO, 25	23100	SONDRIO	PUBLIC
342	041	RENTINO-ALTO ADIGI	41001	OSPEDALE CENTRALE DI BOLZANO	VIA L. BOEHLER, 5	39100	BOLZANO	PUBLIC
343	041	RENTINO-ALTO ADIGI	41002	OSPEDALE AZIENDALE DI MERANO	via Rossini, 5	39012	MERANO	PUBLIC
344	041	RENTINO-ALTO ADIGI	41004	OSPEDALE AZIENDALE DI BRESSANONE	Via Dante 51	39042	BRESSANONE	PUBLIC
345	041	RENTINO-ALTO ADIGI	41005	OSPEDALE AZIENDALE DI BRUNICO	Via ospedale 11	39031	BRUNICO	PUBLIC
346	041	RENTINO-ALTO ADIGI	41006	OSPEDALE DI BASE DI VIPITENO	Via S.Margherita 24	39049	VIPITENO	PUBLIC
347	041	RENTINO-ALTO ADIGI	41007	OSPEDALE DI BASE DI SAN CANDIDO	Via Freising 2	39038	SAN CANDIDO	PUBLIC
348	041	RENTINO-ALTO ADIGI	41011	OSPEDALE DI BASE DI SILANDRO	via Ospedale, 3	39028	SILANDRO	PUBLIC
349	041	RENTINO-ALTO ADIGI	41012	FONDAZIONE SARENTINO	VICOLO OSPEDALE, 3/5	39058	SARENTINO	PRIVATE
350	041	RENTINO-ALTO ADIGI	41013	CASA DI CURA SANTA MARIA	VIA CLAUDIA DE MEDICI, 2	39100	BOLZANO	PRIVATE
351	041	RENTINO-ALTO ADIGI	41014	CASA DI CURA VILLA SANT'ANNA	Via Cavour, 58	39012	MERANO	PRIVATE
352	041	RENTINO-ALTO ADIGI	41015	CASA DI CURA BONVICINI S.R.L.	VIA MICHAEL PACHER, 12	39100	BOLZANO	PRIVATE
353	041	RENTINO-ALTO ADIGI	41017	CASA DI CURA VILLA MELITTA	VIA COL DI LANA, 6	39100	BOLZANO	PRIVATE
354	041	RENTINO-ALTO ADIGI	41018	CASA DI CURA FONTE SAN MARTINO	Via Laurin, 70	39012	MERANO	PRIVATE
355	042	RENTINO-ALTO ADIGI	42001	OSPEDALE DI TRENTO	LARGO MEDAGLIE D ORO, 1	38100	TRENTO	PUBLIC
356	042	RENTINO-ALTO ADIGI	42001	OSPEDALE DI TRENTO	LARGO MEDAGLIE D ORO, 1	38100	TRENTO	PUBLIC
357	042	RENTINO-ALTO ADIGI	42001	OSPEDALE DI TRENTO	LARGO MEDAGLIE D ORO, 1	38100	TRENTO	PUBLIC

358	042	TRENTINO-ALTO ADIGI	42004	PRESIDIO OSPEDALIERO S.LORENZO	VIALE VICENZA 9	38051	BORGO VALSUGANA	PUBLIC
359	042	TRENTINO-ALTO ADIGI	42005	PRESIDIO OSPEDALIERO DI CLES	VIALE DEGASPERI, 31	38023	CLES	PUBLIC
360	042	TRENTINO-ALTO ADIGI	42006	OSPEDALE DI ROVERETO	CORSO VERONA, 4	38068	ROVERETO	PUBLIC
361	042	TRENTINO-ALTO ADIGI	42006	OSPEDALE DI ROVERETO	CORSO VERONA, 4	38068	ROVERETO	PUBLIC
362	042	TRENTINO-ALTO ADIGI	42007	PRESIDIO OSPEDALIERO DI TIONE	VIA OSPEDALE, 11	38079	TIONE DI TRENTO	PUBLIC
363	042	TRENTINO-ALTO ADIGI	42010	PRESIDIO OSPEDALIERO DI ARCO	VIA CAPITELLI, 48	38062	ARCO	PUBLIC
364	042	TRENTINO-ALTO ADIGI	42014	PRESIDIO OSPEDALIERO DI CAVALESE	VIA DOSSI 17	38033	CAVALESE	PUBLIC
365	042	TRENTINO-ALTO ADIGI	42015	OSPEDALE SAN CAMILLO	VIA PODESTA GIOVANELLI 19	38100	TRENTO	PUBLIC
366	042	TRENTINO-ALTO ADIGI	42016	OSPEDALE SAN PANCRAZIO	VIA D.CHIESA 2	38062	ARCO	PUBLIC
367	042	TRENTINO-ALTO ADIGI	42018	CASA DI CURA EREMO DI ARCO SRL	VIA XXI APRILE, 1	38062	ARCO	PRIVATE
368	042	TRENTINO-ALTO ADIGI	42019	CASA DI CURA REGINA	VIA POMERIO, 3	38062	ARCO	PRIVATE
369	042	TRENTINO-ALTO ADIGI	42020	CASA DI CURA SOLATRIX	VIA BELLINI 11	38068	ROVERETO	PRIVATE
370	042	TRENTINO-ALTO ADIGI	42021	CASA DI CURA SACRA FAMIGLIA	VIA STRAPPAZOCCHE, 1	38062	ARCO	PRIVATE
371	042	TRENTINO-ALTO ADIGI	42022	CASA DI CURA VILLA BIANCA	VIA PIAVE 78	38100	TRENTO	PRIVATE
372	050	VENETO	50059	ISTITUTO CODIVILLA - PUTTI CORTINA	VIA CODIVILLA 1	32043	CORTINA D`AMPEZZO	PUBLIC
373	050	VENETO	50201	OSPEDALE DELL`ULSS N. 1 BELLUNO	VIALE EUROPA	32100	BELLUNO	PUBLIC
374	050	VENETO	50201	OSPEDALE DELL`ULSS N. 1 BELLUNO	VIALE EUROPA	32100	BELLUNO	PUBLIC
375	050	VENETO	50201	OSPEDALE DELL`ULSS N. 1 BELLUNO	VIALE EUROPA	32100	BELLUNO	PUBLIC
376	050	VENETO	50202	OSPEDALE DI FELTRE	VIA BAGNOLS SUR CEZE, 3	32032	FELTRE	PUBLIC
377	050	VENETO	50202	OSPEDALE DI FELTRE	VIA BAGNOLS SUR CEZE, 3	32032	FELTRE	PUBLIC
378	050	VENETO	50203	OSPEDALE DELL`AZIENDA ULSS N. 3	VIA DEI LOTTI, 40	36061	BASSANO DEL GRAPPA	PUBLIC
379	050	VENETO	50203	OSPEDALE DELL`AZIENDA ULSS N. 3	VIA DEI LOTTI, 40	36061	BASSANO DEL GRAPPA	PUBLIC
380	050	VENETO	50204	OSPEDALE ULSS 4	VIA RASA, 9	36016	THIENE	PUBLIC
381	050	VENETO	50204	OSPEDALE ULSS 4	VIA RASA, 9	36016	THIENE	PUBLIC
382	050	VENETO	50205	OSPEDALI AZIENDA ULSS 5 ARZIGNANO	VIA TRENTO, 4	36071	ARZIGNANO	PUBLIC
383	050	VENETO	50205	OSPEDALI AZIENDA ULSS 5 ARZIGNANO	VIA TRENTO, 4	36071	ARZIGNANO	PUBLIC
384	050	VENETO	50205	OSPEDALI AZIENDA ULSS 5 ARZIGNANO	VIA TRENTO, 4	36071	ARZIGNANO	PUBLIC
385	050	VENETO	50205	OSPEDALI AZIENDA ULSS 5 ARZIGNANO	VIA TRENTO, 4	36071	ARZIGNANO	PUBLIC
386	050	VENETO	50045	CASA DI CURA ERETENIA	VIALE ERETENIO, 12	36100	VICENZA	PRIVATE
387	050	VENETO	50046	CASA DI CURA VILLA BERICA	VIA CAPPAROZZO, 10	36100	VICENZA	PRIVATE
388	050	VENETO	50047	CASA DI CURA VILLA MARGHERITA	VIA COSTACOLONNA, 6	36057	ARCUGNANO	PRIVATE
389	050	VENETO	50206	ULSS.6 VICENZA	VIALE F. RODOLFI, 37	36100	VICENZA	PUBLIC
390	050	VENETO	50206	ULSS.6 VICENZA	VIALE F. RODOLFI, 37	36100	VICENZA	PUBLIC
391	050	VENETO	50140	ASSOCIAZIONE LA NS. FAMIGLIA IRCSS MEDEA	VIA COSTA ALTA, 37	31015	CONEGLIANO	PUBLIC
392	050	VENETO	50140	ASSOCIAZIONE LA NS. FAMIGLIA IRCSS MEDEA	VIA COSTA ALTA, 37	31015	CONEGLIANO	PUBLIC
393	050	VENETO	50207	OSPEDALE DI ULSS 7	VIA BRIGATA BISAGNO, 4	31015	CONEGLIANO	PUBLIC
394	050	VENETO	50207	OSPEDALE DI ULSS 7	VIA BRIGATA BISAGNO, 4	31015	CONEGLIANO	PUBLIC
395	050	VENETO	50207	OSPEDALE DI ULSS 7	VIA BRIGATA BISAGNO, 4	31015	CONEGLIANO	PUBLIC
396	050	VENETO	50208	AZIENDA ULSS N.8 - ASOLO	VIA FORESTUZZO, 41	31011	ASOLO	PUBLIC
397	050	VENETO	50208	AZIENDA ULSS N.8 - ASOLO	VIA FORESTUZZO, 41	31011	ASOLO	PUBLIC
398	050	VENETO	50072	OSPEDALE RIABILITATIVO DI ALTA SPECIAL.	VIA P. BELLO , 3/C	31045	MOTTA DI LIVENZA	PUBLIC
399	050	VENETO	50079	C.C S. CAMILLO	V.LE V. VENETO	31100	TREVISO	PUBLIC
400	050	VENETO	50080	PARK VILLA NAPOLEON	VIA TERRAGLIO 439/441	31022	PREGANZIOL	PRIVATE
401	050	VENETO	50081	C.C.GIOVANNI XXIII	VIAGIOVANNI XXIII, 7	31050	MONASTIER DI TREVISO	PRIVATE
402	050	VENETO	50209	OSPEDALE DI TREVISO	PIAZZALE OSPEDALE, 1	31100	TREVISO	PUBLIC
403	050	VENETO	50209	OSPEDALE DI TREVISO	PIAZZALE OSPEDALE, 1	31100	TREVISO	PUBLIC
404	050	VENETO	50100	CASA DI CURA SILENO E ANNA RIZZOLA S.P.A	VIA GORIZIA, 1	30027	SAN DONA` DI PIAVE	PRIVATE
405	050	VENETO	50210	OSPEDALE AZIENDA ULSS N. 10	VIA NAZARIO SAURO, 25	30027	SAN DONA` DI PIAVE	PUBLIC
406	050	VENETO	50210	OSPEDALE AZIENDA ULSS N. 10	VIA NAZARIO SAURO, 25	30027	SAN DONA` DI PIAVE	PUBLIC
407	050	VENETO	50210	OSPEDALE AZIENDA ULSS N. 10	VIA NAZARIO SAURO, 25	30027	SAN DONA` DI PIAVE	PUBLIC
408	050	VENETO	50097	OSP.CLASS.FATEBENEFRATELLI	CANNAREGIO 3458	30121	VENEZIA	PUBLIC
409	050	VENETO	50098	OSP.CLASS.VILLASALUS	VIA TERRAGLIO 114	30174	VENEZIA	PUBLIC
410	050	VENETO	50099	CASA DI CURA S.MARCO	VIA ZANOTTO 40	30173	VENEZIA	PUBLIC
411	050	VENETO	50212	OSPEDALE DELL`ULSS VENEZIANA	VIA DON TOSATTO N.147	30170	VENEZIA	PUBLIC
412	050	VENETO	50212	OSPEDALE DELL`ULSS VENEZIANA	VIA DON TOSATTO N.147	30170	VENEZIA	PUBLIC
413	050	VENETO	50212	OSPEDALE DELL`ULSS VENEZIANA	VIA DON TOSATTO N.147	30170	VENEZIA	PUBLIC
414	050	VENETO	50951	I.R.C.C.S. 'S.CAMILLO'	VIA ALBERONI, 70	30126	VENEZIA	PUBLIC
415	050	VENETO	50213	STRUTTURE OSPEDALIERE ULSS 13 - MIRANO	VIA MARIUTTO, 76	30035	MIRANO	PUBLIC
416	050	VENETO	50213	STRUTTURE OSPEDALIERE ULSS 13 - MIRANO	VIA MARIUTTO, 76	30035	MIRANO	PUBLIC
417	050	VENETO	50213	STRUTTURE OSPEDALIERE ULSS 13 - MIRANO	VIA MARIUTTO, 76	30035	MIRANO	PUBLIC
418	050	VENETO	50214	AZIENDA U.L.S.S. N[14	STRADA MADONNA MARINA N. 500	30019	CHIOGGIA	PUBLIC
419	050	VENETO	50214	AZIENDA U.L.S.S. N[14	STRADA MADONNA MARINA N. 500	30019	CHIOGGIA	PUBLIC
420	050	VENETO	50215	OSPEDALE AZ. ULSS N[15 'ALTA PADOVANA'	VIA CASA DI RICOVERO , 40	35013	CITTADELLA	PUBLIC

421	050	VENETO	50215	OSPEDALE AZ. ULSS N[15 'ALTA PADOVANA'	VIA CASA DI RICOVERO , 40	35013	CITTADELLA	PUBLIC
422	050	VENETO	50120	CASA DI CURA 'DIAZ'	VIA A. DIAZ, 9	35193	PADOVA	PRIVATE
423	050	VENETO	50121	CASA DI CURA 'VILLA MARIA'	VIA DELLE MELETTE, 20	35141	PADOVA	PRIVATE
424	050	VENETO	50122	CASA DI CURA 'TRIESTE'	VIA BERGAMO, 10	35142	PADOVA	PRIVATE
425	050	VENETO	50123	CASA DI CURA 'ABANO TERME'	PIAZZA COLOMBO, 1	35031	ABANO TERME	PUBLIC
426	050	VENETO	50124	CASA DI CURA 'PARCO DEI TIGLI'	VIA MONTICELLO, 1	35037	TEOLO	PRIVATE
427	050	VENETO	50147	CASA DI CURA 'MORGAGNI' Srl	VIA CAVAZZANA, 63/7	35123	PADOVA	PRIVATE
428	050	VENETO	50216	OSPEDALE SANT`ANTONIO	VIA FACCIOLATI, 71	35127	PADOVA	PUBLIC
429	050	VENETO	50216	OSPEDALE SANT`ANTONIO	VIA FACCIOLATI, 71	35127	PADOVA	PUBLIC
430	050	VENETO	50217	PRESIDIO OSPEDALIERO ULSS 17	VIA SALUTE, 14/B	35042	ESTE	PUBLIC
431	050	VENETO	50217	PRESIDIO OSPEDALIERO ULSS 17	VIA SALUTE, 14/B	35042	ESTE	PUBLIC
432	050	VENETO	50217	PRESIDIO OSPEDALIERO ULSS 17	VIA SALUTE, 14/B	35042	ESTE	PUBLIC
433	050	VENETO	50130	C.D.C. CITTA' DI ROVIGO	VIA G. SICHIROLLO, 30	45100	ROVIGO	PRIVATE
434	050	VENETO	50131	C.D.C. S.M. MADDALENA	VIA GORIZIA, 2	45030	OCCHIOBELLO	PRIVATE
435	050	VENETO	50218	OSPEDALI AZ ULSS 18 ROVIGO	VIALE TRE MARTIRI 140	45100	ROVIGO	PUBLIC
436	050	VENETO	50218	OSPEDALI AZ ULSS 18 ROVIGO	VIALE TRE MARTIRI 140	45100	ROVIGO	PUBLIC
437	050	VENETO	50132	CASA DI CURA MADONNA DELLA SALUTE	VIA NICOLA BADOLONI 25	45014	PORTO VIRO	PUBLIC
438	050	VENETO	50219	OSPEDALE CIVILE DI ADRIA	PIAZZA DEGLI ETRUSCHI 9	45011	ADRIA	PUBLIC
439	050	VENETO	50018	OSP. CLASSIF. 'S. GIULIANA' SORELLE MIS.	VIA S. GIULIANA, 3	37128	VERONA	PUBLIC
440	050	VENETO	50019	C.D.C. CHIEREGO & PERBELLINI SRL	VIA GAZZERA, 1	37128	VERONA	PRIVATE
441	050	VENETO	50020	C.D.C. VILLA LIETA	VIA ANZANI, 12	37126	VERONA	PRIVATE
442	050	VENETO	50023	C.D.C. SAN FRANCESCO	VIA MONTE ORTIGARA, 21/B	37127	VERONA	PRIVATE
443	050	VENETO	50025	C.DI C. 'VILLA S. CHIARA' SPA	VIA MONTE RECAMAO, 7	37142	VERONA	PRIVATE
444	050	VENETO	50220	AZIENDA ULSS 20 DI VERONA	VIA VALVERDE, 42	37122	VERONA	PUBLIC
445	050	VENETO	50220	AZIENDA ULSS 20 DI VERONA	VIA VALVERDE, 42	37122	VERONA	PUBLIC
446	050	VENETO	50220	AZIENDA ULSS 20 DI VERONA	VIA VALVERDE, 42	37122	VERONA	PUBLIC
447	050	VENETO	50221	AZIENDA U.L.S.S. 21	VIA GIANELLA N 1	37045	LEGNAGO	PUBLIC
448	050	VENETO	50221	AZIENDA U.L.S.S. 21	VIA GIANELLA N 1	37045	LEGNAGO	PUBLIC
449	050	VENETO	50221	AZIENDA U.L.S.S. 21	VIA GIANELLA N 1	37045	LEGNAGO	PUBLIC
450	050	VENETO	50017	OSPEDALE CLASSIFICATO SACRO CUORE - DON	VIA DON SEMPREBON, 5	37024	NEGRAR	PUBLIC
451	050	VENETO	50021	CASA DI CURA VILLA GARDA SPA	VIA MONTE BALDO 89	37016	GARDA	PRIVATE
452	050	VENETO	50022	CASA DI CURA PRIVATA POLISP. DOTT. PEDER	VIA MONTE BALDO 24	37019	PESCHIERA DEL GARDA	PUBLIC
453	050	VENETO	50026	OSPEDALE DON CALABRIA	VIA DON SEMPREBON, 5	37024	NEGRAR	PUBLIC
454	050	VENETO	50222	ALS 22 BUSSOLENGO	VIA CITELLA	37012	BUSSOLENGO	PUBLIC
455	050	VENETO	50222	ALS 22 BUSSOLENGO	VIA CITELLA	37012	BUSSOLENGO	PUBLIC
456	050	VENETO	50222	ALS 22 BUSSOLENGO	VIA CITELLA	37012	BUSSOLENGO	PUBLIC
457	050	VENETO	50222	ALS 22 BUSSOLENGO	VIA CITELLA	37012	BUSSOLENGO	PUBLIC
458	050	VENETO	50901	AZIENDA OSPEDALIERA DI PADOVA	VIA GIUSTINIANI, 1	35127	PADOVA	PUBLIC
459	050	VENETO	50902	AZIENDA OSPEDALIERA DI VERONA	PIAZZALE STEFANI, 1	37126	VERONA	PUBLIC
460	050	VENETO	50902	AZIENDA OSPEDALIERA DI VERONA	PIAZZALE STEFANI, 1	37126	VERONA	PUBLIC
461	050	VENETO	50952	I.R.C.C.S. ISTITUTO ONCOLOGICO VENETO	via gattamelata, 64	35128	PADOVA	PUBLIC
462	060	FRIULI VENEZIA GIULIA	60029	SANATORIO TRIESTINO S.P.A.	VIA DOMENICO ROSSETTI 62	34141	TRIESTE	PRIVATE
463	060	FRIULI VENEZIA GIULIA	60032	PINETA DEL CARSO	VIALE STAZIONE 26	34011	DUINO-AURISINA	PRIVATE
464	060	FRIULI VENEZIA GIULIA	60033	CASA DI CURA SALUS	VIA BONAPARTE 4/6	34123	TRIESTE	PRIVATE
465	060	FRIULI VENEZIA GIULIA	60014	OSPEDALE DI GORIZIA	VIA VITTORIO VENETO 171	34170	GORIZIA	PUBLIC
466	060	FRIULI VENEZIA GIULIA	60016	OSPEDALE DI MONFALCONE	VIA GALVANI 1	34074	MONFALCONE	PUBLIC
467	060	FRIULI VENEZIA GIULIA	60005	O.C. S. MICHELE	VIA RODOLONE 2	33013	GEMONA DEL FRIULI	PUBLIC
468	060	FRIULI VENEZIA GIULIA	60009	O.C. S.ANTONIO ABATE	VIA MORGAGNI 18	33028	TOLMEZZO	PUBLIC
469	060	FRIULI VENEZIA GIULIA	60002	IST.DI MEDICINA FISICA E RIABILITAZIONE	VIA GERVASUTTA, 48	33100	UDINE	PUBLIC
470	060	FRIULI VENEZIA GIULIA	60008	OSPEDALE 'SANT`ANTONIO'	VIALE TRENTO TRIESTE	33038	SAN DANIELE DEL FRIULI	PUBLIC
471	060	FRIULI VENEZIA GIULIA	60011	CASA DI CURA 'CITTA` DI UDINE'	VIALE VENEZIA, 410	33100	UDINE	PRIVATE
472	060	FRIULI VENEZIA GIULIA	60006	OSPEDALE CIVILE DI LATISANA	VIA SABBIONERA N 45	33053	LATISANA	PUBLIC
473	060	FRIULI VENEZIA GIULIA	60007	OSPEDALE CIVILE DI PALMANOVA	VIA NATISONE	33057	PALMANOVA	PUBLIC
474	060	FRIULI VENEZIA GIULIA	60036	OSP. CIV. IMMACOLATA CONCEZIONE	VIA UNITA D'ITALIA 7	33085	MANIAGO	PUBLIC
475	060	FRIULI VENEZIA GIULIA	60038	OSPEDALE S.MARIA DEI BATTUTI	VIA SAVORGNANO ,2	33078	SAN VITO AL TAGLIAMENTO	PUBLIC
476	060	FRIULI VENEZIA GIULIA	60039	OSPED. SAN GIOVANNI DEI BATTUTI	VIA RAFFAELLO 1	33097	SPILIMBERGO	PUBLIC
477	060	FRIULI VENEZIA GIULIA	60040	CASA DI CURA PRIVATA 'S.GIORGIO' SPA	VIA GEMELLI 10	33170	PORDENONE	PRIVATE
478	060	FRIULI VENEZIA GIULIA	60901	I.R.C.C.S. BURLO GAROFOLO	VIA DELL`ISTRIA 65/1	34137	TRIESTE	PUBLIC
479	060	FRIULI VENEZIA GIULIA	60902	CENTRO RIFERIMENTO ONCOLOGICO	VIA PEDEMONTANA OCCIDENTALE 12	33081	AVIANO	PUBLIC
480	060	FRIULI VENEZIA GIULIA	60912	AZIENDA OSPEDALIERA 'S. MARIA DEGLI ANGE	VIA MONTEREALE 24	33170	PORDENONE	PUBLIC
481	060	FRIULI VENEZIA GIULIA	60912	AZIENDA OSPEDALIERA 'S. MARIA DEGLI ANGE	VIA MONTEREALE 24	33170	PORDENONE	PUBLIC
482	060	FRIULI VENEZIA GIULIA	60913	AZ.OSP.UNIV. OSPEDALI RIUNITI DI TRIESTE	VIA FARNETO N. 3	34100	TRIESTE	PUBLIC
483	060	FRIULI VENEZIA GIULIA	60916	AZIENDA OSPEDALIERO-UNIVERSITARIA	P.LE S.MARIA DELLA MISERICORDIA 15	33100	UDINE	PUBLIC

484	070	LIGURIA	70001	PRESIDIO OSPEDALIERO UNIFICATO	VIA AURELIA 97 BUSSANA DI SANREMO	18032	SAN REMO	PUBLIC
485	070	LIGURIA	70001	PRESIDIO OSPEDALIERO UNIFICATO	VIA AURELIA 97 BUSSANA DI SANREMO	18032	SAN REMO	PUBLIC
486	070	LIGURIA	70001	PRESIDIO OSPEDALIERO UNIFICATO	VIA AURELIA 97 BUSSANA DI SANREMO	18032	SAN REMO	PUBLIC
487	070	LIGURIA	70001	PRESIDIO OSPEDALIERO UNIFICATO	VIA AURELIA 97 BUSSANA DI SANREMO	18032	SAN REMO	PUBLIC
488	070	LIGURIA	70008	OSPEDALE S. PAOLO - SAVONA	VIA GENOVA	17100	SAVONA	PUBLIC
489	070	LIGURIA	70009	OSPEDALE S. M. MISERICORDIA - ALBENGA	PIAZZA DEL POPOLO, 13	17031	ALBENGA	PUBLIC
490	070	LIGURIA	70012	OSPEDALE S. GIUSEPPE - CAIRO M.	VIALE MARTIRI DELLA LIBERTA`, 30	17014	CAIRO MONTENOTTE	PUBLIC
491	070	LIGURIA	70014	CASA DI CURA DELLA RIVIERA - SAVONA	VIA GIORDANO, 2	17100	SAVONA	PRIVATE
492	070	LIGURIA	70015	CASA DI CURA SAN MICHELE - ALBENGA	VIALE PONTELUNGO, 79	17031	ALBENGA	PRIVATE
493	070	LIGURIA	70112	CASA DI CURA LA PRESENTAZIONE - LOANO	VIA CARDUCCI, 14	17025	LOANO	PRIVATE
494	070	LIGURIA	70202	C.CLINICO RIAB. 'VILLA ALFIERI - CALICE	Via Puccini 2	17020	CALICE LIGURE	PRIVATE
495	070	LIGURIA	70025	E.O. OSPEDALI GALLIERA	MURA DELLE CAPPUCCINE, 14	16128	GENOVA	PUBLIC
496	070	LIGURIA	70027	PRESIDIO OSPEDALIERO GENOVA NORD	VIA PIERINO NEGROTTO CAMBIASO 62	16159	GENOVA	PUBLIC
497	070	LIGURIA	70027	PRESIDIO OSPEDALIERO GENOVA NORD	VIA PIERINO NEGROTTO CAMBIASO 62	16159	GENOVA	PUBLIC
498	070	LIGURIA	70027	PRESIDIO OSPEDALIERO GENOVA NORD	VIA PIERINO NEGROTTO CAMBIASO 62	16159	GENOVA	PUBLIC
499	070	LIGURIA	70031	PRESIDIO OSPEDALIERO GENOVA PONENTE	PIAZZALE GIANASSO, 4	16158	GENOVA	PUBLIC
500	070	LIGURIA	70031	PRESIDIO OSPEDALIERO GENOVA PONENTE	PIAZZALE GIANASSO, 4	16158	GENOVA	PUBLIC
501	070	LIGURIA	70031	PRESIDIO OSPEDALIERO GENOVA PONENTE	PIAZZALE GIANASSO, 4	16158	GENOVA	PUBLIC
502	070	LIGURIA	70037	OSPEDALE SANT` ANTONIO	VIA A.BIANCHI 1	16036	RECCO	PUBLIC
503	070	LIGURIA	70051	OSPEDALE EVANGELICO INTERNAZIONALE	SALITA SUP. SAN ROCCHINO, 31 A	16122	GENOVA	PUBLIC
504	070	LIGURIA	70065	CASA DI CURA VILLA MONTALLEGRO	Via Monte Zovetto 27	16145	GENOVA	PRIVATE
505	070	LIGURIA	70066	VILLA SERENA S.P.A.	P.ZZA LEOPARDI 18	16145	GENOVA	PRIVATE
506	070	LIGURIA	70067	VILLA S.ANNA S.R.L.	VIA LIRI 27	16145	GENOVA	PRIVATE
507	070	LIGURIA	70074	ISTITUTO CARDIOVASCOLARE CAMOGLI	VIA AURELIA 85	16030	CAMOGLI	PRIVATE
508	070	LIGURIA	70960	FONDAZIONE SALVATORE MAUGERI	VIA MISSOLUNGI 14	16167	GENOVA	PUBLIC
509	070	LIGURIA	70039	PRESIDIO OSPEDALIERO ASL4 CHIAVARESE	VIA DON BOBBIO 25	16033	LAVAGNA	PUBLIC
510	070	LIGURIA	70039	PRESIDIO OSPEDALIERO ASL4 CHIAVARESE	VIA DON BOBBIO 25	16033	LAVAGNA	PUBLIC
511	070	LIGURIA	70039	PRESIDIO OSPEDALIERO ASL4 CHIAVARESE	VIA DON BOBBIO 25	16033	LAVAGNA	PUBLIC
512	070	LIGURIA	70069	CASA DI CURA 'VILLA AZZURRA' S.R.L.	VIA CABRUNA 21	16035	RAPALLO	PRIVATE
513	070	LIGURIA	70058	PRESIDIO OSPEDALIERO LA SPEZIA	VIA VITTORIO VENETO N. 197	19100	LA SPEZIA	PUBLIC
514	070	LIGURIA	70058	PRESIDIO OSPEDALIERO LA SPEZIA	VIA VITTORIO VENETO N. 197	19100	LA SPEZIA	PUBLIC
515	070	LIGURIA	70058	PRESIDIO OSPEDALIERO LA SPEZIA	VIA VITTORIO VENETO N. 197	19100	LA SPEZIA	PUBLIC
516	070	LIGURIA	70059	PRESIDIO OSPEDALIERO SAN BARTOLOMEO	VIA CISA	19038	SARZANA	PUBLIC
517	070	LIGURIA	70070	CASA DI CURA 'ALMA MATER'	CORSO NAZIONALE N. 342	19125	LA SPEZIA	PRIVATE
518	070	LIGURIA	70910	AZ. OSP. 'OSPEDALE S. CORONA'	VIA XXV APRILE, 38	17027	PIETRA LIGURE	PUBLIC
519	070	LIGURIA	70920	AZIENDA OSPEDALIERA VILLA SCASSI	CORSO ONOFRIO SCASSI, 1	16149	GENOVA	PUBLIC
520	070	LIGURIA	70930	AZ.OSP.OSPEDALE S.MARTINO E CL.INIV.CONV	L.GO R.BENZI 10	16132	GENOVA	PUBLIC
521	070	LIGURIA	70940	IST.G.GASLINI	L.GO G. GASLINI	16147	GENOVA	PUBLIC
522	070	LIGURIA	70950	ISTITUTO NAZ. PER LA RICERCA SUL CANCRO	P.LE R. BENZI, 10	16132	GENOVA	PUBLIC
523	080	EMILIA ROMAGNA	80002	VAL TIDONE	VIALE 2 GIUGNO	29015	CASTEL SAN GIOVANNI	PUBLIC
524	080	EMILIA ROMAGNA	80002	VAL TIDONE	VIALE 2 GIUGNO	29015	CASTEL SAN GIOVANNI	PUBLIC
525	080	EMILIA ROMAGNA	80003	BOBBIO	VIA GARIBALDI 1	29022	BOBBIO	PUBLIC
526	080	EMILIA ROMAGNA	80004	PRESIDIO OSPEDALIERO DI PIACENZA	VIA TAVERNA 49	29100	PIACENZA	PUBLIC
527	080	EMILIA ROMAGNA	80006	VAL D'ARDA	VIA ROMA 10	29017	FIORENZUOLA D`ARDA	PUBLIC
528	080	EMILIA ROMAGNA	80006	VAL D'ARDA	VIA ROMA 10	29017	FIORENZUOLA D`ARDA	PUBLIC
529	080	EMILIA ROMAGNA	80006	VAL D'ARDA	VIA ROMA 10	29017	FIORENZUOLA D`ARDA	PUBLIC
530	080	EMILIA ROMAGNA	80203	CASA DI CURA PRIVATA PIACENZA S.P.A.	VIA MORIGI 41	29100	PIACENZA	PRIVATE
531	080	EMILIA ROMAGNA	80204	CASA DI CURA PRIVATA S. ANTONINO S.R.L	VIALE MALTA 4	29100	PIACENZA	PRIVATE
532	080	EMILIA ROMAGNA	80205	CASA DI CURA S.GIACOMO S.R.L.	V.LE S.BONO 3	29028	PONTE DELL`OLIO	PRIVATE
533	080	EMILIA ROMAGNA	80013	FIDENZA-SAN SECONDO	VIA DON TINCATI 5	43036	FIDENZA	PUBLIC
534	080	EMILIA ROMAGNA	80013	FIDENZA-SAN SECONDO	VIA DON TINCATI 5	43036	FIDENZA	PUBLIC
535	080	EMILIA ROMAGNA	80015	BORGO VAL DI TARO	VIA BENEFATTORI N. 12	43043	BORGO VAL DI TARO	PUBLIC
536	080	EMILIA ROMAGNA	80206	CITTA' DI PARMA	PIAZZA ATHOS MAESTRI N. 5	43100	PARMA	PRIVATE
537	080	EMILIA ROMAGNA	80207	PICCOLE FIGLIE	VIA PO 1	43100	PARMA	PRIVATE
538	080	EMILIA ROMAGNA	80208	VILLA MARIA LUIGIA	VIA MONTEPELATO NORD 41-MONTICELLI T	43023	MONTECHIARUGOLO	PRIVATE
539	080	EMILIA ROMAGNA	80209	VILLA IGEA - PR	VIALE BERENINI 2	34039	SALSOMAGGIORE TERME	PRIVATE
540	080	EMILIA ROMAGNA	80210	VAL PARMA	VIA XX SETTEMBRE 22	43013	LANGHIRANO	PRIVATE
541	080	EMILIA ROMAGNA	80253	FONDAZIONE DON CARLO GNOCCHI	PIAZZALE DEI SERVI 3	43100	PARMA	PRIVATE
542	080	EMILIA ROMAGNA	80254	CARDINAL FERRARI	VIA IV NOVEMBRE 21	43012	FONTANELLATO	PRIVATE
543	080	EMILIA ROMAGNA	80021	PRESIDIO OSPEDALIERO PROVINCIALE DI R.E.	VIA AMENDOLA 2	42100	REGGIO NELL`EMILIA	PUBLIC
544	080	EMILIA ROMAGNA	80021	PRESIDIO OSPEDALIERO PROVINCIALE DI R.E.	VIA AMENDOLA 2	42100	REGGIO NELL`EMILIA	PUBLIC
545	080	EMILIA ROMAGNA	80021	PRESIDIO OSPEDALIERO PROVINCIALE DI R.E.	VIA AMENDOLA 2	42100	REGGIO NELL`EMILIA	PUBLIC
546	080	EMILIA ROMAGNA	80021	PRESIDIO OSPEDALIERO PROVINCIALE DI R.E.	VIA AMENDOLA 2	42100	REGGIO NELL`EMILIA	PUBLIC

547	080	EMILIA ROMAGNA	80021	PRESIDIO OSPEDALIERO PROVINCIALE DI R.E.	VIA AMENDOLA 2	42100	REGGIO NELL`EMILIA	PUBLIC
548	080	EMILIA ROMAGNA	80211	SALUS S.P.A	VIA ULDERICO LEVI N.7	42100	REGGIO NELL`EMILIA	PRIVATE
549	080	EMILIA ROMAGNA	80212	CASA DI CURA PRIVATA VILLA VERDE S.R.L.	VIALE LELIO BASSO 1	42100	REGGIO NELL`EMILIA	PRIVATE
550	080	EMILIA ROMAGNA	80031	PRESIDIO OSPED.PROV. NUOVO S.AGOSTINO	VIA GIARDINI, 1355 - BAGGIOVARA	41100	MODENA	PUBLIC
551	080	EMILIA ROMAGNA	80031	PRESIDIO OSPED.PROV. NUOVO S.AGOSTINO	VIA GIARDINI, 1355 - BAGGIOVARA	41100	MODENA	PUBLIC
552	080	EMILIA ROMAGNA	80031	PRESIDIO OSPED.PROV. NUOVO S.AGOSTINO	VIA GIARDINI, 1355 - BAGGIOVARA	41100	MODENA	PUBLIC
553	080	EMILIA ROMAGNA	80031	PRESIDIO OSPED.PROV. NUOVO S.AGOSTINO	VIA GIARDINI, 1355 - BAGGIOVARA	41100	MODENA	PUBLIC
554	080	EMILIA ROMAGNA	80031	PRESIDIO OSPED.PROV. NUOVO S.AGOSTINO	VIA GIARDINI, 1355 - BAGGIOVARA	41100	MODENA	PUBLIC
555	080	EMILIA ROMAGNA	80031	PRESIDIO OSPED.PROV. NUOVO S.AGOSTINO	VIA GIARDINI, 1355 - BAGGIOVARA	41100	MODENA	PUBLIC
556	080	EMILIA ROMAGNA	80031	PRESIDIO OSPED.PROV. NUOVO S.AGOSTINO	VIA GIARDINI, 1355 - BAGGIOVARA	41100	MODENA	PUBLIC
557	080	EMILIA ROMAGNA	80096	NUOVO OSPEDALE DI SASSUOLO S.p.A.	VIA FRANCESCO RUINI, 2	41049	SASSUOLO	PUBLIC
558	080	EMILIA ROMAGNA	80213	HESPERIA HOSPITAL MODENA S.r.l.	VIA ARQUA`, 80/A	41100	MODENA	PRIVATE
559	080	EMILIA ROMAGNA	80214	PROF. FOGLIANI CASA DI CURA S.r.l.	VIA LANA, 1	41100	MODENA	PRIVATE
560	080	EMILIA ROMAGNA	80215	VILLA IGEA S.p.A. CASA DI CURA	VIA STRADELLA, 73	41040	MODENA	PRIVATE
561	080	EMILIA ROMAGNA	80216	VILLA ROSA OSPEDALE PRIVATO ACCREDITATO	VIA FRATELLI ROSSELLI, 83	41100	MODENA	PRIVATE
562	080	EMILIA ROMAGNA	80218	VILLA PINETA S.r.l.	VIA GAIATO, 127	41026	PAVULLO NEL FRIGNANO	PRIVATE
563	080	EMILIA ROMAGNA	80053	PRESIDIO UNICO OSP AZIENDA DI BOLOGNA	LARGO BARTOLO NIGRISOLI 2	40133	BOLOGNA	PUBLIC
564	080	EMILIA ROMAGNA	80053	PRESIDIO UNICO OSP AZIENDA DI BOLOGNA	LARGO BARTOLO NIGRISOLI 2	40133	BOLOGNA	PUBLIC
565	080	EMILIA ROMAGNA	80053	PRESIDIO UNICO OSP AZIENDA DI BOLOGNA	LARGO BARTOLO NIGRISOLI 2	40133	BOLOGNA	PUBLIC
566	080	EMILIA ROMAGNA	80053	PRESIDIO UNICO OSP AZIENDA DI BOLOGNA	LARGO BARTOLO NIGRISOLI 2	40133	BOLOGNA	PUBLIC
567	080	EMILIA ROMAGNA	80053	PRESIDIO UNICO OSP AZIENDA DI BOLOGNA	LARGO BARTOLO NIGRISOLI 2	40133	BOLOGNA	PUBLIC
568	080	EMILIA ROMAGNA	80053	PRESIDIO UNICO OSP AZIENDA DI BOLOGNA	LARGO BARTOLO NIGRISOLI 2	40133	BOLOGNA	PUBLIC
569	080	EMILIA ROMAGNA	80053	PRESIDIO UNICO OSP AZIENDA DI BOLOGNA	LARGO BARTOLO NIGRISOLI 2	40133	BOLOGNA	PUBLIC
570	080	EMILIA ROMAGNA	80053	PRESIDIO UNICO OSP AZIENDA DI BOLOGNA	LARGO BARTOLO NIGRISOLI 2	40133	BOLOGNA	PUBLIC
571	080	EMILIA ROMAGNA	80053	PRESIDIO UNICO OSP AZIENDA DI BOLOGNA	LARGO BARTOLO NIGRISOLI 2	40133	BOLOGNA	PUBLIC
572	080	EMILIA ROMAGNA	80219	VILLA CHIARA	VIA PORRETTANA, 170	40033	CASALECCHIO DI RENO	PRIVATE
573	080	EMILIA ROMAGNA	80220	PROF. NOBILI	VIA FIERA 25	40035	CASTIGLIONE DEI PEPOLI	PRIVATE
574	080	EMILIA ROMAGNA	80221	VILLA ERBOSA SPA	VIA DELL'ARCOVEGGIO 50/2	40129	BOLOGNA	PRIVATE
575	080	EMILIA ROMAGNA	80222	CASA DI CURA NIGRISOLI SPA	VIALE ERCOLANI 7 BIS	40138	BOLOGNA	PRIVATE
576	080	EMILIA ROMAGNA	80223	CASA DI CURA VILLA TORRI	VIALE FILOPANTI 12	40126	BOLOGNA	PRIVATE
577	080	EMILIA ROMAGNA	80224	CASA DI CURA AI COLLI S.R.L.	VIA S MAMOLO 158	40136	BOLOGNA	PRIVATE
578	080	EMILIA ROMAGNA	80225	CASA DI CURA MADRE FORTUNATA TONIOLO	VIA TOSCANA 34	40141	BOLOGNA	PRIVATE
579	080	EMILIA ROMAGNA	80226	CASA DI CURA VILLALBA S.R.L.	VIA DI RONCRIO 25	40136	BOLOGNA	PRIVATE
580	080	EMILIA ROMAGNA	80227	OSPEDALE PRIVATO ACCRED.VILLA BARUZZIANA	VIA DELL'OSSERVANZA 19	40136	BOLOGNA	PRIVATE
581	080	EMILIA ROMAGNA	80228	CASA DI CURA VILLA BELLOMBRA	VIA BELLOMBRA 24	40136	BOLOGNA	PRIVATE
582	080	EMILIA ROMAGNA	80229	CASA DI CURA VILLA LAURA S.P.A.	VIA EMILIA LEVANTE 137	40139	BOLOGNA	PRIVATE
583	080	EMILIA ROMAGNA	80231	CASA DI CURA VILLA REGINA S.P.A.	VIA CASTIGLIONE 115	40133	BOLOGNA	PRIVATE
584	080	EMILIA ROMAGNA	80255	OSPEDALE PRIVATO S.VIOLA S.R.L.	VIA DELLA FERRIERA 10	40133	BOLOGNA	PRIVATE
585	080	EMILIA ROMAGNA	80044	PRESIDIO OSPED. IMOLA - CASTEL S. PIETRO	VIA MONTERICCO, 4	40026	IMOLA	PUBLIC
586	080	EMILIA ROMAGNA	80044	PRESIDIO OSPED. IMOLA - CASTEL S. PIETRO	VIA MONTERICCO, 4	40026	IMOLA	PUBLIC
587	080	EMILIA ROMAGNA	80045	OSPEDALE MONTECATONE R.I. S.P.A.	VIA MONTECATONE, 37	40026	IMOLA	PUBLIC
588	080	EMILIA ROMAGNA	80068	PRESIDIO UNICO OSPEDALIERO	VIA R. FELLETTI, 2	44022	COMACCHIO	PUBLIC
589	080	EMILIA ROMAGNA	80068	PRESIDIO UNICO OSPEDALIERO	VIA R. FELLETTI, 2	44022	COMACCHIO	PUBLIC
590	080	EMILIA ROMAGNA	80068	PRESIDIO UNICO OSPEDALIERO	VIA R. FELLETTI, 2	44022	COMACCHIO	PUBLIC
591	080	EMILIA ROMAGNA	80068	PRESIDIO UNICO OSPEDALIERO	VIA R. FELLETTI, 2	44022	COMACCHIO	PUBLIC
592	080	EMILIA ROMAGNA	80068	PRESIDIO UNICO OSPEDALIERO	VIA R. FELLETTI, 2	44022	COMACCHIO	PUBLIC
593	080	EMILIA ROMAGNA	80068	PRESIDIO UNICO OSPEDALIERO	VIA R. FELLETTI, 2	44022	COMACCHIO	PUBLIC
594	080	EMILIA ROMAGNA	80235	CASA DI CURA QUISISANA S.R.L.	VIALE CAVOUR, 128	44100	FERRARA	PRIVATE
595	080	EMILIA ROMAGNA	80236	CASA DI CURA SALUS S.R.L.	VIA ARIANUOVA, 38	44100	FERRARA	PRIVATE
596	080	EMILIA ROMAGNA	80414	FERRARA DAY SURGERY S.R.L.	VIA GIOVANNI VERGA, 17 - 17/A	44100	FERRARA	PRIVATE
597	080	EMILIA ROMAGNA	80072	RAVENNA	VIALE RANDI, 5	48100	RAVENNA	PUBLIC
598	080	EMILIA ROMAGNA	80079	LUGO	VIALE DANTE, 10	48022	LUGO	PUBLIC
599	080	EMILIA ROMAGNA	80082	FAENZA	VIALE STRADONE, 9	48018	FAENZA	PUBLIC
600	080	EMILIA ROMAGNA	80237	DOMUS NOVA	VIA PAVIRANI 44	48100	RAVENNA	PRIVATE
601	080	EMILIA ROMAGNA	80238	SAN FRANCESCO	VIA AMALASUNTA 20	48100	RAVENNA	PRIVATE
602	080	EMILIA ROMAGNA	80239	VILLA MARIA CECILIA	VIA CORRIERA 1	48010	COTIGNOLA	PRIVATE
603	080	EMILIA ROMAGNA	80240	SAN PIER DAMIANO	VIA ISONZO 10	48018	FAENZA	PRIVATE
604	080	EMILIA ROMAGNA	80241	VILLA STACCHINI S.P.A.	VIALE STRADONE 18	48018	FAENZA	PRIVATE
605	080	EMILIA ROMAGNA	80242	VILLA AZZURRA	VIA CAVINA 9	48025	RIOLO TERME	PRIVATE
606	080	EMILIA ROMAGNA	80085	PRESIDIO OSPEDALIERO FORLI`	VIA C. FORLANINI, 34	47100	FORLI`	PUBLIC
607	080	EMILIA ROMAGNA	80085	PRESIDIO OSPEDALIERO FORLI`	VIA C. FORLANINI, 34	47100	FORLI`	PUBLIC
608	080	EMILIA ROMAGNA	80085	PRESIDIO OSPEDALIERO FORLI`	VIA C. FORLANINI, 34	47100	FORLI`	PUBLIC
609	080	EMILIA ROMAGNA	80121	I.R.S.T. SRL ISTITUTO SCIENT.ROMAGNOLO	VIA P. MARONCELLI N. 40/42	47014	MELDOLA	PUBLIC

610	080	EMILIA ROMAGNA	80243	OSP.PRIVATO ACCRED. VILLA IGEA S.P.A.	VIALE ANTONIO GRAMSCI, 42-44-46	47100	FORLI`	PRIVATE
611	080	EMILIA ROMAGNA	80244	VILLA SERENA	VIA DEL CAMALDOLINO N 8	47100	FORLI`	PRIVATE
612	080	EMILIA ROMAGNA	80091	CESENA	V.LE GHIROTTI, 286	47023	CESENA	PUBLIC
613	080	EMILIA ROMAGNA	80091	CESENA	V.LE GHIROTTI, 286	47023	CESENA	PUBLIC
614	080	EMILIA ROMAGNA	80091	CESENA	V.LE GHIROTTI, 286	47023	CESENA	PUBLIC
615	080	EMILIA ROMAGNA	80245	MALATESTA NOVELLO	VIA RENATO SERRA, 2	47023	CESENA	PRIVATE
616	080	EMILIA ROMAGNA	80246	SAN LORENZINO	V. NATALE DELL`AMORE, 15	47023	CESENA	PRIVATE
617	080	EMILIA ROMAGNA	80095	PRESIDIO OSPEDALIERO RIMINI-SANTARCANGEL	VIALE SETTEMBRINI, 2	47900	RIMINI	PUBLIC
618	080	EMILIA ROMAGNA	80095	PRESIDIO OSPEDALIERO RIMINI-SANTARCANGEL	VIALE SETTEMBRINI, 2	47900	RIMINI	PUBLIC
619	080	EMILIA ROMAGNA	80100	PRESIDIO OSPEDALIERO RICCIONE-CATTOLICA	VIA FORMIA, 14	47838	RICCIONE	PUBLIC
620	080	EMILIA ROMAGNA	80100	PRESIDIO OSPEDALIERO RICCIONE-CATTOLICA	VIA FORMIA, 14	47838	RICCIONE	PUBLIC
621	080	EMILIA ROMAGNA	80247	SOL ET SALUS	VIA S. SALVADOR, 204	47812	RIMINI	PRIVATE
622	080	EMILIA ROMAGNA	80249	CASA DI CURA VILLA MARIA	VIALE MATTEOTTI N. 24	47900	RIMINI	PRIVATE
623	080	EMILIA ROMAGNA	80250	LUCE SUL MARE	VIALE PINZON 312/314	47813	BELLARIA-IGEA MARINA	PRIVATE
624	080	EMILIA ROMAGNA	80251	VILLA SALUS S.R.L.	VIA PORTO PALOS N. 93	47900	RIMINI	PRIVATE
625	080	EMILIA ROMAGNA	80252	CASA DI CURA PROF. E. MONTANARI	VIA ROMA 7	47833	MORCIANO DI ROMAGNA	PRIVATE
626	080	EMILIA ROMAGNA	80902	AZIENDA OSPEDALIERA DI PARMA	VIA GRAMSCI, 14	43100	PARMA	PUBLIC
627	080	EMILIA ROMAGNA	80903	ARCISPEDALE S. MARIA NUOVA	VIALE RISORGIMENTO N 80	42100	REGGIO NELL`EMILIA	PUBLIC
628	080	EMILIA ROMAGNA	80904	AZIENDA OSPEDALIERA POLICLINICO	VIA DEL POZZO, 71	41100	MODENA	PUBLIC
629	080	EMILIA ROMAGNA	80908	S.ORSOLA-MALPIGHI - BO	VIA MASSARENTI 9	40138	BOLOGNA	PUBLIC
630	080	EMILIA ROMAGNA	80909	AZIENDA OSPEDALIERO-UNIVERSITARIA DI FER	C.SO GIOVECCA 203	44100	FERRARA	PUBLIC
631	080	EMILIA ROMAGNA	80960	II.OO. RIZZOLI - BO	VIA DI BARBIANO 1/10	40136	BOLOGNA	PUBLIC
632	090	TOSCANA	90601	PRESIDIO OSPEDALIERO DELLA LUNIGIANA	VIA DON MINZONI (SEDE AZIENDALE)	54033	CARRARA	PUBLIC
633	090	TOSCANA	90601	PRESIDIO OSPEDALIERO DELLA LUNIGIANA	VIA DON MINZONI (SEDE AZIENDALE)	54033	CARRARA	PUBLIC
634	090	TOSCANA	90602	PRESIDIO OSPEDALIERO DI MASSA E CARRARA	VIA DON MINZONI 3	54033	CARRARA	PUBLIC
635	090	TOSCANA	90602	PRESIDIO OSPEDALIERO DI MASSA E CARRARA	VIA DON MINZONI 3	54033	CARRARA	PUBLIC
636	090	TOSCANA	90204	CASA DI CURA M.D. BARBANTINI	VIA DEL CALCIO 2	55100	LUCCA	PRIVATE
637	090	TOSCANA	90205	CASA DI CURA S.ZITA	VIA DEL PANIFICIO 13	55100	LUCCA	PRIVATE
638	090	TOSCANA	90206	CASA DI CURA VILLE DI NOZZANO	VIA BORDOGNA 144 - NOZZANO	55050	LUCCA	PRIVATE
639	090	TOSCANA	90603	PRESIDIO OSPEDALIERO VALLE DEL SERCHIO	VIA DELL'OSPEDALE	55032	CASTELNUOVO DI GARFAGNANA	PUBLIC
640	090	TOSCANA	90603	PRESIDIO OSPEDALIERO VALLE DEL SERCHIO	VIA DELL'OSPEDALE	55032	CASTELNUOVO DI GARFAGNANA	PUBLIC
641	090	TOSCANA	90604	PRESIDIO OSPEDALIERO PIANA DI LUCCA	LOCALITA' CAMPO DI MARTE-LUCCA	55100	LUCCA	PUBLIC
642	090	TOSCANA	90207	CASA DI CURA S. RITA	V. MANIN N. 29 MONTECATINI T.	51016	MONTECATINI-TERME	PRIVATE
643	090	TOSCANA	90606	SPEDALI RIUNITI	V.LE G. MATTEOTTI PISTOIA	51100	PISTOIA	PUBLIC
644	090	TOSCANA	90606	SPEDALI RIUNITI	V.LE G. MATTEOTTI PISTOIA	51100	PISTOIA	PUBLIC
645	090	TOSCANA	90607	OSPEDALE DELLA VALDINIEVOLE	V. C. BATTISTI PESCIA	51017	PESCIA	PUBLIC
646	090	TOSCANA	90210	CASA DI CURA VILLA FIORITA	VIA CANTAGALLO 56	59100	PRATO	PRIVATE
647	090	TOSCANA	90608	OSPEDALE MISERICORDIA E DOLCE	P.ZA OSPEDALE 5	59100	PRATO	PUBLIC
648	090	TOSCANA	90231	CASA DI CURA SUORE DELL'ADDOLORATA	V. MANZONI 13	56100	PISA	PRIVATE
649	090	TOSCANA	90232	CASA DI CURA PRIVATA DELLA MISERICORDIA	VIA CARLO CAMMEO , 24 -NAVACCHIO	56023	CASCINA	PRIVATE
650	090	TOSCANA	90233	CASA DI CURA PRIVATA SAN ROSSORE S.R.L.	VIALE DELLE CASCINE , 152/F	56122	PISA	PRIVATE
651	090	TOSCANA	90609	PRESIDIO OSPEDALIERO "S.MARIA MADDALENA"	BORGO S.LAZZARO,5	56048	VOLTERRA	PUBLIC
652	090	TOSCANA	90610	PRESIDIO OSPEDALIERO "FELICE LOTTI"	VIA ROMA,180	56025	PONTEDERA	PUBLIC
653	090	TOSCANA	90636	SPDC PISANO	VIA ROMA	56100	PISA	PUBLIC
654	090	TOSCANA	90908	CENTRO RIABILITAZIONE MOTORIA INAIL	BORGO SAN LAZZARO , 5	56048	VOLTERRA	PUBLIC
655	090	TOSCANA	90909	AUXILIUM VITAE VOLTERRA SPA	BORGO SAN LAZZARO, 5	56048	VOLTERRA	PUBLIC
656	090	TOSCANA	90234	CASA DI CURA VILLA TIRRENA	VIA MONTEBELLO 102	57126	LIVORNO	PRIVATE
657	090	TOSCANA	90612	OSPEDALE CECINA	VIA DELLA LADRONAIA	57023	CECINA	PUBLIC
658	090	TOSCANA	90613	OSPEDALE LIVORNO	VIALE ALFIERI	57100	LIVORNO	PUBLIC
659	090	TOSCANA	90614	OSPEDALE PIOMBINO	VIA FORLANINI, 24	57025	PIOMBINO	PUBLIC
660	090	TOSCANA	90615	OSPEDALE DI PORTOFERRAIO	LOC. S.ROCCO	57027	PORTOFERRAIO	PUBLIC
661	090	TOSCANA	90239	CASA DI CURA RUGANI	PIAZZA MATTEOTTI 33	53100	SIENA	PRIVATE
662	090	TOSCANA	90634	SERVIZIO PSICHIATRICO DIAGNOSI E CURA	VIALE BRACCI 16	53100	SIENA	PUBLIC
663	090	TOSCANA	90637	PRESIDIO OSPEDALIERO AMIATA SENESE	VIA TRENTO	53021	ABBADIA SAN SALVATORE	PUBLIC
664	090	TOSCANA	90638	OSPEDALE DELL'ALTA VAL D'ELSA	LOC.CAMPOSTAGGIA	53036	POGGIBONSI	PUBLIC
665	090	TOSCANA	90639	OSPEDALI RIUNITI DELLA VAL DI CHIANA	VIA PROVINCIALE MONTEPULCIANO, 5	53045	MONTEPULCIANO	PUBLIC
666	090	TOSCANA	90236	CASA DI CURA POGGIO DEL SOLE SRL	VIA FRA' GUITTONE, 2	52100	AREZZO	PRIVATE
667	090	TOSCANA	90237	CASA DI CURA S.GIUSEPPE	VIA A. SAFFI, 33	52100	AREZZO	PRIVATE
668	090	TOSCANA	90618	OSPEDALE DEL CASENTINO	VIALE FILIPPO TURATI	52011	BIBBIENA	PUBLIC
669	090	TOSCANA	90619	OSPEDALE DELLA VALTIBERINA	VIA SANTI DI TITO, 24	52037	SANSEPOLCRO	PUBLIC
670	090	TOSCANA	90621	OSPEDALE AREA ARETINA NORD	VIA P. NENNI	52100	AREZZO	PUBLIC
671	090	TOSCANA	90641	OSPEDALE NUOVO VALDARNO	LOC. GRUCCIA	52025	MONTEVARCHI	PUBLIC
672	090	TOSCANA	90643	NUOVO OSPEDALE VALDICHIANA S. MARGHERITA	LOC. FRATTA	52044	CORTONA	PUBLIC

673	090	TOSCANA	90911	CENTRO RIABILITAZIONE TERRANUOVA B. SPA	PI.ZZA VOLONTARIATO	52025	MONTEVARCHI	PUBLIC
674	090	TOSCANA	90623	OSPEDALE "S.ANDREA" MASSA MARITTIMA	VIA RISORGIMENTO, 43	58024	MASSA MARITTIMA	PUBLIC
675	090	TOSCANA	90624	OSPEDALE DELLE COLLINE DELL'ALBEGNA	LUNGOLAGO DEI PESCATORI	58045	ORBETELLO	PUBLIC
676	090	TOSCANA	90624	OSPEDALE DELLE COLLINE DELL'ALBEGNA	LUNGOLAGO DEI PESCATORI	58045	ORBETELLO	PUBLIC
677	090	TOSCANA	90625	OSPEDALE CIVILE DI CASTEL DEL PIANO	VIA DANTE ALIGHIERI	58033	CASTEL DEL PIANO	PUBLIC
678	090	TOSCANA	90626	OSPEDALE DELLA MISERICORDIA GROSSETO	VIA SENESE, 169	58100	GROSSETO	PUBLIC
679	090	TOSCANA	90212	CASA DI CURA VILLA SANTA CHIARA	PIAZZA INDIPENDENZA, 11	50129	FIRENZE	PRIVATE
680	090	TOSCANA	90213	CASA DI CURA VILLA DONATELLO SPA	P.LE DONATELLO N. 14	50132	FIRENZE	PRIVATE
681	090	TOSCANA	90214	CASA DI CURA VILLA CHERUBINI	VIA L.CHERUBINI 8	50121	FIRENZE	PRIVATE
682	090	TOSCANA	90215	VILLA MARIA BEATRICE	VIA ALESSANDRO MANZONI, 12	50121	FIRENZE	PRIVATE
683	090	TOSCANA	90216	VILLA DEI PINI S.R.L.	VIA UGO FOSCOLO 78	50124	FIRENZE	PRIVATE
684	090	TOSCANA	90218	CASA DI CURA ULIVELLA E GLICINI	VIA DEL PERGOLINO 416	50139	FIRENZE	PRIVATE
685	090	TOSCANA	90219	CASA DI CURA VILLA MARIA TERESA	VIA DELLA CERNAIA 18	50129	FIRENZE	PRIVATE
686	090	TOSCANA	90220	CASA DI CURA VILLANOVA	VIA DI CAREGGI 38	50139	FIRENZE	PRIVATE
687	090	TOSCANA	90221	CASA DI CURA IL PERGOLINO SPA	VIA DEL PERGOLINO 9	50139	FIRENZE	PRIVATE
688	090	TOSCANA	90224	ISTITUTO REUMATOLOGICO MUNARI S.P.A.	VIALE MAZZINI 38	50132	FIRENZE	PRIVATE
689	090	TOSCANA	90225	VILLA DELLE TERME	VIALE MAZZINI, 43	50132	FIRENZE	PRIVATE
690	090	TOSCANA	90227	CASA DI CURA SAN CAMILLO	VIA G. MARCONI, 4	50131	FIRENZE	PRIVATE
691	090	TOSCANA	90228	CASA DI CURA POGGIO SERENO	VIA BENEDETTO DA MAIANO, 14	50016	FIESOLE	PRIVATE
692	090	TOSCANA	90229	VILLA DELLE TERME SPA	VIA CASSIA, 217 LOC. FALCIANI	50023	IMPRUNETA	PRIVATE
693	090	TOSCANA	90230	CASA DI CURA VAL DI SIEVE	VIA FORLIVESE, 122	50060	PELAGO	PRIVATE
694	090	TOSCANA	90240	CASA DI CURA FRATE SOLE	VIA SAN ROMOLO 109	50063	FIGLINE VALDARNO	PRIVATE
695	090	TOSCANA	90627	OSPEDALE DI BORGO SAN LORENZO	VIALE DELLA RESISTENZA	50032	BORGO SAN LORENZO	PUBLIC
696	090	TOSCANA	90629	OSPEDALE FIORENTINO SUD-EST	VIA DELL'ANTELLA 58	50011	BAGNO A RIPOLI	PUBLIC
697	090	TOSCANA	90629	OSPEDALE FIORENTINO SUD-EST	VIA DELL'ANTELLA 58	50011	BAGNO A RIPOLI	PUBLIC
698	090	TOSCANA	90632	OSPEDALE FIORENTINO	PIAZZA SANTA MARIA NUOVA 1	50122	FIRENZE	PUBLIC
699	090	TOSCANA	90632	OSPEDALE FIORENTINO	PIAZZA SANTA MARIA NUOVA 1	50122	FIRENZE	PUBLIC
700	090	TOSCANA	90632	OSPEDALE FIORENTINO	PIAZZA SANTA MARIA NUOVA 1	50122	FIRENZE	PUBLIC
701	090	TOSCANA	90632	OSPEDALE FIORENTINO	PIAZZA SANTA MARIA NUOVA 1	50122	FIRENZE	PUBLIC
702	090	TOSCANA	90910	FONDAZIONE DON CARLO GNOCCHI - ONLUS	VIA IMPRUNETANA 124 - POZZOLATICO	50020	IMPRUNETA	PUBLIC
703	090	TOSCANA	90235	CASA DI CURA "LEONARDO"	VIA P.GROCCO,136 LOC. SOVIGLIANA	50059	VINCI	PRIVATE
704	090	TOSCANA	90642	PRESIDIO OSPEDALIERO ASL 11 EMPOLI	VIALE BOCCACCIO	50053	EMPOLI	PUBLIC
705	090	TOSCANA	90642	PRESIDIO OSPEDALIERO ASL 11 EMPOLI	VIALE BOCCACCIO	50053	EMPOLI	PUBLIC
706	090	TOSCANA	90642	PRESIDIO OSPEDALIERO ASL 11 EMPOLI	VIALE BOCCACCIO	50053	EMPOLI	PUBLIC
707	090	TOSCANA	90642	PRESIDIO OSPEDALIERO ASL 11 EMPOLI	VIALE BOCCACCIO	50053	EMPOLI	PUBLIC
708	090	TOSCANA	90202	CASA DI CURA "S.CAMILLO"	VIA P.IGNAZIO, 37	55042	FORTE DEI MARMI	PRIVATE
709	090	TOSCANA	90203	CASA DI CURA "BARBANTINI"	VIA FOSSO GUIDARIO LOC.BICCHIO	55049	VIAREGGIO	PRIVATE
710	090	TOSCANA	90640	OSPEDALE VERSILIA	VIA AURELIA, 335	55043	CAMAIORE	PUBLIC
711	090	TOSCANA	90901	AZIENDA OSPEDALIERO-UNIVERSITARIA PISANA	VIA ROMA 67	56126	PISA	PUBLIC
712	090	TOSCANA	90906	FONDAZIONE STELLA MARIS - CALAMBRONE	VIALE DEL TIRRENO 331 - CALAMBRONE	56018	PISA	PUBLIC
713	090	TOSCANA	90907	AZIENDA ENTE DI RICERCA CREAS IFC-CNR	VIA TRIESTE 41	56126	PISA	PUBLIC
714	090	TOSCANA	90907	AZIENDA ENTE DI RICERCA CREAS IFC-CNR	VIA TRIESTE 41	56126	PISA	PUBLIC
715	090	TOSCANA	90902	SPEDALI RIUNITI	V.LE BRACCI - LOC.LE SCOTTE	53100	SIENA	PUBLIC
716	090	TOSCANA	90903	AZ. OSPEDALIERO - UNIVERSITARIA CAREGGI	VIALE PIERACCINI, 17	50139	FIRENZE	PUBLIC
717	090	TOSCANA	90904	AZIENDA OSPEDALIERA MEYER	VIALE PIERACCINI 24	50139	FIRENZE	PUBLIC
718	100	UMBRIA	100801	PRESIDIO OSPEDALIERO C. CASTELLO	VOCABOLO CHIOCCOLO	06012	CITTA` DI CASTELLO	PUBLIC
719	100	UMBRIA	100801	PRESIDIO OSPEDALIERO C. CASTELLO	VOCABOLO CHIOCCOLO	06012	CITTA` DI CASTELLO	PUBLIC
720	100	UMBRIA	100808	PRESIDIO OSPEDALIERO GUBBIO	LARGO SAN FRANCESCO 7/A	06024	GUBBIO	PUBLIC
721	100	UMBRIA	100808	PRESIDIO OSPEDALIERO GUBBIO	LARGO SAN FRANCESCO 7/A	06024	GUBBIO	PUBLIC
722	100	UMBRIA	100601	SERVIZIO PSICHIATRICO DIAGNOSI E CURA	VIA ENRICO DAL POZZO	06100	PERUGIA	PUBLIC
723	100	UMBRIA	100701	CASA DI CURA VILLA FIORITA S.R.L.	VIA XX SETTEMBRE, 55	06124	PERUGIA	PRIVATE
724	100	UMBRIA	100702	CASA DI CURA PORTA SOLE	PIAZZA MICHELOTTI, 4	06100	PERUGIA	PRIVATE
725	100	UMBRIA	100703	SAGISC S.R.L.-CASA DI CURA MADONNA DEGLI	VIA SOLATIA, 3	06121	PERUGIA	PRIVATE
726	100	UMBRIA	100704	CASA DI CURA LIOTTI S.P.A.	VIA SERAFINO SIEPI, 11	06123	PERUGIA	PRIVATE
727	100	UMBRIA	100803	PRESIDIO OSPEDALIERO USL N. 2	VIA GALLENGA 2	06127	PERUGIA	PUBLIC
728	100	UMBRIA	100803	PRESIDIO OSPEDALIERO USL N. 2	VIA GALLENGA 2	06127	PERUGIA	PUBLIC
729	100	UMBRIA	100803	PRESIDIO OSPEDALIERO USL N. 2	VIA GALLENGA 2	06127	PERUGIA	PUBLIC
730	100	UMBRIA	100803	PRESIDIO OSPEDALIERO USL N. 2	VIA GALLENGA 2	06127	PERUGIA	PUBLIC
731	100	UMBRIA	100803	PRESIDIO OSPEDALIERO USL N. 2	VIA GALLENGA 2	06127	PERUGIA	PUBLIC
732	100	UMBRIA	100803	PRESIDIO OSPEDALIERO USL N. 2	VIA GALLENGA 2	06127	PERUGIA	PUBLIC
733	100	UMBRIA	100705	CASA DI CURA VILLA AURORA SRL	VIA ARNO N. 2	06034	FOLIGNO	PRIVATE
734	100	UMBRIA	100805	POLO OSPEDALIERO SPOLETO	VIA LORETO N.3	06049	SPOLETO	PUBLIC
735	100	UMBRIA	100805	POLO OSPEDALIERO SPOLETO	VIA LORETO N.3	06049	SPOLETO	PUBLIC

736	100	UMBRIA	100805	POLO OSPEDALIERO SPOLETO	VIA LORETO N.3	06049	SPOLETO	PUBLIC
737	100	UMBRIA	100809	POLO OSPEDALIERO FOLIGNO	VIA ARCAMONE	06034	FOLIGNO	PUBLIC
738	100	UMBRIA	100809	POLO OSPEDALIERO FOLIGNO	VIA ARCAMONE	06034	FOLIGNO	PUBLIC
739	100	UMBRIA	100602	SERVIZIO PSICHIATRICO DIAGNOSI E CURA	VIA T. DI IANNUCCIO,2	05100	TERNI	PUBLIC
740	100	UMBRIA	100806	SANTA MARIA DELLA STELLA	LOCALITA' CICONIA	05019	ORVIETO	PUBLIC
741	100	UMBRIA	100807	PRESIDIO OSPEDALIERO NARNI AMELIA	VIA CAPPUCCINI NUOVI N.3	05035	NARNI	PUBLIC
742	100	UMBRIA	100807	PRESIDIO OSPEDALIERO NARNI AMELIA	VIA CAPPUCCINI NUOVI N.3	05035	NARNI	PUBLIC
743	100	UMBRIA	100807	PRESIDIO OSPEDALIERO NARNI AMELIA	VIA CAPPUCCINI NUOVI N.3	05035	NARNI	PUBLIC
744	100	UMBRIA	100901	AZIENDA OSPEDALIERA DI PERUGIA	VIA BRUNAMONTI 51	06122	PERUGIA	PUBLIC
745	100	UMBRIA	100901	AZIENDA OSPEDALIERA DI PERUGIA	VIA BRUNAMONTI 51	06122	PERUGIA	PUBLIC
746	100	UMBRIA	100902	AZIENDA OSPEDALIERA 'S. MARIA' - TERNI	VIA TRISTANO DI JOANNUCCIO	05100	TERNI	PUBLIC
747	110	MARCHE	110002	OSPEDALE SANTA CROCE FANO	VIA VITTORIO VENETO 2	61032	FANO	PUBLIC
748	110	MARCHE	110003	S. MARIA DELLA MISERICORDIA URBINO	VIA COMANDINO 70	61029	URBINO	PUBLIC
749	110	MARCHE	110004	OSPEDALE LANCIARINI SASSOCORVARO	VIA LANCIARINI	61028	SASSOCORVARO	PUBLIC
750	110	MARCHE	110005	OSPEDALE SS DONNINO E CARLO	VIA G. DI VITTORIO 2	61045	PERGOLA	PUBLIC
751	110	MARCHE	110006	OSPEDALE CIVILE FOSSOMBRONE	VIA KENNEDY	61034	FOSSOMBRONE	PUBLIC
752	110	MARCHE	110007	OSPEDALE CELLI CAGLI	VIA ATANAGI	61043	CAGLI	PUBLIC
753	110	MARCHE	110009	PRESIDIO OSPEDALIERO 'SACRA FAMIGLIA'	VIA XXIV MAGGIO N. 174	61015	NOVAFELTRIA	PUBLIC
754	110	MARCHE	110019	OSPEDALE 'M. MONTESSORI' CHIARAVALLE	VIA ROSSELLI 176	60033	ANCONA	PUBLIC
755	110	MARCHE	110022	U.S.L.N.6 -OSPEDALE CIVILE 'E.PROFILI'-F	VIA CADUTI DEL LAVORO 40	60044	FABRIANO	PUBLIC
756	110	MARCHE	110025	OSPEDALI RIUNITI DI JESI	VIALE DELLA VITTORIA, 76	60035	JESI	PUBLIC
757	110	MARCHE	110026	OSPEDALE 'SANTA CASA' - LORETO	VIA SAN FRANCESCO 1	60025	LORETO	PUBLIC
758	110	MARCHE	110028	OSP.'SS. BENVENUTO E ROCCO' OSIMO	VIA LEOPARDI, 15	60027	OSIMO	PUBLIC
759	110	MARCHE	110031	U.S.L.N.6 -OSP. S.ANTONIO ABATE-SASSOFER	VIA CADUTI DEL LAVORO 40	60041	SASSOFERRATO	PUBLIC
760	110	MARCHE	110032	OSPEDALE SENIGALLIA	VIA CELLINI 1	60019	SENIGALLIA	PUBLIC
761	110	MARCHE	110035	IST.DI RIAB. S.STEFANO - VILLA ADRIA	VIA FLAMINIA 324	60100	ANCONA	PRIVATE
762	110	MARCHE	110036	CASA DI CURA VILLA IGEA	VIA MAGGINI 200	60127	ANCONA	PRIVATE
763	110	MARCHE	110037	CASA DI CURA VILLA SILVIA	VIALE ANITA GARIBALDI 64	60019	SENIGALLIA	PRIVATE
764	110	MARCHE	110038	CASA DI CURA VILLA SERENA	VIA DI COLLE ONORATO 2	60035	JESI	PRIVATE
765	110	MARCHE	110039	CASA DI CURA VILLA JOLANDA	VIA SCISCIANO NORD 11	60030	MAIOLATI SPONTINI	PRIVATE
766	110	MARCHE	110040	OSPEDALE GENERALE PROVINCIALE MACERATA	VIA SANTA LUCIA N.2	62100	MACERATA	PUBLIC
767	110	MARCHE	110041	OSPEDALE S.MARIA DELLA PIETA` - CAMERINO	LOC. CASELLE	62032	CAMERINO	PUBLIC
768	110	MARCHE	110042	OSPEDALE GENERALE DI ZONA - CINGOLI -	VIALE DELA CARITA` N.11	62011	CINGOLI	PUBLIC
769	110	MARCHE	110044	PRES.OSP. OSPEDALE S.SOLLECITO-MATELICA	VIALE EUROPA N 16	62024	MATELICA	PUBLIC
770	110	MARCHE	110045	OSPEDALE CIVILE SANTA LUCIA RECANATI	P.LE ANDREA DA RECANATI 2	62019	RECANATI	PUBLIC
771	110	MARCHE	110046	OSPEDALE 'B.EUSTACCHIO' - S. SEVERINO M.	VIA DEL GLORIOSO N 8	62027	SAN SEVERINO MARCHE	PUBLIC
772	110	MARCHE	110047	OSPEDALE TOLENTINO	VIALE DELLA REPUBBLICA,18	62029	TOLENTINO	PUBLIC
773	110	MARCHE	110048	OSPEDALE TREIA	VIA G. LEOPARDI N. 2	62010	TREIA	PUBLIC
774	110	MARCHE	110049	OSPEDALE GENERALE DI ZONA CIVITANOVA	VIA GINEVRI N,1	62012	CIVITANOVA MARCHE	PUBLIC
775	110	MARCHE	110052	CASA DI CURA DOTT. MARCHETTI SRL	VIA ARIANI, 9 MACERATA	62100	MACERATA	PRIVATE
776	110	MARCHE	110053	CENTRO OSPEDALIERO S.STEFANO	VIA APRUTINA 194 P. POTENZA PICENA	62018	POTENZA PICENA	PRIVATE
777	110	MARCHE	110054	CASA DI CURA VILLA PINI SANATRIX GESTION	VIALE DEI PINI, 31	62012	CIVITANOVA MARCHE	PRIVATE
778	110	MARCHE	110056	OSPEDALE GEN.LE PROV.LE 'C.G.MAZZONI'	VIA DEGLI IRIS MONTICELLI ASCOLI P.	63100	ASCOLI PICENO	PUBLIC
779	110	MARCHE	110058	PRESIDIO OSPEDALIERO FERMO	VIAMURRI 151	63023	FERMO	PUBLIC
780	110	MARCHE	110059	OSPEDALE VITTORIO EMANUELE II AMANDOLA	VIA AGELLO	63021	AMANDOLA	PUBLIC
781	110	MARCHE	110060	PRESIDIO OSPEDALIERO MONTEGIORGIO	VIA DELL` OSPEDALE	63025	MONTEGIORGIO	PUBLIC
782	110	MARCHE	110062	PRESIDIO OSPEDALIERO S.ELPIDIO A MARE	VIA PORTA ROMANA	63023	SANT`ELPIDIO A MARE	PUBLIC
783	110	MARCHE	110064	OSP. 'MADONNA DEL SOCCORSO' S.BENEDETTO	VIA SILVIO PELLICO 68	63039	SAN BENEDETTO DEL TRONTO	PUBLIC
784	110	MARCHE	110070	CASA DI CURA 'VILLA SAN MARCO'	VIA 3 OTTOBRE 11 ASCOLI PICENO	63100	ASCOLI PICENO	PRIVATE
785	110	MARCHE	110071	CASA DI CURA 'SAN GIUSEPPE'	VIA DEI GIRASOLI, 6 MONTICELLI ASCOLI P.	63100	ASCOLI PICENO	PRIVATE
786	110	MARCHE	110072	CASA DI CURA ' VILLA ANNA ' SRL	VIA TOSCANA 159	63039	SAN BENEDETTO DEL TRONTO	PRIVATE
787	110	MARCHE	110073	CASA DI CURA 'STELLA MARIS' SRL	VIA MURRI 1	63039	SAN BENEDETTO DEL TRONTO	PRIVATE
788	110	MARCHE	110074	RITA SRL CASA CURA PRIVATA VILLAVERDE	PIAZZALE KENNEDY2	63023	FERMO	PRIVATE
789	110	MARCHE	110901	AZIENDA OSPEDALIERA SAN SALVATORE	PIAZZALE CINELLI 04	61100	PESARO	PUBLIC
790	110	MARCHE	110905	A.O.U.OSPEDALI RIUNITI - ANCONA	VIA CONCA 71	60100	ANCONA	PUBLIC
791	110	MARCHE	110905	A.O.U.OSPEDALI RIUNITI - ANCONA	VIA CONCA 71	60100	ANCONA	PUBLIC
792	110	MARCHE	110905	A.O.U.OSPEDALI RIUNITI - ANCONA	VIA CONCA 71	60100	ANCONA	PUBLIC
793	110	MARCHE	110921	INRCA ANCONA	VIA DELLA MONTAGNOLA, 81	60100	ANCONA	PUBLIC
794	110	MARCHE	110923	INRCA FERMO	CONTRADA MOSSA 2	63023	FERMO	PUBLIC
795	120	LAZIO	120027	OSPEDALE SAN GIACOMO	VIA ANTONIO CANOVA 29	00186	ROMA	PUBLIC
796	120	LAZIO	120034	ISTITUTO ODONTOIATRIA G.EASTMAN	VIALE REGINA ELENA 287	00161	ROMA	PUBLIC
797	120	LAZIO	120037	OSPEDALE NUOVO REGINA MARGHERITA	VIA MOROSINI 30	00153	ROMA	PUBLIC
798	120	LAZIO	120072	OSPEDALE FATEBENEFRATELLI	ISOLA TIBERINA 39	00186	ROMA	PUBLIC

799	120	LAZIO	120079	CASA DI CURA VILLA DOMELIA S.R.L.	VIA ARBE N.3	00141	ROMA	PRIVATE
800	120	LAZIO	120083	VILLA VALERIA S.R.L.	PIAZZA CARNARO 18	00141	ROMA	PRIVATE
801	120	LAZIO	120084	CASA DI CURA VILLA TIBERIA S.R.L.	VIA EMILIO PRAGA N 26	00137	ROMA	PRIVATE
802	120	LAZIO	120087	CASA DI CURA POLICLINICO ITALIA	PIAZZA DEL CAMPIDANO 6	00162	ROMA	PRIVATE
803	120	LAZIO	120092	CASA DI CURA SANATRIX	VIA TRASONE 61	00199	ROMA	PRIVATE
804	120	LAZIO	120093	CLINICA PARIOLI	VIA FELICE GIORDANO 8	00197	ROMA	PRIVATE
805	120	LAZIO	120098	CASA DI CURA N.SIGNORA DELLA MERCEDE	VIA TAGLIAMENTO 25	00198	ROMA	PRIVATE
806	120	LAZIO	120105	CASA DI CURA MARCO POLO	VIA MARCO POLO 41	00154	ROMA	PRIVATE
807	120	LAZIO	120108	CASA DI CURA ASSUNZIONE DI MARIA SS	VIA NOMENTANA 311	00162	ROMA	PRIVATE
808	120	LAZIO	120114	CASA DI CURA QUISISANA	VIA G GIACOMO PORRO 5	00197	ROMA	PRIVATE
809	120	LAZIO	120119	CASA DI CURA VILLA MAFALDA	VIA MONTE DELLE GIOIE 5	00199	ROMA	PRIVATE
810	120	LAZIO	120120	CASA DI CURA SAN DOMENICO	PIAZZA SASSARI 5	00161	ROMA	PRIVATE
811	120	LAZIO	120129	CASA DI CURA VILLA MARGHERITA	VIALE DI VILLA MASSIMO 48	00161	ROMA	PRIVATE
812	120	LAZIO	120137	CASA DI CURA NOMENTANA	VIA GUATTANI 4	00161	ROMA	PRIVATE
813	120	LAZIO	120141	CASA DI CURA SANTO VOLTO	PIAZZA DEL TEMPIO DI DIANA 12	00153	ROMA	PRIVATE
814	120	LAZIO	120144	CASA DI CURA VALLE GIULIA SPA	VIA GIUSEPPE DE NOTARIS 2/B	00197	ROMA	PRIVATE
815	120	LAZIO	120148	SAN RAFFAELE NOMENTANA	VIA EMILIO PRAGA N 39	00137	ROMA	PRIVATE
816	120	LAZIO	120172	CASA DI CURA MATER DEI	V. ANTONIO BERTOLONI 34	00197	ROMA	PRIVATE
817	120	LAZIO	120243	VILLA SALARIA S.R.L.	VIA F.A.GUALTERIO 127	00139	ROMA	PRIVATE
818	120	LAZIO	120281	CENTRO PER LA SALUTE DELLA DONNA S.ANNA	VIA GARIGLIANO, 55	00198	ROMA	PUBLIC
819	120	LAZIO	120282	VILLA BORGHESE INSTITUTE SrL	VIA SAVERIO MERCADANTE, 18	00198	ROMA	PRIVATE
820	120	LAZIO	120104	CASA DI CURA VILLA FULVIA SRL	VIA APPIA NUOVA 901	00178	ROMA	PRIVATE
821	120	LAZIO	120132	CASA DI CURA GUARNIERI SPA	VIA TOR DE SCHIAVI 139	00172	ROMA	PRIVATE
822	120	LAZIO	120165	POLICLINICO CASILINO	VIA CASILINA 1049	00169	ROMA	PUBLIC
823	120	LAZIO	120166	CASA DI CURA NUOVA ITOR	VIA DI PIETRALATA 162	00158	ROMA	PRIVATE
824	120	LAZIO	120248	CASA DI CURA S.ALESSANDRO SRL	VIA NOMENTANA N[1362 - KM 13,300	00137	ROMA	PRIVATE
825	120	LAZIO	120265	ROME AMERICAN HOSPITAL	VIA EMILIO LONGONI 69	00155	ROMA	PRIVATE
826	120	LAZIO	120267	OSPEDALE SANDRO PERTINI	VIA DEI MONTI TIBURTINI	00157	ROMA	PUBLIC
827	120	LAZIO	120915	POLICL. UNIV. CAMPUS BIO MEDICO	VIA EMILIO LONGONI 69	00155	ROMA	PUBLIC
828	120	LAZIO	120058	OSP. C.T.O. ANDREA ALESINI	VIA SAN NEMESIO 21	00143	ROMA	PUBLIC
829	120	LAZIO	120066	OSPEDALE S. EUGENIO	P.LE DELL`UMANESIMO 10	00144	ROMA	PUBLIC
830	120	LAZIO	120076	MADRE GIUSEPPINA VANNINI	VIA ACQUA BULLICANTE 4	00177	ROMA	PUBLIC
831	120	LAZIO	120085	MADONNA DELLA FIDUCIA	VIA C. CORRENTI N. 6	00179	ROMA	PRIVATE
832	120	LAZIO	120089	-CLINICA NUOVA LATINA	VIA PATRICA 15	00178	ROMA	PRIVATE
833	120	LAZIO	120116	C.D.C. SAN LUCA	VIA TEANO 8/A	00177	ROMA	PRIVATE
834	120	LAZIO	120135	C.D.C. MATER MISERICORDIAE	VIA LATINA 28	00179	ROMA	PRIVATE
835	120	LAZIO	120139	CLINICA LATINA	VIA VULCI 16	00183	ROMA	PRIVATE
836	120	LAZIO	120143	CONCORDIA HOSPITAL	VIA DELLE SETTE CHIESE 90	00145	ROMA	PRIVATE
837	120	LAZIO	120155	C.D.C. ADDOMINALE ALL`EUR	VIALE AFRICA 32	00144	ROMA	PRIVATE
838	120	LAZIO	120157	C.D.C. FABIA MATER	VIA OLEVANO ROMANO 25	00171	ROMA	PRIVATE
839	120	LAZIO	120169	NUOVA CLINICA ANNUNZIATELLA	VIA MEROPIA 124	00147	ROMA	PRIVATE
840	120	LAZIO	120241	C.D.C. VILLA EUROPA ALL`EUR	VIA EUFRATE 27	00144	ROMA	PRIVATE
841	120	LAZIO	120249	C.D.C. VILLA ANNA MARIA	VIA SATURNIA 25	00183	ROMA	PRIVATE
842	120	LAZIO	120909	I.R.C.C.S. S. LUCIA	VIA ARDEATINA 306	00179	ROMA	PUBLIC
843	120	LAZIO	120061	OSPEDALE G. B. GRASSI	VIA G. C. PASSERONI, 30	00122	ROMA	PUBLIC
844	120	LAZIO	120065	CENTRO PARAPLEGICI OSTIA	VIALE VEGA 3	00122	ROMA	PUBLIC
845	120	LAZIO	120075	OSPEDALE ISRAELITICO	VIA FULDA 14	00148	ROMA	PUBLIC
846	120	LAZIO	120103	POLICLINICO DI LIEGRO	VIA DEI BUONVISI 50	00148	ROMA	PUBLIC
847	120	LAZIO	120113	CASA DI CURA VILLA PIA	VIA B RAMAZZINI, 93	00151	ROMA	PRIVATE
848	120	LAZIO	120130	CASA DI CURA VILLA SANDRA	VIA PORTUENSE 798	00148	ROMA	PRIVATE
849	120	LAZIO	120133	CASA DI CURA SALVATOR MUNDI	V.LE DELLE MURA GIANICOLENSI 67	00152	ROMA	PRIVATE
850	120	LAZIO	120138	OSPEDALE SAN GIOVANNI BATTISTA-SMOM	VIA L. E. MORSELLI 13	00148	ROMA	PUBLIC
851	120	LAZIO	120171	CASA DI CURA CITTA` DI ROMA	VIA MAIDALCHINI 20	00152	ROMA	PRIVATE
852	120	LAZIO	120173	EUROPEAN HOSPITAL	VIA PORTUENSE 700	00149	ROMA	PRIVATE
853	120	LAZIO	120191	CASA DI CURA MERRY HOUSE/C.GERIATRICO RO	VIA G. BEDUSCHI 28	00126	ROMA	PRIVATE
854	120	LAZIO	120262	CASA DI CURA VILLA MARIA IMMACOLATA	VIA DEL CASALETTO 391	00151	ROMA	PRIVATE
855	120	LAZIO	120606	CASA DI CURA S. RAFFAELE PORTUENSE	VIA B. RAMAZZINI, 45	00151	ROMA	PRIVATE
856	120	LAZIO	120026	OSPEDALE GENERALE SANTO SPIRITO	LUNGOTEVERE IN SAXIA 3	00193	ROMA	PUBLIC
857	120	LAZIO	120026	OSPEDALE GENERALE SANTO SPIRITO	LUNGOTEVERE IN SAXIA 3	00193	ROMA	PUBLIC
858	120	LAZIO	120030	OSPEDALE REGIONALE OFTALMICO	PIAZZALE DEGLI EROI 11	00136	ROMA	PUBLIC
859	120	LAZIO	120071	OSPEDALE SAN PIETRO FATEBENEFRATELLI	VIA CASSIA 600	00189	ROMA	PUBLIC
860	120	LAZIO	120073	OSPEDALE SAN CARLO DI NANCY	VIA AURELIA 275	00165	ROMA	PUBLIC
861	120	LAZIO	120074	OSP. GEN. DI ZONA 'CRISTO RE'	VIA DELLE CALASANZIANE 25	00167	ROMA	PUBLIC

862	120	LAZIO	120080	CASA DI CURA SAN GIORGIO	VIALE DELLE MEDAGLIE D`ORO 142	00136	ROMA	PRIVATE
863	120	LAZIO	120081	CASA DI CURA E MORELLI	VIA AURELIA 278	00165	ROMA	PRIVATE
864	120	LAZIO	120090	ISTITUTO DI CURA VILLA BENEDETTA	CIRCONVALL CORNELIA 65	00165	ROMA	PRIVATE
865	120	LAZIO	120091	CASA DI CURA VILLA LUISA	VIA SANTA MARIA MEDIATRICE 2	00165	ROMA	PRIVATE
866	120	LAZIO	120097	CASA DI CURA VILLA AURORA	VIA MATTIA BATTISTINI 44	00167	ROMA	PRIVATE
867	120	LAZIO	120115	CASA DI CURA SANTA FAMIGLIA	VIA DEI GRACCHI 134	00192	ROMA	PRIVATE
868	120	LAZIO	120118	CASA DI CURA VILLA DEL ROSARIO	VIA FLAMINIA 499	00191	ROMA	PRIVATE
869	120	LAZIO	120123	CASA DI CURA PRIVATA VILLA STUART	VIA TRIONFALE 5952	00136	ROMA	PRIVATE
870	120	LAZIO	120126	CASA DI CURA SAN GIUSEPPE	VIA BERNARDINO TELESIO 4	00195	ROMA	PRIVATE
871	120	LAZIO	120146	CASA DI CURA S.RITA DA CASCIA	VIA DEGLI SCIPIONI 130	00192	ROMA	PRIVATE
872	120	LAZIO	120150	CASA DI CURA PIO XI	VIA AURELIA 559	00165	ROMA	PRIVATE
873	120	LAZIO	120158	CASA DI CURA PAIDEIA	VIA VINCENZO TIBERIO 46	00191	ROMA	PRIVATE
874	120	LAZIO	120159	CASA DI CURA VILLA CLAUDIA	VIA FLAMINIA NUOVA 280	00191	ROMA	PRIVATE
875	120	LAZIO	120162	CASA DI CURA NS. SIGNORA SACRO CUORE	VIA CARDINAL PACCA 16	00165	ROMA	PRIVATE
876	120	LAZIO	120163	CASA DI CURA SAN FELICIANO	VIA E. DE OSSO, 6	00166	ROMA	PRIVATE
877	120	LAZIO	120179	OSPEDALE ANCELLE DEL BUON PASTORE	VIA DI VALLELUNGA, 8	00166	ROMA	PRIVATE
878	120	LAZIO	120180	AURELIA HOSPITAL	VIA AURELIA 860	00165	ROMA	PRIVATE
879	120	LAZIO	120254	ARS MEDICA SPA	VIA CESARE FERRERO DI CAMBIANO 29	00191	ROMA	PRIVATE
880	120	LAZIO	120256	CASA DI CURA CLINICA VILLA FLAMINIA	VIA LUIGI BODIO, 58	00191	ROMA	PRIVATE
881	120	LAZIO	120257	CASA DI CURA VILLA S. MARIA DI LEUCA	VIA TIBERINA 173	00188	ROMA	PRIVATE
882	120	LAZIO	120264	CASA DI CURA VILLA VERDE	VIA DI TORREVECCHIA 250	00168	ROMA	PRIVATE
883	120	LAZIO	120272	VILLA SACRA FAMIGLIA	LARGO OTTORINO RESPIGHI 6	00135	ROMA	PRIVATE
884	120	LAZIO	120328	CASA DI CURA PRIVATA `DON CARLO GNOCCHI`	VIA MARESCIALLO CAVIGLIA 30	00194	ROMA	PRIVATE
885	120	LAZIO	120911	I.D.I.	VIA MONTI DI CRETA 104	00146	ROMA	PUBLIC
886	120	LAZIO	120912	INRCA	VIA CASSIA 1167	00189	ROMA	PUBLIC
887	120	LAZIO	120045	OSPEDALE SAN PAOLO	LARGO DONATORI DEL SANGUE	00053	CIVITAVECCHIA	PUBLIC
888	120	LAZIO	120059	OSPEDALE PADRE PIO DI BRACCIANO	VIA DELLE COSTE	00062	BRACCIANO	PUBLIC
889	120	LAZIO	120140	CASA DI CURA SILIGATO	VIA BUONARROTI, 54	00053	CIVITAVECCHIA	PRIVATE
890	120	LAZIO	120197	SANTO VOLTO	VIA C. BATTISTI, 7	00058	SANTA MARINELLA	PRIVATE
891	120	LAZIO	120046	OSPEDALE L.PARODI DELFINO	PIAZZA ALDO MORO N. 1	00034	COLLEFERRO	PUBLIC
892	120	LAZIO	120049	0SPEDALE SS. GONFALONE	VIA ROBERTO FARAVELLI 27	00015	MONTEROTONDO	PUBLIC
893	120	LAZIO	120051	0SPEDALE CIVILE CONIUGI BERNARDINI	VIA PIO XII	00036	PALESTRINA	PUBLIC
894	120	LAZIO	120052	OSPEDALE A. ANGELUCCI	C.DA COLLE CISTERNA	00028	SUBIACO	PUBLIC
895	120	LAZIO	120053	OSPEDALE SAN GIOVANNI EVANGELISTA	VIA PARROZZANI N. 3	00019	TIVOLI	PUBLIC
896	120	LAZIO	120057	OSPEDALE SAN GIOVANNI BATTISTA	PIAZZA MASSIMO D`AZEGLIO	00039	ZAGAROLO	PUBLIC
897	120	LAZIO	120062	OSPEDALE SS. SALVATORE	VIALE POSSENTI SNC	00018	PALOMBARA SABINA	PUBLIC
898	120	LAZIO	120088	I.N.I. Srl Divisione Medicus Hotel	PIAZZALE S. GIOVANNI DI DIO- MONTERIPO	00019	TIVOLI	PRIVATE
899	120	LAZIO	120186	NOMENTANA HOSPITAL	VIALE N. BERLOCO, 60 - FONTE NUOVA	00010	FONTE NUOVA	PRIVATE
900	120	LAZIO	120199	I.N.I. Srl Divisione Villa Dante	VIA ROMA N. 298	00012	GUIDONIA MONTECELIO	PRIVATE
901	120	LAZIO	120273	CASA DI CURA VILLA LUANA	via tivoli 66	00010	POLI	PRIVATE
902	120	LAZIO	120036	OSP. VILLA ALBANI ANZIO	VIA ALDOBRANDINI 32	00042	ANZIO	PUBLIC
903	120	LAZIO	120043	OSP. ANZIO-NETTUNO	VIA CUPA DEI MARMI ANZIO	00042	ANZIO	PUBLIC
904	120	LAZIO	120044	OSP.RIUNITI ALBANO-GENZANO	VIA OLIVELLA	00041	ALBANO LAZIALE	PUBLIC
905	120	LAZIO	120044	OSP.RIUNITI ALBANO-GENZANO	VIA OLIVELLA	00041	ALBANO LAZIALE	PUBLIC
906	120	LAZIO	120047	OSP. S.SEBASTIANO FRASCATI	VIA TUSCOLANA	00044	FRASCATI	PUBLIC
907	120	LAZIO	120048	OSP. S.GIUSEPPE MARINO	VIALE XXIV MAGGIO	00047	MARINO	PUBLIC
908	120	LAZIO	120054	OSP.'PAOLO COLOMBO' VELLETRI	VIA ORTI GINNETTI 6	00049	VELLETRI	PUBLIC
909	120	LAZIO	120055	OSP. ARICCIA	VIALE A.GHIGI 64	00040	ARICCIA	PUBLIC
910	120	LAZIO	120064	OSP. CARTONI ROCCA PRIORA	VIA MALPASSO D`ACQUA 2	00040	ROCCA PRIORA	PUBLIC
911	120	LAZIO	120070	OSP. REGINA APOSTOLORUM ALBANO	VIA S.FRANCESCO 50	00041	ALBANO LAZIALE	PUBLIC
912	120	LAZIO	120082	VILLA DELLE QUERCIE-POLIGEST	VIA DELLE VIGNE N[19 NEMI	00040	NEMI	PRIVATE
913	120	LAZIO	120096	C.D.C. MADONNA DELLE GRAZIE	VIALE SALVO D,ACQUISTO 67	00049	VELLETRI	PRIVATE
914	120	LAZIO	120122	C.D.C. SAN RAFFAELE ROCCA DI PAPA	VIA ARICCIA 16	00040	ROCCA DI PAPA	PRIVATE
915	120	LAZIO	120131	C.D.C. VILLA DEI PINI-MERINVEST	VIA CASAL DI BROCCO 19	00042	ANZIO	PRIVATE
916	120	LAZIO	120134	CASA DI CURA S.ANNA POMEZIA	VIA DEL MARE 69-71	00040	POMEZIA	PRIVATE
917	120	LAZIO	120149	C.D.C. CLINICA VILLA NINA SRL	VIA NETTUNENSE,110	00047	MARINO	PRIVATE
918	120	LAZIO	120176	C.D.C. I.N.I. SRL	VIA S.ANNA SNC	00046	GROTTAFERRATA	PRIVATE
919	120	LAZIO	120196	C.D.C. SAN RAFFAELE_VELLETRI.	VIA DEI LAGHI KM 19,600	00049	VELLETRI	PRIVATE
920	120	LAZIO	120280	C.D.C. SAN RAFFAELE MONTECOMPATRI	VIA SAN SILVESTRO 21/25	00040	MONTECOMPATRI	PRIVATE
921	120	LAZIO	120002	OSPEDALE DI ACQUAPENDENTE	VIA C. BATTISTI	01021	ACQUAPENDENTE	PUBLIC
922	120	LAZIO	120003	OSPEDALE DI CIVITACASTELLANA	VIA FERRETTI	01033	CIVITA CASTELLANA	PUBLIC
923	120	LAZIO	120004	OSPEDALE DI MONTEFIASCONE	VIA VERENTANA	01027	MONTEFIASCONE	PUBLIC
924	120	LAZIO	120006	OSPEDALE DI RONCIGLIONE	VIA OSPEDALE DI S.ANNA,17	01037	RONCIGLIONE	PUBLIC

925	120	LAZIO	120007	OSPEDALE DI TARQUINIA	VIALE IGEA,1	01016	TARQUINIA	PUBLIC
926	120	LAZIO	120012	CASA DI CURA SALUS	VIALE TRIESTE	01100	VITERBO	PRIVATE
927	120	LAZIO	120013	CENTRO DI RIABILITAZIONE DI NEPI	VIA CASSIA KM 37	01036	NEPI	PRIVATE
928	120	LAZIO	120014	CASA DI CURA S.TERESA DEL BAMBIN GESU`	VIALE TRIESTE,87	01100	VITERBO	PRIVATE
929	120	LAZIO	120015	VILLA IMMACOLATA	STRADA SAMMARTINESE	01100	VITERBO	PRIVATE
930	120	LAZIO	120271	OSPEDALE DI BELCOLLE	STRADA SAMMARTINESE	01100	VITERBO	PUBLIC
931	120	LAZIO	120914	ISTITUTO VILLA PAOLA	VIA P. MONTI,1	01012	CAPRANICA	PUBLIC
932	120	LAZIO	120019	OSPEDALE S.CAMILLO DE LELLIS	VIALE KENNEDY	02100	RIETI	PUBLIC
933	120	LAZIO	120020	OSPEDALE MARZIO MARINI	LOCALITA` FILONI	02046	MAGLIANO SABINA	PUBLIC
934	120	LAZIO	120022	OSPEDALE FRANCESCO GRIFONI AMATRICE	VIALE FRANCESCO GRIFONI N.30	02012	AMATRICE	PUBLIC
935	120	LAZIO	120200	PRESIDIO OSPEDALIERO NORD	VIA CANOVA	04100	LATINA	PUBLIC
936	120	LAZIO	120200	PRESIDIO OSPEDALIERO NORD	VIA CANOVA	04100	LATINA	PUBLIC
937	120	LAZIO	120200	PRESIDIO OSPEDALIERO NORD	VIA CANOVA	04100	LATINA	PUBLIC
938	120	LAZIO	120204	PRESIDIO OSPEDALIERO CENTRO	VIA FIRENZE	04019	TERRACINA	PUBLIC
939	120	LAZIO	120204	PRESIDIO OSPEDALIERO CENTRO	VIA FIRENZE	04019	TERRACINA	PUBLIC
940	120	LAZIO	120206	PRESIDIO OSPEDALIERO SUD	VIA APPIA LATO NAPOLI	04023	FORMIA	PUBLIC
941	120	LAZIO	120206	PRESIDIO OSPEDALIERO SUD	VIA APPIA LATO NAPOLI	04023	FORMIA	PUBLIC
942	120	LAZIO	120206	PRESIDIO OSPEDALIERO SUD	VIA APPIA LATO NAPOLI	04023	FORMIA	PUBLIC
943	120	LAZIO	120209	CASA DEL SOLE CLINICA TOMMASO COSTA	VIA G.PAONE 58	04023	FORMIA	PRIVATE
944	120	LAZIO	120211	CASA DI CURA VILLA AZZURRA SRL TERRACINA	LUNGOMARE MATTEOTTI 4 TERRACINA	04019	TERRACINA	PRIVATE
945	120	LAZIO	120212	ISTIT CHIR ORTOP TRAUMATOLOGICO	VIA FAGGIANA	04100	LATINA	PRIVATE
946	120	LAZIO	120213	CASA DI CURA SAN MARCO	VIALE XXI APRILE	04100	LATINA	PRIVATE
947	120	LAZIO	120214	ISTITUTO FISIOTERAPICO C.FRANCESCHINI	LOCALITA` SELVAPIANA 2	04016	SABAUDIA	PRIVATE
948	120	LAZIO	120215	CASA DI CURA CITTA` DI APRILIA	VIA DELLE PALME 25	04011	APRILIA	PRIVATE
949	120	LAZIO	120278	VILLA SILVANA	VIALE EUROPA1/3	04011	APRILIA	PRIVATE
950	120	LAZIO	120216	OSPEDALE UMBERTO I[FROSINONE	VIALE MAZZINI	03100	FROSINONE	PUBLIC
951	120	LAZIO	120217	OSPEDALE SAN BENEDETTO ALATRI	LOCALITA` CHIAPPITTO	03011	ALATRI	PUBLIC
952	120	LAZIO	120218	OSPEDALE CIVILE ANAGNI	VIA D. CAPO 2	03012	ANAGNI	PUBLIC
953	120	LAZIO	120219	OSPEDALE CIVILE SANTA CROCE ARPINO	VIA VITTORIO COLONNA	03033	ARPINO	PUBLIC
954	120	LAZIO	120220	OSPEDALE CIVILE DELLA CROCE ATINA	VIA VITTORIO EMANUELE	03042	ATINA	PUBLIC
955	120	LAZIO	120221	OSPEDALE CIVILE CECCANO	BORGO SANTA LUCIA - 52	03023	CECCANO	PUBLIC
956	120	LAZIO	120222	HOSP. RIABILITITATIVO "FERRARI" CEPRANO	VIA REGINA MARGHERITA 5	03024	CEPRANO	PUBLIC
957	120	LAZIO	120223	HOSP. RIABILITATIVO CIVICO FERENTINO	PL. DELL`OSPIZIO	03013	FERENTINO	PUBLIC
958	120	LAZIO	120224	OSP. CIVILE IN MEM.DEI CADUTI ISOLA LIRI	VIA OSPEDALE	03036	ISOLA DEL LIRI	PUBLIC
959	120	LAZIO	120225	OSPEDALE CIVILE PAS. D. PRETE PONTECORVO	VIA SAN GIOVANNI BATTISTA	03037	PONTECORVO	PUBLIC
960	120	LAZIO	120226	OSPEDALE CIVILE S.S.TRINITA` N.SEDE SORA	LOCALITA` SAN MARCIANO	03039	SORA	PUBLIC
961	120	LAZIO	120228	OSPEDALE CIVILE G. DE BOSIS CASSINO	VIA CASILINA NORD	03043	CASSINO	PUBLIC
962	120	LAZIO	120230	CASA DI CURA PRIV. S. ANNA S.R.L CASSINO	VIA K. HEROLD	03043	CASSINO	PRIVATE
963	120	LAZIO	120234	CASA DI CURA PRIV. VILLA GIOIA SORA	VIAALE S. DOMENICO 1/F	03039	SORA	PRIVATE
964	120	LAZIO	120235	CASA DI CURA PRIV. VILLA SERENA CASSINO	CORSO DELLA REPUBBLICA 204	03043	CASSINO	PRIVATE
965	120	LAZIO	120236	CASA DI CURA PRIV. SANTA TERESA ISOLA L.	VIA NAZIONALE 7/25	03036	ISOLA DEL LIRI	PRIVATE
966	120	LAZIO	120277	EX CENTER HOSPITAL'SAN RAFFAELE' CASSINO	VIA G. DI BIASIO 218	03043	CASSINO	PRIVATE
967	120	LAZIO	120279	I.N.I. DIV.DISTACCAT CITTA` BIANCA	VIA FOIANO N.4	03020	VEROLI	PRIVATE
968	120	LAZIO	120901	AZ.OSP.SAN CAMILLO-FORLANINI	PIAZZA CARLO FORLANINI, 1	00151	ROMA	PUBLIC
969	120	LAZIO	120902	AZIENDA OSP. S.GIOVANNI/ADDOLORATA ROMA	VIA DELL`AMBA ARADAM 9	00184	ROMA	PUBLIC
970	120	LAZIO	120903	AZ. COMPL. OSP. S.FILIPPO NERI	VIA MARTINOTTI N. 20	00135	ROMA	PUBLIC
971	120	LAZIO	120903	AZ. COMPL. OSP. S.FILIPPO NERI	VIA MARTINOTTI N. 20	00135	ROMA	PUBLIC
972	120	LAZIO	120903	AZ. COMPL. OSP. S.FILIPPO NERI	VIA MARTINOTTI N. 20	00135	ROMA	PUBLIC
973	120	LAZIO	120903	AZ. COMPL. OSP. S.FILIPPO NERI	VIA MARTINOTTI N. 20	00135	ROMA	PUBLIC
974	120	LAZIO	120904	OSPEDALE PEDIATRICO BAMBINO GESU`	PIAZZA S.ONOFRIO 4	00165	ROMA	PUBLIC
975	120	LAZIO	120904	OSPEDALE PEDIATRICO BAMBINO GESU`	PIAZZA S.ONOFRIO 4	00165	ROMA	PUBLIC
976	120	LAZIO	120904	OSPEDALE PEDIATRICO BAMBINO GESU`	PIAZZA S.ONOFRIO 4	00165	ROMA	PUBLIC
977	120	LAZIO	120905	POLICLINICO A. GEMELLI E C.I.C.	LARGO AGOSTINO GEMELLI 8	00168	ROMA	PUBLIC
978	120	LAZIO	120905	POLICLINICO A. GEMELLI E C.I.C.	LARGO AGOSTINO GEMELLI 8	00168	ROMA	PUBLIC
979	120	LAZIO	120906	POLICLINICO U. I	VIALE DEL POLICLINICO 155	00161	ROMA	PUBLIC
980	120	LAZIO	120908	ISTITUTI FISIOTERAPICI OSPITALIERI	VIA ELIO CHIANESI 53	00128	ROMA	PUBLIC
981	120	LAZIO	120908	ISTITUTI FISIOTERAPICI OSPITALIERI	VIA ELIO CHIANESI 53	00128	ROMA	PUBLIC
982	120	LAZIO	120910	IRCCS SAN RAFFAELE PISANA	VIA DELLA PISANA 235	00163	ROMA	PUBLIC
983	120	LAZIO	120918	INMI 'L.SPALLANZANI' - IRCCS	VIA PORTUENSE,292	00149	ROMA	PUBLIC
984	120	LAZIO	120919	AZIENDA OSPEDALIERA SANT`ANDREA	VIA DI GROTTAROSSA 1035 - 1039	00189	ROMA	PUBLIC
985	120	LAZIO	120920	AZ. OSP. UNIV. POLICLINICO TOR VERGATA	VIALE OXFORD , 81	00133	ROMA	PUBLIC
986	130	ABRUZZO	130002	PO 'DELL` ANNUNZIATA' SULMONA	VIALE MAZZINI	67039	SULMONA	PUBLIC
987	130	ABRUZZO	130003	PO 'S.NICOLA E FILIPPO' AVEZZANO	VIA G. DI VITTORIO	67051	AVEZZANO	PUBLIC

988	130	ABRUZZO	130004	PO S. RINALDI PESCINA	VIA S. RINALDI	66057	PESCINA	PUBLIC
989	130	ABRUZZO	130005	PO CASTEL DI SANGRO	VIA LA CROCE	67031	CASTEL DI SANGRO	PUBLIC
990	130	ABRUZZO	130006	PO UMBERTO 1° TAGLIACOZZO	VIA VARIANTE TIBURTINA	67069	TAGLIACOZZO	PUBLIC
991	130	ABRUZZO	130010	CASA DI CURA 'N. DI LORENZO'	VIA VENETO,37	67051	AVEZZANO	PRIVATE
992	130	ABRUZZO	130011	CASA DI CURA S. MARIA	VIA TRIESTE,46	67051	AVEZZANO	PRIVATE
993	130	ABRUZZO	130012	CASA DI CURA L`IMMACOLATA	VIA COLLUTRI,6	67043	CELANO	PRIVATE
994	130	ABRUZZO	130013	CDC S. RAFFAELE	VIALE DELL`AGRICOLTURA	67039	SULMONA	PRIVATE
995	130	ABRUZZO	130039	INI CANISTRO SRL	LOCALITA` COTARDO	67050	CANISTRO	PRIVATE
996	130	ABRUZZO	130041	NOVA SALUS SRL	VIA ROMA 75/A	67059	TRASACCO	PRIVATE
997	130	ABRUZZO	130026	Pres. Osp. Clinicizzato SS. ANNUNZIATA	VIA DEI VESTINI	66100	CHIETI	PUBLIC
998	130	ABRUZZO	130031	OSPEDALE MARIA SS IMMACOLATA	VIA ANELLO 6	66016	GUARDIAGRELE	PUBLIC
999	130	ABRUZZO	130032	OSPEDALE 'G. BERNABEO' - ORTONA	CONTRADA S. LIBERATA	66026	ORTONA	PUBLIC
1000	130	ABRUZZO	130034	VILLA PINI D`ABRUZZO S.r.l.	VIA DEI FRENTANI, 228	66100	CHIETI	PRIVATE
1001	130	ABRUZZO	130035	CASA DI CURA DOTT SPATOCCO	VIA AMENDOLA 93	66100	CHIETI	PRIVATE
1002	130	ABRUZZO	130028	OSPEDALE RENZETTI LANCIANO	VIA DEL MARE 1	66034	LANCIANO	PUBLIC
1003	130	ABRUZZO	130029	OSPEDALE CIVILE VASTO	VIA C. DE LELLIS, 1	66054	VASTO	PUBLIC
1004	130	ABRUZZO	130030	OSPEDALE VITT.EMANUELE ATESSA	VIA IANICO	66041	ATESSA	PUBLIC
1005	130	ABRUZZO	130033	OSPEDALE CIVILE CONSALVI	VIA AVENTINO	66040	CASOLI	PUBLIC
1006	130	ABRUZZO	130037	OSPEDALE CIVILE GISSI	VIA ITALIA	66052	GISSI	PUBLIC
1007	130	ABRUZZO	130042	ISTITUTO SAN FRANCESCO - VASTO-	VIALE DALMAZIA, 116	66054	VASTO	PRIVATE
1008	130	ABRUZZO	130001	OSPEDALE CIVILE S.SALVATORE	VIA VETOIO - COPPITO	67010	L`AQUILA	PUBLIC
1009	130	ABRUZZO	130008	SANATRIX S.R.L.	VIA XXIV MAGGIO 7	67100	L`AQUILA	PRIVATE
1010	130	ABRUZZO	130040	CASA DI CURA PRIVATA VILLA LETIZIA	S.S. 80 N. 25/B - PRETURO	67010	L`AQUILA	PRIVATE
1011	130	ABRUZZO	130018	OSPEDALE CIVILE SPIRITO SANTO	VIA R PAOLINI N 45	65124	PESCARA	PUBLIC
1012	130	ABRUZZO	130019	OSPEDALE CIVILE S.MASSIMO	VIA BGT.ALPINI,1 PENNE PE	65017	PENNE	PUBLIC
1013	130	ABRUZZO	130020	OSPEDALE CIVILE SS.TRINITA`	VIA BERLINGUER 118	65026	POPOLI	PUBLIC
1014	130	ABRUZZO	130022	CASA DI CURA PRIVATA 'PIERANGELI S.R.L.'	PIAZZA L.PIERANGELI, N.1	65124	PESCARA	PRIVATE
1015	130	ABRUZZO	130024	C.DI CURA PRIV.VILLA SERENA DR.PETRUZZI	VIALE L.PETRUZZI, N.19	65013	CITTA` SANT`ANGELO	PRIVATE
1016	130	ABRUZZO	130038	PRES. OSP. S. VALENTINO	VIA PER CARAMANICO	65020	SAN VALENTINO IN ABRUZZO CITERIORE	PUBLIC
1017	130	ABRUZZO	130014	OSPEDALE MAZZINI	PIAZZA ITALIA	64100	TERAMO	PUBLIC
1018	130	ABRUZZO	130015	MARIA SS. DELLO SPLENDORE	VIA GRAMSCI	64021	GIULIANOVA	PUBLIC
1019	130	ABRUZZO	130016	OSPEDALE CIVILE S. LIBERATORE	PIAZZALE ALESSANDRINI	64032	ATRI	PUBLIC
1020	130	ABRUZZO	130017	OSPEDALE VAL VIBRATA	VIA ALLA SALARA	64027	SANT`OMERO	PUBLIC
1021	140	MOLISE	140001	PRESIDIO OSPEDALIERO 'A.CARDARELLI'	C.DA TAPPINO	80100	CAMPOBASSO	PUBLIC
1022	140	MOLISE	140002	Ospedale ' G. Vietri' Larino	Via Lualdi	86035	LARINO	PUBLIC
1023	140	MOLISE	140003	Ospedale 'S. Timoteo' Termoli	Viale Padre Pio	86039	TERMOLI	PUBLIC
1024	140	MOLISE	140004	CASA DI CURA VILLA MARIA SRL	VIA PRINCIPE DI PIEMONTE 4	86100	CAMPOBASSO	PRIVATE
1025	140	MOLISE	140005	CASA DI CURA VILLA ESTHER DI FORTE ELISA	VIA GINO DI BIASE, 18	86021	BOJANO	PRIVATE
1026	140	MOLISE	140006	OSPEDALE FERDINANDO VENEZIALE	VIA S. IPPOLITO	86170	ISERNIA	PUBLIC
1027	140	MOLISE	140006	OSPEDALE FERDINANDO VENEZIALE	VIA S. IPPOLITO	86170	ISERNIA	PUBLIC
1028	140	MOLISE	140007	PRESIDIO OSPEDALIERO S. FRANCESCO CARACC	VIA MARCONI,22	86081	AGNONE	PUBLIC
1029	140	MOLISE	140009	CENTRO DI RICERCA SCIENZE BIOMED U.C.S.C	L.GO AGOSTINO GEMELLI, 1	86100	CAMPOBASSO	PUBLIC
1030	140	MOLISE	140010	ISTITUTO EUROPEO di RIABILITAZIONE S.r.l	VIA ACQUA SOLFUREA N°1	86170	ISERNIA	PRIVATE
1031	140	MOLISE	140911	NEUROMED I.R.C.C.S.	VIA ATINENSE N°18	94038	POZZILLI	PUBLIC
1032	150	CAMPANIA	150140	OSPEDALE ARIANO IRPINO	CSO VITT EMANUELE	83031	ARIANO IRPINO	PUBLIC
1033	150	CAMPANIA	150141	OSPEDALE DI BISACCIA	PIANO REGOLATORE	83044	BISACCIA	PUBLIC
1034	150	CAMPANIA	150146	CASA DI CURA VILLA MARIA	PASSO DI MIRABELLA ECLANO VIA POZZIL	83030	MIRABELLA ECLANO	PRIVATE
1035	150	CAMPANIA	150150	OSPEDALE S.ANGELO DEI LOMBARDI	LOCALITA` QUADRIVIO	83054	SANT`ANGELO DEI LOMBARDI	PUBLIC
1036	150	CAMPANIA	150150	OSPEDALE S.ANGELO DEI LOMBARDI	LOCALITA` QUADRIVIO	83054	SANT`ANGELO DEI LOMBARDI	PUBLIC
1037	150	CAMPANIA	150139	OSPEDALE LANDOLFI SOLOFRA	VIA MELITO	83029	SOLOFRA	PUBLIC
1038	150	CAMPANIA	150143	CASA DI CURA VILLA ESTER	VIA S.TOMMASO 33	83100	AVELLINO	PRIVATE
1039	150	CAMPANIA	150144	CASA DI CURA VILLA MARIA	VIA NAZIONALE	83022	BAIANO	PRIVATE
1040	150	CAMPANIA	150145	CASA DI CURA S.RITA	VIA APPIA	83042	ATRIPALDA	PRIVATE
1041	150	CAMPANIA	150147	CASA DI CURA VILLA DEI PINI	VIA PENNINI	83100	AVELLINO	PRIVATE
1042	150	CAMPANIA	150148	CASA DI CURA MONTEVERGINE	VIA SAN MODESTINO , 8	83013	MERCOGLIANO	PRIVATE
1043	150	CAMPANIA	150149	CASA DI CURA VILLA DEI PLATANI	VIA ERRICO	83100	AVELLINO	PRIVATE
1044	150	CAMPANIA	150031	OSPEDALE SAN GIOVANNI DI DIO	VIA CAUDINA - 5	82019	SANT`AGATA DE` GOTI	PUBLIC
1045	150	CAMPANIA	150032	OSP.SACRO CUORE DI GESU`FATEBENEFRATELLI	VIALE PRINCIPE DI NAPOLI 14/A	82100	BENEVENTO	PUBLIC
1046	150	CAMPANIA	150033	CASA DI CURA GE.P.O.S. SRL	VIA ROMA 29	82037	TELESE	PRIVATE
1047	150	CAMPANIA	150034	CASA DI CURA NUOVA CLINICA S.RITA	VIALE MELLUSI,103	82100	BENEVENTO	PRIVATE
1048	150	CAMPANIA	150035	CASA DI CURA SAN FRANCESCO	VIALE EUROPA 21	82037	TELESE	PRIVATE
1049	150	CAMPANIA	150036	OSPEDALE SS.MARIA DELLE GRAZIE	VIA CESINE DI SOPRA	82032	CERRETO SANNITA	PUBLIC
1050	150	CAMPANIA	150036	OSPEDALE SS.MARIA DELLE GRAZIE	VIA CESINE DI SOPRA	82032	CERRETO SANNITA	PUBLIC

1051	150	CAMPANIA	150350	C.M.R. S.P.A.- CENTRO MED.DIAGN.E RIAB.	VIA PENNINO TRAV.MUSTILLI	82019	SANT`AGATA DE` GOTI	PRIVATE
1052	150	CAMPANIA	150371	CASA DI CURA VILLA MARGHERITA SRL	CONTRADA PIANO CAPPELLE	82100	BENEVENTO	PRIVATE
1053	150	CAMPANIA	150911	FONDAZIONE 'MAUGERI' IRCCS TELESE TERME	VIA BAGNI VECCHI,1- TELESE -Terme	82037	TELESE	PUBLIC
1054	150	CAMPANIA	150014	CLINICA SANT`ANNA	VIA ROMA 124	81100	CASERTA	PRIVATE
1055	150	CAMPANIA	150015	CLINICA SAN LUCA	VIA TESCIONE 20	81100	CASERTA	PRIVATE
1056	150	CAMPANIA	150016	VILLA DEL SOLE	VIA NAZ.APPIA	81100	CASERTA	PRIVATE
1057	150	CAMPANIA	150020	CLINICA SAN MICHELE	VIA APPIA 176	81024	MADDALONI	PRIVATE
1058	150	CAMPANIA	150023	VILLA DEI PINI	VIA MATESE, 42	81016	PIEDIMONTE MATESE	PRIVATE
1059	150	CAMPANIA	150025	VILLA DEGLI ULIVI	VIA SANNITICA SAN LEUCIO	81100	CASERTA	PRIVATE
1060	150	CAMPANIA	150028	VILLA DELLE MAGNOLIE	VIA CIUMMIENTO -CASTELMORRONE	81020	CASTEL MORRONE	PRIVATE
1061	150	CAMPANIA	150413	DEA MADDALONI-MARCIANISE-S.FELICE A CANC	VIA LIBERTA'	81024	MADDALONI	PUBLIC
1062	150	CAMPANIA	150413	DEA MADDALONI-MARCIANISE-S.FELICE A CANC	VIA LIBERTA'	81024	MADDALONI	PUBLIC
1063	150	CAMPANIA	150413	DEA MADDALONI-MARCIANISE-S.FELICE A CANC	VIA LIBERTA'	81024	MADDALONI	PUBLIC
1064	150	CAMPANIA	150414	DEA PIEDIMONTE MATESE-TEANO	VIA MATESE	81016	PIEDIMONTE MATESE	PUBLIC
1065	150	CAMPANIA	150414	DEA PIEDIMONTE MATESE-TEANO	VIA MATESE	81016	PIEDIMONTE MATESE	PUBLIC
1066	150	CAMPANIA	150004	OSPEDALE SAN GIUSEPPE E MELORIO	VIA MELORIO	81055	SANTA MARIA CAPUA VETERE	PUBLIC
1067	150	CAMPANIA	150005	PRES.OSPEDALIERO 'F.PALASCIANO' CAPUA	VIA F.PALASCIANO	81043	CAPUA	PUBLIC
1068	150	CAMPANIA	150010	PRESIDIO OSPEDALIERO 'SAN ROCCO'	VIA SESSA MIGNANO	81037	SESSA AURUNCA	PUBLIC
1069	150	CAMPANIA	150013	PRESIDIO OSPEDALIERO S.G.MOSCATI AVERSA	VIA GRAMSCI	81031	AVERSA	PUBLIC
1070	150	CAMPANIA	150017	CASA DI CURA VILLA FIORITA	VIA F.SAPORITO 24 AVERSA	81031	AVERSA	PRIVATE
1071	150	CAMPANIA	150018	CASA DI CURA SAN PAOLO	VIA VITO DI IASI 29 AVERSA	81031	AVERSA	PRIVATE
1072	150	CAMPANIA	150019	CASA DI CURA 'VILLA FIORITA' SPA	VIA APPIA km 199	81043	CAPUA	PRIVATE
1073	150	CAMPANIA	150021	CASA DI CURA PINETA GRANDE	VIA DOMITIANA KM 30	81030	CASTEL VOLTURNO	PRIVATE
1074	150	CAMPANIA	150022	MINERVA S.P.A. SANTA MARIA DELLA SALUTE	VIA AVEZZANA 53/55	81005	SANTA MARIA CAPUA VETERE	PRIVATE
1075	150	CAMPANIA	150024	CASA DI CURA VILLA ORTENSIA	VIA PONTE VECCHIO ROMANO 6	81043	CAPUA	PRIVATE
1076	150	CAMPANIA	150073	CASA DI CURA OSPEDALE FATEBENEFRATELLI	VILLA B. CONSIGLIO VIA MANZONI 220	80123	NAPOLI	PUBLIC
1077	150	CAMPANIA	150075	CASA DI CURA OSP. INTERNAZIONALE	VIA TASSO 38	80122	NAPOLI	PRIVATE
1078	150	CAMPANIA	150076	CASA DI CURA VILLALBA	VIA PROVINCIALE S.GENNARO 86	80125	NAPOLI	PRIVATE
1079	150	CAMPANIA	150078	CASA DI CURA VILLA CAMALDOLI	VIALE PRIV.VILLA CAMALDOLI,55	80131	NAPOLI	PRIVATE
1080	150	CAMPANIA	150079	CASA DI CURA VILLA ANGELA	VIA MICHELANGELO SCHIPA 40	80122	NAPOLI	PRIVATE
1081	150	CAMPANIA	150081	CASA DI CURA CLINIC CENTER	VIA CINZIA PARCO SAN PAOLO	80126	NAPOLI	PRIVATE
1082	150	CAMPANIA	150083	CASA DI CURA VILLA DEL SOLE	VIA MANZONI 15	80123	NAPOLI	PRIVATE
1083	150	CAMPANIA	150086	CASA DI CURA 'VILLA RUSSO' S.P.A.	VIA MIANO N 94	80145	NAPOLI	PRIVATE
1084	150	CAMPANIA	150088	CASA DI CURA COLUCCI	VIA cupa delle tozzole,2	80131	NAPOLI	PRIVATE
1085	150	CAMPANIA	150092	CASA DI CURA VILLA DELLE QUERCE	VIA BATTISTELLO CARACCIOLO 48	80136	NAPOLI	PRIVATE
1086	150	CAMPANIA	150093	CASA DI CURA RUESH	VI/LE MARIA CRISTINA DI SAVOIA,39	80122	NAPOLI	PRIVATE
1087	150	CAMPANIA	150094	CASA DI CURA VESUVIO	VIA L. VOLPICELLA 493	80147	NAPOLI	PRIVATE
1088	150	CAMPANIA	150095	CASA DI CURA MEDITERRANEA	VIA ORAZIO 2	80136	NAPOLI	PRIVATE
1089	150	CAMPANIA	150099	CASA DI CURA SANTA PATRIZIA	C. SECONDIGLIANO 7 BIS	80144	NAPOLI	PRIVATE
1090	150	CAMPANIA	150104	CASA DI CURA VILLA CINZIA	VIA EPOMEO 74	80126	NAPOLI	PRIVATE
1091	150	CAMPANIA	150106	CASA DI CURA TASSO	VIA BERNARDO CAVALLINO,102	80127	NAPOLI	PRIVATE
1092	150	CAMPANIA	150109	CLINICA SANATRIX SPA	VIA S.DOMENICO 31	80127	NAPOLI	PRIVATE
1093	150	CAMPANIA	150120	CASA DI CURA SANTO STEFANO	VL MICHELANGELO DA CARAVAGGIO 2	80126	NAPOLI	PRIVATE
1094	150	CAMPANIA	150196	FONDAZIONE EVANGELICA BETANIA	VIA ARGINE	80147	NAPOLI	PUBLIC
1095	150	CAMPANIA	150418	PRESIDIO OSPEDALIERO NAPOLI EST	VIA F.M. BRIGANTI,255	80144	NAPOLI	PUBLIC
1096	150	CAMPANIA	150418	PRESIDIO OSPEDALIERO NAPOLI EST	VIA F.M. BRIGANTI,255	80144	NAPOLI	PUBLIC
1097	150	CAMPANIA	150418	PRESIDIO OSPEDALIERO NAPOLI EST	VIA F.M. BRIGANTI,255	80144	NAPOLI	PUBLIC
1098	150	CAMPANIA	150418	PRESIDIO OSPEDALIERO NAPOLI EST	VIA F.M. BRIGANTI,255	80144	NAPOLI	PUBLIC
1099	150	CAMPANIA	150418	PRESIDIO OSPEDALIERO NAPOLI EST	VIA F.M. BRIGANTI,255	80144	NAPOLI	PUBLIC
1100	150	CAMPANIA	150418	PRESIDIO OSPEDALIERO NAPOLI EST	VIA F.M. BRIGANTI,255	80144	NAPOLI	PUBLIC
1101	150	CAMPANIA	150418	PRESIDIO OSPEDALIERO NAPOLI EST	VIA F.M. BRIGANTI,255	80144	NAPOLI	PUBLIC
1102	150	CAMPANIA	150419	PRESIDIO OSPEDALIERO NAPOLI OVEST	VIA TERRACINA 219	80125	NAPOLI	PUBLIC
1103	150	CAMPANIA	150419	PRESIDIO OSPEDALIERO NAPOLI OVEST	VIA TERRACINA 219	80125	NAPOLI	PUBLIC
1104	150	CAMPANIA	150419	PRESIDIO OSPEDALIERO NAPOLI OVEST	VIA TERRACINA 219	80125	NAPOLI	PUBLIC
1105	150	CAMPANIA	150419	PRESIDIO OSPEDALIERO NAPOLI OVEST	VIA TERRACINA 219	80125	NAPOLI	PUBLIC
1106	150	CAMPANIA	150419	PRESIDIO OSPEDALIERO NAPOLI OVEST	VIA TERRACINA 219	80125	NAPOLI	PUBLIC
1107	150	CAMPANIA	150419	PRESIDIO OSPEDALIERO NAPOLI OVEST	VIA TERRACINA 219	80125	NAPOLI	PUBLIC
1108	150	CAMPANIA	150058	OSPEDALE SAN GIULIANO	VIA G.B. BASILE	80014	GIUGLIANO IN CAMPANIA	PUBLIC
1109	150	CAMPANIA	150070	OSPEDALE RIZZOLI	VIA FUNDERA LACCO AMENO	80076	LACCO AMENO	PUBLIC
1110	150	CAMPANIA	150097	CASA DI CURA VILLA MAIONE	VIA E.FERMI 59	80010	VILLARICCA	PRIVATE
1111	150	CAMPANIA	150116	CASA DI CURA VILLA DEI FIORI	CORSO ITALIA 110	80018	MUGNANO DI NAPOLI	PRIVATE
1112	150	CAMPANIA	150189	OSPEDALE CIVILE ALBANO FRANCESCANO	CORSO VITT.EMANUELE	80074	PROCIDA	PUBLIC
1113	150	CAMPANIA	150191	OSPEDALE S.M.DELLE GRAZIE	VIA DOMITIANA LOCALITA` LA SCHIANA	80078	POZZUOLI	PUBLIC

1114	150	CAMPANIA	150057	OSPEDALE CIVILE S. GIOVANNI DI DIO	VIA PIROZZI, 66	80027	FRATTAMAGGIORE	PUBLIC
1115	150	CAMPANIA	150101	CASA DI CURA 'CLINICA S.ANTIMO'	VIA GIOVANNI FALCONE N.121(EX VIA ROM	80025	CASANDRINO	PRIVATE
1116	150	CAMPANIA	150118	ISTITUTO DI CURA MARIA SS.DELLA PIETA`	VIA S. ROCCO N.9	80026	CASORIA	PUBLIC
1117	150	CAMPANIA	150064	PRES. OSPEDALIERO S. MARIA DELLA PIETA`	VIA SEMINARIO	80035	NOLA	PUBLIC
1118	150	CAMPANIA	150066	OSPEDALE CAV RAFFAELE APICELLA	VIA MASSA N. 1	80040	POLLENA TROCCHIA	PUBLIC
1119	150	CAMPANIA	150082	CASA DI CURA ' LA MADONNINA ' SRL	VIA ROMA N.29	80040	SAN GENNARO VESUVIANO	PRIVATE
1120	150	CAMPANIA	150084	CASA DI CURA VILLA DEI FIORI SRL	CORSO ITALIA N.1	80011	ACERRA	PRIVATE
1121	150	CAMPANIA	150100	CASA DI CURA ' N.S. DI LOURDES ' SPA	VIA TULLIO BOCCARUSSO N.1	80040	MASSA DI SOMMA	PRIVATE
1122	150	CAMPANIA	150107	CASA DI CURA ' VILLA ELISA '	VIA NAZ.LE DELLE PUGLIE KM.54,700	80032	CASAMARCIANO	PRIVATE
1123	150	CAMPANIA	150108	CASA DI CURA TRUSSO S.R.L.	VIA SAN GIOVANNI BOSCO N.3	80044	OTTAVIANO	PRIVATE
1124	150	CAMPANIA	150113	CASA DI CURA S.LUCIA SRL	VIA AIELLI	80047	SAN GIUSEPPE VESUVIANO	PRIVATE
1125	150	CAMPANIA	150117	CASA DI CURA ' MELUCCIO ' S.R.L.	VIA F. PIROZZI N.20	80038	POMIGLIANO D`ARCO	PRIVATE
1126	150	CAMPANIA	150121	CLINICA S. FELICE S.R.L.	VIA MAURO LEONE N.106	80038	POMIGLIANO D`ARCO	PRIVATE
1127	150	CAMPANIA	150192	CASA DI CURA S.MARIA DEL POZZO	VIA POMIGLIANO N.40	80049	SOMMA VESUVIANA	PRIVATE
1128	150	CAMPANIA	150087	CASA DI CURA S.MICHELE	VIA C. AMALFI13	80063	PIANO DI SORRENTO	PRIVATE
1129	150	CAMPANIA	150102	CASA DI CURA S. MARIA LA BRUNA SRL	VIA NAZIONALE 627	80059	TORRE DEL GRECO	PRIVATE
1130	150	CAMPANIA	150105	CASA DI CURA VILLA STABIA	VIALE EUROPA 77	80053	CASTELLAMMARE DI STABIA	PRIVATE
1131	150	CAMPANIA	150110	CASA DI CURA MARIA ROSARIA SPA	VIA COLLE S. BARTOLOMEO 50	80045	POMPEI	PRIVATE
1132	150	CAMPANIA	150111	STAZIONE CLIMATICA BIANCHI	VIA LIBERTA`,342	80055	PORTICI	PRIVATE
1133	150	CAMPANIA	150114	CASA DI CURA A.GRIMALDI	VIA MARCONI 10	80046	SAN GIORGIO A CREMANO	PRIVATE
1134	150	CAMPANIA	150115	CASA DI CURA VILLA DELLE MARGHERITE SNC	VIA SCAPPI 18	80059	TORRE DEL GRECO	PRIVATE
1135	150	CAMPANIA	150377	PARK HOSPITAL SERVICE SRL	VIA PLINIO IL VECCHIO 40	80046	SAN SEBASTIANO AL VESUVIO	PRIVATE
1136	150	CAMPANIA	150415	OSPEDALI RIUNITI GOLFO VESUVIANO	Via Montedoro	80059	TORRE DEL GRECO	PUBLIC
1137	150	CAMPANIA	150415	OSPEDALI RIUNITI GOLFO VESUVIANO	Via Montedoro	80059	TORRE DEL GRECO	PUBLIC
1138	150	CAMPANIA	150416	OSPEDALI RIUNITI AREA STABIESE	VIALE EUROPA	80053	CASTELLAMMARE DI STABIA	PUBLIC
1139	150	CAMPANIA	150416	OSPEDALI RIUNITI AREA STABIESE	VIALE EUROPA	80053	CASTELLAMMARE DI STABIA	PUBLIC
1140	150	CAMPANIA	150417	OSPEDALI RIUNITI PENISOLA SORRENTINA	CORSO ITALIA 1	80067	SORRENTO	PUBLIC
1141	150	CAMPANIA	150417	OSPEDALI RIUNITI PENISOLA SORRENTINA	CORSO ITALIA 1	80067	SORRENTO	PUBLIC
1142	150	CAMPANIA	150153	P.O. S. MARIA DELL`OLMO CAVA	VIA DE MARINIS	84013	CAVA DE` TIRRENI	PUBLIC
1143	150	CAMPANIA	150153	P.O. S. MARIA DELL`OLMO CAVA	VIA DE MARINIS	84013	CAVA DE` TIRRENI	PUBLIC
1144	150	CAMPANIA	150154	P.O. UMBERTO I	VIA IODICE	84014	NOCERA INFERIORE	PUBLIC
1145	150	CAMPANIA	150159	P.O. ANDREA TORTORA	VIA DE GASPERI 1	84015	PAGANI	PUBLIC
1146	150	CAMPANIA	150163	P.O. VILLA MALTA	P.ZA GARIBALDI	84087	SARNO	PUBLIC
1147	150	CAMPANIA	150164	P.O. MAURO SCARLATO SCAFATI	VIA PASSANTI 2	84018	SCAFATI	PUBLIC
1148	150	CAMPANIA	150178	CASA DI CURA VILLA CHIARUGI	VIA GIUSEPPE ATZORI,283	84014	NOCERA INFERIORE	PRIVATE
1149	150	CAMPANIA	150158	P.O. SAN FRANCESCO D`ASSISI	VIA M .CLEMENTE	84020	OLIVETO CITRA	PUBLIC
1150	150	CAMPANIA	150162	PRESIDIO OSPEDALIERO MARIA SS. ADDOLORA	PZA SCUOLA MEDICA SALERNITANA	84025	EBOLI	PUBLIC
1151	150	CAMPANIA	150166	PRESIDIO OSPEDALIERO S.MARIA SPERANZA	VIA FIORIGNANO	84091	BATTIPAGLIA	PUBLIC
1152	150	CAMPANIA	150167	HIPPOCRATICA SPA VILLA DEL SOLE	VIA BELVEDERE 31	84135	SALERNO	PRIVATE
1153	150	CAMPANIA	150171	CASA DI CURA LA QUIETE SRL	VIA G. AMENDOLA 1-CAPEZZANO	84080	PELLEZZANO	PRIVATE
1154	150	CAMPANIA	150172	CASA DI CURA VENOSA SRL	VIA MAZZINI 34/36	84091	BATTIPAGLIA	PRIVATE
1155	150	CAMPANIA	150173	CASA DI CURA PRIVATA SALUS	VIA F. CONFALONIERI 4	84091	BATTIPAGLIA	PRIVATE
1156	150	CAMPANIA	150175	CAMPOLONGO HOSPITAL SPA C.E.M.F.R. EBOLI	VIA MARINA8712 LOC CAMPOLONGO	84025	EBOLI	PRIVATE
1157	150	CAMPANIA	150177	CASA DI CURA TORTORELLA SPA	VIA N. AVERSANO 1	84127	SALERNO	PRIVATE
1158	150	CAMPANIA	150183	OSPEDALE AMICO G.FUCITO_M.S.SEVERINO	C.SO UMBERTO I	84085	MERCATO SAN SEVERINO	PUBLIC
1159	150	CAMPANIA	150187	P.O.G.DA PROCIDA	VIA SALVATORE CALENDA 162	84126	SALERNO	PUBLIC
1160	150	CAMPANIA	150160	PRESIDIO OSPEDALIERO DI POLLA-S. ARSENIO	VIA L.CURTO	84035	POLLA	PUBLIC
1161	150	CAMPANIA	150161	PRESIDIO OSPEDALIERO DELL`IMMACOLATA	VIA VERDI	84073	SAPRI	PUBLIC
1162	150	CAMPANIA	150165	OSPEDALE S.LUCA	VIA F.CAMMAROTA	84078	VALLO DELLA LUCANIA	PUBLIC
1163	150	CAMPANIA	150170	CASA DI CURA MALZONI	VIA GIANBATTISTA VICO	84043	AGROPOLI	PRIVATE
1164	150	CAMPANIA	150176	CASA DI CURA COBELLIS	C/DA BADIA	84078	VALLO DELLA LUCANIA	PRIVATE
1165	150	CAMPANIA	150387	OSPEDALE DI ROCCADASPIDE	VIA SANTA PALOMBA	84069	ROCCADASPIDE	PUBLIC
1166	150	CAMPANIA	150404	OSPEDALE CIVILE DI AGROPOLI	CONTRAD MARROTA	84043	AGROPOLI	PUBLIC
1167	150	CAMPANIA	150901	AZIENDA OSPEDALIERA 'A. CARDARELLI'	VIA A.CARDARELLI 9	80131	NAPOLI	PUBLIC
1168	150	CAMPANIA	150902	A.O.SANTOBONO-PAUSILIPON	VIA CROCE ROSSA 8	80122	NAPOLI	PUBLIC
1169	150	CAMPANIA	150902	A.O.SANTOBONO-PAUSILIPON	VIA CROCE ROSSA 8	80122	NAPOLI	PUBLIC
1170	150	CAMPANIA	150903	AZIENDA OSPEDALIERA VINCENZO MONALDI	VIA LEONARDO BIANCHI 29	80131	NAPOLI	PUBLIC
1171	150	CAMPANIA	150904	A.O.OO.RR.S.GIOVANNI DI DIO E RUGGI D`AR	S.LEONARDO	84100	SALERNO	PUBLIC
1172	150	CAMPANIA	150905	AZIENDA OSPEDALIERA S.G. MOSCATI	VIA OTRANTO	83100	AVELLINO	PUBLIC
1173	150	CAMPANIA	150906	AZIENDA OSPEDALE `G.RUMMO`	VIA DELL`ANGELO,1	82100	BENEVENTO	PUBLIC
1174	150	CAMPANIA	150907	A.O. SANT'ANNA E SAN SEBASTIANO CASERTA	VIA PALASCIANO	81100	CASERTA	PUBLIC
1175	150	CAMPANIA	150908	AZIENDA UNIVERSITARIA POLICLINICO	VIA COSTANTINOPOLI, 104	80138	NAPOLI	PUBLIC
1176	150	CAMPANIA	150909	UNIV.STUDI NAPOLI-FEDERICO II-FAC.MEDIC.	VIA SERGIO PANSINI 5	80100	NAPOLI	PUBLIC

1177	150	CAMPANIA	150910	ISTITUTO NAZIONALE TUMORI DI NAPOLI	VIA MARIANO SEMMOLA	80131	NAPOLI	PUBLIC
1178	150	CAMPANIA	150912	AZIENDA OSPEDALIERA COTUGNO	VIA G. QUAGLIARIELLO, 54	80131	NAPOLI	PUBLIC
1179	160	PUGLIA	160101	CASA DI CURA 'SALUS' - BRINDISI	VIA APPIA, 366	72100	BRINDISI	PRIVATE
1180	160	PUGLIA	160151	ASS.LA NOSTRA FAMIGLIA IRCCS 'E.MEDEA'	VIA DEI COLLI, 5/7	72017	OSTUNI	PUBLIC
1181	160	PUGLIA	160161	P.O. OSTUNI-FASANO-CISTERNINO	VIA VILLAFRANCA	72017	OSTUNI	PUBLIC
1182	160	PUGLIA	160161	P.O. OSTUNI-FASANO-CISTERNINO	VIA VILLAFRANCA	72017	OSTUNI	PUBLIC
1183	160	PUGLIA	160161	P.O. OSTUNI-FASANO-CISTERNINO	VIA VILLAFRANCA	72017	OSTUNI	PUBLIC
1184	160	PUGLIA	160162	P.O. FRANCAVILLA FONT.- CEGLIE MESSAPICA	VIA PROVINCIALE PER CEGLIE MESSAPICA	72021	FRANCAVILLA FONTANA	PUBLIC
1185	160	PUGLIA	160162	P.O. FRANCAVILLA FONT.- CEGLIE MESSAPICA	VIA PROVINCIALE PER CEGLIE MESSAPICA	72021	FRANCAVILLA FONTANA	PUBLIC
1186	160	PUGLIA	160170	P.O.BRINDISI-MESAGNE-S.PIETRO V.-CEGLIE	S.S.7 PER MESAGNE	72100	BRINDISI	PUBLIC
1187	160	PUGLIA	160170	P.O.BRINDISI-MESAGNE-S.PIETRO V.-CEGLIE	S.S.7 PER MESAGNE	72100	BRINDISI	PUBLIC
1188	160	PUGLIA	160170	P.O.BRINDISI-MESAGNE-S.PIETRO V.-CEGLIE	S.S.7 PER MESAGNE	72100	BRINDISI	PUBLIC
1189	160	PUGLIA	160170	P.O.BRINDISI-MESAGNE-S.PIETRO V.-CEGLIE	S.S.7 PER MESAGNE	72100	BRINDISI	PUBLIC
1190	160	PUGLIA	160074	PRESIDIO OSPEDALIERO ORIENTALE	VIA MANDONION	74024	MANDURIA	PUBLIC
1191	160	PUGLIA	160075	PRESIDIO VALLE D`ITRIA.	P.ZZA SAN FRANCESCO DA PAOLA	74015	MARTINA FRANCA	PUBLIC
1192	160	PUGLIA	160111	CASA DI CURA BERNARDINI	Via Scoglio del Tonno, n. 64	74100	TARANTO	PRIVATE
1193	160	PUGLIA	160112	CASA DI CURA D`AMORE SRL	VIALE MAGNA GRECIA 62	74100	TARANTO	PRIVATE
1194	160	PUGLIA	160113	CASA DI CURA MATER DEI	VIA SOLITO N. 14	74100	TARANTO	PRIVATE
1195	160	PUGLIA	160114	CASA DI CURA SAN CAMILLO	VIA MASACCIO N. 12	74100	TARANTO	PRIVATE
1196	160	PUGLIA	160115	CASA DI CURA SANTA RITA S.R.L.	VIALE MAGNA GRECIA N. 191	74100	TARANTO	PRIVATE
1197	160	PUGLIA	160116	CASA DI CURA VILLA VERDE S.N.C.	VIA GOLFO DI TARANTO N. 22	74100	TARANTO	PRIVATE
1198	160	PUGLIA	160141	CASA DI CURA VILLA BIANCA S.R.L.	C/DA TAGLIENTE - SAN PAOLO	74015	MARTINA FRANCA	PRIVATE
1199	160	PUGLIA	160146	CENTRO MEDICO DI RIABILITAZIONE (C.M.R.)	VIA DELLA CHIESA N. 4	74025	GINOSA	PRIVATE
1200	160	PUGLIA	160149	FONDAZIONE CITTADELLA DELLA CARITA`	PIAZZALE CITTADELLA DELLA CARITA` N. 1	74100	TARANTO	PRIVATE
1201	160	PUGLIA	160168	PRESIDIO OSPEDALIERO OCCIDENTALE	VIA DEL MERCATO 60	74011	CASTELLANETA	PUBLIC
1202	160	PUGLIA	160168	PRESIDIO OSPEDALIERO OCCIDENTALE	VIA DEL MERCATO 60	74011	CASTELLANETA	PUBLIC
1203	160	PUGLIA	160168	PRESIDIO OSPEDALIERO OCCIDENTALE	VIA DEL MERCATO 60	74011	CASTELLANETA	PUBLIC
1204	160	PUGLIA	160172	PRESIDIO OSPEDALIERO CENTRALE	VIA BRUNO	74100	TARANTO	PUBLIC
1205	160	PUGLIA	160172	PRESIDIO OSPEDALIERO CENTRALE	VIA BRUNO	74100	TARANTO	PUBLIC
1206	160	PUGLIA	160174	OSPEDALE 'L. BONOMO' - ANDRIA	VIALE ISTRIA 1	70031	ANDRIA	PUBLIC
1207	160	PUGLIA	160175	OSP. CANOSA-MINERVINO-SPINAZZOLA	VIA G. BOVIO, 81	70053	CANOSA DI PUGLIA	PUBLIC
1208	160	PUGLIA	160175	OSP. CANOSA-MINERVINO-SPINAZZOLA	VIA G. BOVIO, 81	70053	CANOSA DI PUGLIA	PUBLIC
1209	160	PUGLIA	160175	OSP. CANOSA-MINERVINO-SPINAZZOLA	VIA G. BOVIO, 81	70053	CANOSA DI PUGLIA	PUBLIC
1210	160	PUGLIA	160177	OSP. "MONS. R. DIMICCOLI" - BARLETTA	VIALE IPPOCRATE 15	70051	BARLETTA	PUBLIC
1211	160	PUGLIA	160178	OSP. BISCEGLIE - TRANI	VIA G. BOVIO 279/A	70052	BISCEGLIE	PUBLIC
1212	160	PUGLIA	160178	OSP. BISCEGLIE - TRANI	VIA G. BOVIO 279/A	70052	BISCEGLIE	PUBLIC
1213	160	PUGLIA	160180	CASA DIVINA PROVVIDENZA - BISCEGLIE	VIA G. BOVIO 76/A	70052	BISCEGLIE	PRIVATE
1214	160	PUGLIA	160078	ENTE ECCL.OSP.GEN.REG.'MIULLI'	S.P. ACQUAVIVA SANTERAMO, 127	70021	ACQUAVIVA DELLE FONTI	PUBLIC
1215	160	PUGLIA	160086	CASA DI CURA LA MADONNINA	V.LE PASTEUR 18	70124	BARI	PRIVATE
1216	160	PUGLIA	160087	SANTA MARIA	VIA A. DE FERRARIIS 18/D	70100	BARI	PRIVATE
1217	160	PUGLIA	160091	C.B.H. PRESIDIO S. RITA	VIA G. PETRONI 132/G	70100	BARI	PRIVATE
1218	160	PUGLIA	160092	CASA DI CURA TORREBELLA	VIA GENERALE BELLOMO,87	70124	BARI	PRIVATE
1219	160	PUGLIA	160093	C.B.H. PRESIDIO VILLA DEL SOLE	C.SO A. DE GASPERI 142	70100	BARI	PRIVATE
1220	160	PUGLIA	160094	C.B.H. PRESIDIO VILLA LUCE	VIA NAPOLI 8	70100	BARI	PRIVATE
1221	160	PUGLIA	160095	VILLA SERENA	STRADELLA CARDUCCI 8/A	70125	BARI	PRIVATE
1222	160	PUGLIA	160098	CASA DI CURA 'VILLA LUCIA'	VIA LACALANDRA	70014	CONVERSANO	PRIVATE
1223	160	PUGLIA	160099	CASA CURA VILLA GIUSTINA	VIA VOLPICELLA 12/A	70056	MOLFETTA	PRIVATE
1224	160	PUGLIA	160100	CASA DI CURA ' MONTE IMPERATORE'	STRADA PROV. NOCI - CASTELLANETA KM.	70015	NOCI	PRIVATE
1225	160	PUGLIA	160138	C.B.H. PRESIDIO VILLA BIANCA	VIA SCIPIONE L`AFRICANO 191	70100	BARI	PRIVATE
1226	160	PUGLIA	160140	CASA DI CURA ANTHEA	VIA C. ROSALBA 35/37	70124	BARI	PRIVATE
1227	160	PUGLIA	160145	CASA BIANCA	VIA VITTORIO EMANUELE II, 2	70020	CASSANO DELLE MURGE	PRIVATE
1228	160	PUGLIA	160147	C.B.H. PRESIDIO MATER DEI	VIA AMENDOLA 209	70100	BARI	PRIVATE
1229	160	PUGLIA	160156	PO MOLFETTA	VIA TERLIZZI	70056	MOLFETTA	PUBLIC
1230	160	PUGLIA	160157	PRESIDIO OSPEDALE UNICO AUSL BA/3	VIALE REGINA MARGHERITA	70022	ALTAMURA	PUBLIC
1231	160	PUGLIA	160157	PRESIDIO OSPEDALE UNICO AUSL BA/3	VIALE REGINA MARGHERITA	70022	ALTAMURA	PUBLIC
1232	160	PUGLIA	160157	PRESIDIO OSPEDALE UNICO AUSL BA/3	VIALE REGINA MARGHERITA	70022	ALTAMURA	PUBLIC
1233	160	PUGLIA	160158	P.O. S. PAOLO	VIA CAPOSCARDICCHIO S.N.	70123	BARI	PUBLIC
1234	160	PUGLIA	160159	P. O. MONOPOLI - CONVERSANO	LARGO SIMONE VENEZIANI N[21	70043	MONOPOLI	PUBLIC
1235	160	PUGLIA	160159	P. O. MONOPOLI - CONVERSANO	LARGO SIMONE VENEZIANI N[21	70043	MONOPOLI	PUBLIC
1236	160	PUGLIA	160160	P.O. PUTIGNANO-NOCI-GIOIA DEL COLLE	VIA CAPPUCCINI	70017	PUTIGNANO	PUBLIC
1237	160	PUGLIA	160160	P.O. PUTIGNANO-NOCI-GIOIA DEL COLLE	VIA CAPPUCCINI	70017	PUTIGNANO	PUBLIC
1238	160	PUGLIA	160160	P.O. PUTIGNANO-NOCI-GIOIA DEL COLLE	VIA CAPPUCCINI	70017	PUTIGNANO	PUBLIC
1239	160	PUGLIA	160169	P.O. BARI SUD	VIA OSPEDALE DI VENERE	70012	BARI	PUBLIC

1240	160	PUGLIA	160169	P.O. BARI SUD	VIA OSPEDALE DI VENERE	70012	BARI	PUBLIC
1241	160	PUGLIA	160169	P.O. BARI SUD	VIA OSPEDALE DI VENERE	70012	BARI	PUBLIC
1242	160	PUGLIA	160176	PO CORATO-RUVO	VIA RUVO, 108	70033	CORATO	PUBLIC
1243	160	PUGLIA	160176	PO CORATO-RUVO	VIA RUVO, 108	70033	CORATO	PUBLIC
1244	160	PUGLIA	160179	PO TERLIZZI-BITONTO	VIA PASQUALE FIORE, 135	70038	TERLIZZI	PUBLIC
1245	160	PUGLIA	160179	PO TERLIZZI-BITONTO	VIA PASQUALE FIORE, 135	70038	TERLIZZI	PUBLIC
1246	160	PUGLIA	160047	OSPEDALE 'G.TATARELLA'	VIA TRINITAPOLI	71042	CERIGNOLA	PUBLIC
1247	160	PUGLIA	160048	OSPEDALE 'FRANCESCO LASTARIA'	VIA F. LASTARIA	71036	LUCERA	PUBLIC
1248	160	PUGLIA	160102	CASA DI CURA PROF. BRODETTI	VIA VITTIMI CIVILI 112	71100	FOGGIA	PRIVATE
1249	160	PUGLIA	160105	CASA DI CURA LEONARDO DE LUCA	VIA POGGIO DELLA BRECCIOSA	71034	CASTELNUOVO DELLA DAUNIA	PRIVATE
1250	160	PUGLIA	160106	CASA DI CURA 'S.MICHELE' MANFREDONIA	VIA COZZOLETE N. 29	71043	MANFREDONIA	PRIVATE
1251	160	PUGLIA	160124	CENTRO RIABIL. S. MARIA	VIA LUCERA 110	71100	FOGGIA	PRIVATE
1252	160	PUGLIA	160163	PRESIDIO OSPEDALIERO TERESA MASSELLI	VIA TERESA MASSELLI	71016	SAN SEVERO	PUBLIC
1253	160	PUGLIA	160163	PRESIDIO OSPEDALIERO TERESA MASSELLI	VIA TERESA MASSELLI	71016	SAN SEVERO	PUBLIC
1254	160	PUGLIA	160163	PRESIDIO OSPEDALIERO TERESA MASSELLI	VIA TERESA MASSELLI	71016	SAN SEVERO	PUBLIC
1255	160	PUGLIA	160164	OSPEDALE MANFREDONIA-MONTE S.ANGELO	VIA ISONZO	71043	MANFREDONIA	PUBLIC
1256	160	PUGLIA	160164	OSPEDALE MANFREDONIA-MONTE S.ANGELO	VIA ISONZO	71043	MANFREDONIA	PUBLIC
1257	160	PUGLIA	160181	CASE CURA RIUNITE VILLA SERENA-S. FRANCE	VIALE EUROPA, 12	71100	FOGGIA	PRIVATE
1258	160	PUGLIA	160062	P.O. S.CATERINA NOVELLA - GALATINA	VIA ROMA	73013	GALATINA	PUBLIC
1259	160	PUGLIA	160063	PRESIDIO OSP. SACRO CUORE DI GESU`	STRADA PROV.LE ALEZIO-GALLIPOLI	73014	GALLIPOLI	PUBLIC
1260	160	PUGLIA	160080	OSPEDALE GENERALE PROV.CARD. G. PANICO	VIA SAN PIO X 4	73039	TRICASE	PUBLIC
1261	160	PUGLIA	160107	CASA DI CURA PETRUCCIANI	VIA ALDO MORO snc	73100	LECCE	PRIVATE
1262	160	PUGLIA	160108	CASA DI CURA VILLA BIANCA	VIA LEUCA 133	73100	LECCE	PRIVATE
1263	160	PUGLIA	160109	CASA DI CURA VILLA VERDE	VIA MONTERONI 222	73100	LECCE	PRIVATE
1264	160	PUGLIA	160110	CASA DI CURA SAN FRANCESCO	P/ZZA F.CESARI 4	73013	GALATINA	PRIVATE
1265	160	PUGLIA	160150	CASA DI CURA CITTA DI LECCE	VIA PROVINCIALE PER ARNESANO	73100	LECCE	PRIVATE
1266	160	PUGLIA	160152	CASA DI CURA RIABILITATIVA EUROITALIA	VIA PROV. LE PER COLLEPASSO	73042	CASARANO	PRIVATE
1267	160	PUGLIA	160165	COPERTINO-NARDO	VIA CARMIANO	73043	COPERTINO	PUBLIC
1268	160	PUGLIA	160165	COPERTINO-NARDO	VIA CARMIANO	73043	COPERTINO	PUBLIC
1269	160	PUGLIA	160166	P.O.DI SCORRANO-MAGLIE-POGGIARDO	VIA DELLI PONTI	73020	SCORRANO	PUBLIC
1270	160	PUGLIA	160166	P.O.DI SCORRANO-MAGLIE-POGGIARDO	VIA DELLI PONTI	73020	SCORRANO	PUBLIC
1271	160	PUGLIA	160166	P.O.DI SCORRANO-MAGLIE-POGGIARDO	VIA DELLI PONTI	73020	SCORRANO	PUBLIC
1272	160	PUGLIA	160167	P.O. DI CASARANO-GAGLIANO	VIALE FRANCESCO FERRARI	73042	CASARANO	PUBLIC
1273	160	PUGLIA	160167	P.O. DI CASARANO-GAGLIANO	VIALE FRANCESCO FERRARI	73042	CASARANO	PUBLIC
1274	160	PUGLIA	160171	P.O. N. 1 V. FAZZI - S.CESARIO - CAMPI	VIA F. MURATORE	73100	LECCE	PUBLIC
1275	160	PUGLIA	160171	P.O. N. 1 V. FAZZI - S.CESARIO - CAMPI	VIA F. MURATORE	73100	LECCE	PUBLIC
1276	160	PUGLIA	160171	P.O. N. 1 V. FAZZI - S.CESARIO - CAMPI	VIA F. MURATORE	73100	LECCE	PUBLIC
1277	160	PUGLIA	160901	ISTITUTO TUMORI GIOVANNI PAOLO II	VIA S. F. HAHNEMANN	70126	BARI	PUBLIC
1278	160	PUGLIA	160902	IRCCS "SAVERIO DE BELLIS"	Via F. VALENTE, 4	70013	CASTELLANA GROTTE	PUBLIC
1279	160	PUGLIA	160905	OSPEDALE CASA SOLLIEVO DELLA SOFFERENZA	VIALE CAPPUCCINI	71013	SAN GIOVANNI ROTONDO	PUBLIC
1280	160	PUGLIA	160906	FONDAZIONE SALVATORE MAUGERI	VIA PER MERCADANTE KM 2	70020	CASSANO DELLE MURGE	PUBLIC
1281	160	PUGLIA	160907	AO Univ Consorziale Policlinico di Bari	P.zza G. Cesare 11	70124	BARI	PUBLIC
1282	160	PUGLIA	160907	AO Univ Consorziale Policlinico di Bari	P.zza G. Cesare 11	70124	BARI	PUBLIC
1283	160	PUGLIA	160910	Azienda Ospedaliera"Ospedali Riuniti"	Viale Luigi Pinto	71100	FOGGIA	PUBLIC
1284	170	BASILICATA	170024	PRESIDIO OSPEDALIERO UNIFICATO	VIA FOGGIA	85025	MELFI	PUBLIC
1285	170	BASILICATA	170024	PRESIDIO OSPEDALIERO UNIFICATO	VIA FOGGIA	85025	MELFI	PUBLIC
1286	170	BASILICATA	170008	OSPEDALE CIVILE VILLA D`AGRI	VIA PROVINCIALE -MARSICOVETERE	85050	MARSICOVETERE	PUBLIC
1287	170	BASILICATA	170009	CASA DI CURA LUCCIONI-POTENZA	VIALE MAZZINI, 52	85100	POTENZA	PRIVATE
1288	170	BASILICATA	170032	Fondazione Don Carlo Gnocchi - Onlus	C.da Gala	85011	ACERENZA	PUBLIC
1289	170	BASILICATA	170006	PRESIDIO OSPEDALIERO DI CHIAROMONTE	VIA SANTA LUCIA	85032	CHIAROMONTE	PUBLIC
1290	170	BASILICATA	170020	OSPEDALI UNIFICATI DEL LAGONEGRESE	VIALE COLOMBO	85042	LAGONEGRO	PUBLIC
1291	170	BASILICATA	170020	OSPEDALI UNIFICATI DEL LAGONEGRESE	VIALE COLOMBO	85042	LAGONEGRO	PUBLIC
1292	170	BASILICATA	170020	OSPEDALI UNIFICATI DEL LAGONEGRESE	VIALE COLOMBO	85042	LAGONEGRO	PUBLIC
1293	170	BASILICATA	170011	PRESIDIO OSPEDALIERO - MATERA	C.DA CATTEDRA AMBULANTE	75100	MATERA	PUBLIC
1294	170	BASILICATA	170012	PRESIDIO OSPEDALIERO - TRICARICO	V.LE REGINA MARGHERITA,134	75019	TRICARICO	PUBLIC
1295	170	BASILICATA	170025	PRESIDIO OSPEDALIERO POLICORO	VIALE SALERNO	75025	POLICORO	PUBLIC
1296	170	BASILICATA	170025	PRESIDIO OSPEDALIERO POLICORO	VIALE SALERNO	75025	POLICORO	PUBLIC
1297	170	BASILICATA	170025	PRESIDIO OSPEDALIERO POLICORO	VIALE SALERNO	75025	POLICORO	PUBLIC
1298	170	BASILICATA	170901	AZIENDA OSPEDALIERA OSPEDALE S.CARLO	C/DA MACCHIA ROMANA POTENZA	85100	POTENZA	PUBLIC
1299	170	BASILICATA	170901	AZIENDA OSPEDALIERA OSPEDALE S.CARLO	C/DA MACCHIA ROMANA POTENZA	85100	POTENZA	PUBLIC
1300	170	BASILICATA	170902	OSPEDALE ONCOLOGICO REGIONALE	STRADA PROVINCIALE N.8 DEL VULTURE	85028	RIONERO IN VULTURE	PUBLIC
1301	180	CALABRIA	180059	OSPEDALE CIVILE LOCRI	C/DA VERGA	89044	LOCRI	PUBLIC
1302	180	CALABRIA	180069	OSPEDALE CIVILE SIDERNO	VIALE EUROPA	89048	SIDERNO	PUBLIC

1303	180	CALABRIA	180070	OSPEDALE CIVILE GERACE	C/O PRES. OSPED. DI LOCRI C.DA VERGA	89044	LOCRI	PUBLIC
1304	180	CALABRIA	180003	STABILIMENTO OSPEDALIERO CORIGLIANO	VIALE RIMEMBRANZE	87064	CORIGLIANO CALABRO	PUBLIC
1305	180	CALABRIA	180004	STABILIMENTO OSPEDALIERO ROSSANO	VIA IPPOCRATE	87068	ROSSANO	PUBLIC
1306	180	CALABRIA	180005	PRESIDIO OSPEDALIERO DI CETRARO	LOCALIA` TESTA CETRARO	87022	CETRARO	PUBLIC
1307	180	CALABRIA	180006	OSPEDALE CIVILE FERRARI - CASTROVILLARI	VIALE DEL LAVORO	87012	CASTROVILLARI	PUBLIC
1308	180	CALABRIA	180007	PRESIDIO OSPEDALIERO SAN FRANCESCO PAOLA	VIA PROMINTESTA PAOLA	87027	PAOLA	PUBLIC
1309	180	CALABRIA	180008	OSPEDALE CIVILE PRAIA A MARE	C.DA S.STEFANO	87028	PRAIA A MARE	PUBLIC
1310	180	CALABRIA	180009	OSPEDALE GENERALE DI ZONA-LUNGRO	CONTRADA SAN LEONARDO	87010	LUNGRO	PUBLIC
1311	180	CALABRIA	180011	P.O.BEATO ANGELICO	VIA IPPOCRATE	87041	ACRI	PUBLIC
1312	180	CALABRIA	180013	OSPEDALE CIVILE MINERVINI	VIA STAZIONE	87026	MORMANNO	PUBLIC
1313	180	CALABRIA	180014	STABILIMENTO OSPEDALIERO CARIATI	S.S. 106	87063	CARIATI	PUBLIC
1314	180	CALABRIA	180015	OSPEDALE CIVILE	VIA GRAMSCI	87055	SAN GIOVANNI IN FIORE	PUBLIC
1315	180	CALABRIA	180016	STABILIMENTO OSPEDALIERO TREBISACCE	VIA VICCINELLI	87075	TREBISACCE	PUBLIC
1316	180	CALABRIA	180017	SANTA LUCIA	VIALE TRIESTE	87100	COSENZA	PRIVATE
1317	180	CALABRIA	180018	CASA DI CURA VILLA VERDE	C.DA FIEGO DONNICI INFERIORE	87030	COSENZA	PRIVATE
1318	180	CALABRIA	180019	CASA DI CURA VILLA DEL SOLE	RIONE S. VITO	87100	COSENZA	PRIVATE
1319	180	CALABRIA	180021	LA MADONNINA S.R.L.	VIA PAOLO ROSSI 109	87100	COSENZA	PRIVATE
1320	180	CALABRIA	180022	G. SANTORO	VIA ISONZO 38	87100	COSENZA	PRIVATE
1321	180	CALABRIA	180023	CLINICA MADONNA DELLE GRAZIE	P.LE F.SCO TOSCANO 1 - SIBARI	87011	CASSANO ALLO IONIO	PRIVATE
1322	180	CALABRIA	180024	VILLA ORTENSIA	VIA ARIOSTO 10	87100	COSENZA	PRIVATE
1323	180	CALABRIA	180025	ISTITUTO 'NINETTA ROSANO'	VIA CAPOTIRONE N 12/20 BELVEDERE M.MO	87021	BELVEDERE MARITTIMO	PRIVATE
1324	180	CALABRIA	180026	CASA DI CURA SPINELLI S.R.L.	VIA CASTEL RUGIERO,18	87021	BELVEDERE MARITTIMO	PRIVATE
1325	180	CALABRIA	180027	CASA DI CURA CASCINI SRL	PIAZZA DE SETA N 2	87021	BELVEDERE MARITTIMO	PRIVATE
1326	180	CALABRIA	180028	SACRO CUORE	CORSO D`ITALIA 50	87100	COSENZA	PRIVATE
1327	180	CALABRIA	180029	CASA DI CURA SCARNATI	VIA ZARA,4	87100	COSENZA	PRIVATE
1328	180	CALABRIA	180030	CASA DI CURA VILLA DEGLI OLEANDRI	C.DA PASQUALI,117	87040	MENDICINO	PRIVATE
1329	180	CALABRIA	180042	CASA DI CURA MADONNA DELLA CATENA	VIA FRA` BENEDETTO,33	87040	DIPIGNANO	PRIVATE
1330	180	CALABRIA	180079	OSPEDALE DI SAN MARCO ARGENTANO	VIA NEGRONI	87018	SAN MARCO ARGENTANO	PUBLIC
1331	180	CALABRIA	180081	CASA DI CURA M.MISASI GR.S.BARTOLO	P.ZZA CRISPI 6	87100	COSENZA	PRIVATE
1332	180	CALABRIA	180083	CASA DI CURA SAN LUCA SRL	LOC.FORTINO N 334 FABBRICATO D10	87028	PRAIA A MARE	PRIVATE
1333	180	CALABRIA	180084	MEDICAL HOTEL CLIMAT. SPES PIETR.'ARENA'	VIA NAZIONALE 70	87020	SANGINETO	PRIVATE
1334	180	CALABRIA	180086	CASA DI CURA SAN FRANCESCO	VIA CANDELISI 105	87040	MENDICINO	PRIVATE
1335	180	CALABRIA	180100	'VILLA TORANO'	CONTRADA MACCHIA TAVOLA	87010	TORANO CASTELLO	PRIVATE
1336	180	CALABRIA	180916	INRCA	C.DA MUOIO PICCOLO	87100	COSENZA	PUBLIC
1337	180	CALABRIA	180032	OSPEDALE CIVILE SAN GIOVANNI DI DIO	VIA BOLOGNA	88900	CROTONE	PUBLIC
1338	180	CALABRIA	180044	5CASA DI CURA 'VILLA GIOSE'	VIA TUFOLO	88900	CROTONE	PRIVATE
1339	180	CALABRIA	180045	CASA DI CURA S.RITA DOTT. CAPARRA	VIA ROMA 227	88811	BELVEDERE DI SPINELLO	PRIVATE
1340	180	CALABRIA	180046	CASA DI CURA REUMATOLOGICA OLIVETI	VIA laghi silani	88836	COTRONEI	PRIVATE
1341	180	CALABRIA	180054	ISTITUTO SANT`ANNA	VIA PER CAPOCOLONNA	88900	CROTONE	PRIVATE
1342	180	CALABRIA	180085	ROMOLO HOSPITAL (EX VILLA EVA)	VIA SANDRO PERTINI (LOCALITA` CUPONE)	88821	ROCCA DI NETO	PRIVATE
1343	180	CALABRIA	180101	CALABRODENTAL SRL	VIA ENRICO FERMI	88900	CROTONE	PRIVATE
1344	180	CALABRIA	180033	OSPEDALE DI LAMEZIA TERME	VIA ARTURO PERUGINI	88046	LAMEZIA TERME	PUBLIC
1345	180	CALABRIA	180040	OSPEDALE DI SOVERIA MANNELLI	VIALE RUBBETTINO	88049	SOVERIA MANNELLI	PUBLIC
1346	180	CALABRIA	180041	OSPEDALE SAN BIAGIO	VIA MARIO CERAVOLO	88064	CHIARAVALLE CENTRALE	PUBLIC
1347	180	CALABRIA	180043	CASA DI CURA VILLA DEL SOLE	VIALE PIO X, 202	88100	CATANZARO	PRIVATE
1348	180	CALABRIA	180048	CASA DI CURA VILLA NUCCIA	VIA PADULA, 1	88100	CATANZARO	PRIVATE
1349	180	CALABRIA	180049	CASA DI CURA S.VINCENZO	VIA BARLAAMDA SEMINARA N[26	88100	CATANZARO	PRIVATE
1350	180	CALABRIA	180050	CASA DI CURA VILLA SERENA	VIA LUIGI PASCALI, 11	88100	CATANZARO	PRIVATE
1351	180	CALABRIA	180051	CASA DI CURA PRIVATA VILLA MICHELINO SRL	VIA DUCA D`AOSTA,164	88046	LAMEZIA TERME	PRIVATE
1352	180	CALABRIA	180052	CASA DI CURA VILLA PUCA	VIA TOMMASO CAMPANELLA 60	88100	CATANZARO	PRIVATE
1353	180	CALABRIA	180053	CASA DI CURA SANT`ANNA HOSPITAL	VIALE PIO X 111	88100	CATANZARO	PRIVATE
1354	180	CALABRIA	180055	OSPEDALE BASSO IONIO	VIA DON CARLO DE CARDONA	88068	SOVERATO	PUBLIC
1355	180	CALABRIA	180034	P.O. JAZZOLINO	PIAZZA FLEMING	89900	VIBO VALENTIA	PUBLIC
1356	180	CALABRIA	180035	P.O. TROPEA	VIA PROVINCIALE	89861	TROPEA	PUBLIC
1357	180	CALABRIA	180047	VILLA DEI GERANI	VIA S. DOMENICO SAVIO,10	89900	VIBO VALENTIA	PRIVATE
1358	180	CALABRIA	180056	P.O. SORIANO CALABRO	VIA AMENDOLA	89831	SORIANO CALABRO	PUBLIC
1359	180	CALABRIA	180057	P.O. SERRA SAN BRUNO	VIA ALFONSO SCRIVO	89822	SERRA SAN BRUNO	PUBLIC
1360	180	CALABRIA	180060	P.O. 'F. PENTIMALLI'	VIA BRUNO BUOZZI	89015	PALMI	PUBLIC
1361	180	CALABRIA	180061	P.O. 'S. MARIA DEGLI UNGHERESI'	VIA MONTEGRAPPA	89024	POLISTENA	PUBLIC
1362	180	CALABRIA	180062	OSPEDALE 'TIBERIO EVOLI' - MELITO P.S.	CORSO GARIBALDI	89063	MELITO DI PORTO SALVO	PUBLIC
1363	180	CALABRIA	180063	P.O. 'MARIA PIA DI SAVOIA'	CORSO ASPROMONTE	89014	OPPIDO MAMERTINA	PUBLIC
1364	180	CALABRIA	180064	P.O. 'PRINCIPESSA DI PIEMONTE'	VIA PRINCIPESSA DI PIEMONTE	89029	TAURIANOVA	PUBLIC
1365	180	CALABRIA	180067	OSPEDALE 'SCILLESI D`AMERICA' - SCILLA	VIA TRIPI SUPERIORE	89058	SCILLA	PUBLIC

1366	180	CALABRIA	180068	P.O. 'GIOVANNI XXIII'	VIA MADAME CURIE	89013	GIOIA TAURO	PUBLIC
1367	180	CALABRIA	180071	ISTITUTO ORTOPEDICO MEZZOGIORNO D`ITALIA	VIA EREMO, 10	89100	REGGIO DI CALABRIA	PRIVATE
1368	180	CALABRIA	180073	CASA DI CURA 'VILLA AURORA'	VIA AURORA	89100	REGGIO DI CALABRIA	PRIVATE
1369	180	CALABRIA	180074	POLICLINICO 'MADONNA DELLA CONSOLAZIONE'	VIA CARINALE PORTANOVA, 126	89100	REGGIO DI CALABRIA	PRIVATE
1370	180	CALABRIA	180075	CASA DI CURA 'VILLA CAMINITI'	VIA NAZIONALE, 421	89018	VILLA SAN GIOVANNI	PRIVATE
1371	180	CALABRIA	180076	CASA DI CURA 'VILLA S.ANNA'	VIA CROCIFISSO, 25	89100	REGGIO DI CALABRIA	PRIVATE
1372	180	CALABRIA	180077	CASA DI CURA 'VILLA ELISA' S.P.A.	VIA VITTORIO VENETO, 141	89021	CINQUEFRONDI	PRIVATE
1373	180	CALABRIA	180912	AZIENDA OSPEDALIERA DI COSENZA	VIA SAN MARTINO	87100	COSENZA	PUBLIC
1374	180	CALABRIA	180912	AZIENDA OSPEDALIERA DI COSENZA	VIA SAN MARTINO	87100	COSENZA	PUBLIC
1375	180	CALABRIA	180912	AZIENDA OSPEDALIERA DI COSENZA	VIA SAN MARTINO	87100	COSENZA	PUBLIC
1376	180	CALABRIA	180913	AZIENDA OSPEDALIERA PUGLIESE CIACCIO	VIALE PIO X	88100	CATANZARO	PUBLIC
1377	180	CALABRIA	180913	AZIENDA OSPEDALIERA PUGLIESE CIACCIO	VIALE PIO X	88100	CATANZARO	PUBLIC
1378	180	CALABRIA	180913	AZIENDA OSPEDALIERA PUGLIESE CIACCIO	VIALE PIO X	88100	CATANZARO	PUBLIC
1379	180	CALABRIA	180914	AZIENDA OSPEDALIERA MATER DOMINI	VIALE EUROPA LOC. GERMANTO	88100	CATANZARO	PUBLIC
1380	180	CALABRIA	180915	OSPEDALE BIANCHI - MELACRINO - MORELLI	VIA PROVINCIALE SPIRITO SANTO, 24	89100	REGGIO DI CALABRIA	PUBLIC
1381	190	SICILIA	190112	P.O. F.LLI PARLAPIANO - RIBERA	VIA CIRCONVALLAZIONE	92016	RIBERA	PUBLIC
1382	190	SICILIA	190115	P.O. BARONE LOMBARDO - CANICATTI`	CONTRADA GIARRE	92024	CANICATTI`	PUBLIC
1383	190	SICILIA	190116	P.O. S. GIACOMO D'ALTOPASSO LICATA	C/DA CANNAVECCHIA	92027	LICATA	PUBLIC
1384	190	SICILIA	190503	CASA DI SALUTE IGNAZIO ATTARDI S.P.A.	VIA NAZIONALE 16	92020	SANTO STEFANO QUISQUINA	PRIVATE
1385	190	SICILIA	190504	SIA CASA DI CURA S. ANNA S.R.L.	VIA PORTA AUREA	92100	AGRIGENTO	PRIVATE
1386	190	SICILIA	190117	P.O.M.RAIMONDI	VIA FORLANINI 5	93017	SAN CATALDO	PUBLIC
1387	190	SICILIA	190118	P.O. MARIA IMMACOLATA LONGO	VIA DOGLIOTTI S.N.	93014	MUSSOMELI	PUBLIC
1388	190	SICILIA	190124	P.O. S. STEFANO	VIA DANTE 2	93013	MAZZARINO	PUBLIC
1389	190	SICILIA	190125	P.O.SUOR CECILIA BASAROCCO	PIAZZA MARTIRI DI NASSIRYA	93015	NISCEMI	PUBLIC
1390	190	SICILIA	190505	CASA DI CURA 'REGINA PACIS'	VIA SCALEA 3/5	93017	SAN CATALDO	PRIVATE
1391	190	SICILIA	190506	SO.GE.SA.SPA.C.DI.C.S.BARBARA	VIA MINERBIO 3	93012	GELA	PRIVATE
1392	190	SICILIA	190149	P.O.BASSO RAGUSA M.- MILITELLO	V.LE REGINA MARGHERITA 25	95043	MILITELLO IN VAL DI CATANIA	PUBLIC
1393	190	SICILIA	190151	P.O.SS.SALVATORE - PATERNO'	VIA LIVORNO	95047	PATERNO`	PUBLIC
1394	190	SICILIA	190152	P.O.M.SS.ADDOLORATA-BIANCAVILLA	VIA CRISTOFORO COLOMBO 77	95033	BIANCAVILLA	PUBLIC
1395	190	SICILIA	190162	P.O.S. MARTA S.VENERA - ACIREALE	VIA CARONIA S.N.	95024	ACIREALE	PUBLIC
1396	190	SICILIA	190163	P.O.S.GIOVANNI DI DIO E S.ISODORO-GIARRE	VIA C. FORLANINI CONTRADA COSTE	95014	GIARRE	PUBLIC
1397	190	SICILIA	190165	P.O.CASTIGLIONE PRESTIANNI - BRONTE	VIA UMBERTO 406	95034	BRONTE	PUBLIC
1398	190	SICILIA	190511	CASA DI CURA DI STEFANO VELONA	VIA S. EUPLIO 162	95124	CATANIA	PRIVATE
1399	190	SICILIA	190512	CASA DI CURA MADONNA DEL ROSARIO	VIA BRONTE 44	95125	CATANIA	PRIVATE
1400	190	SICILIA	190513	CASA DI CURA G.B. MORGAGNI S.R.L.	VIA DEL BOSCO 105	95125	CATANIA	PRIVATE
1401	190	SICILIA	190515	CASA DI CURA S. RITA SRL	VIA DOTT. CONSOLI 49	95124	CATANIA	PRIVATE
1402	190	SICILIA	190516	MATER DEI DI G.NESI & C. S.P.A	VIALE MARIO RAPISARDI 1	95124	CATANIA	PRIVATE
1403	190	SICILIA	190517	CASA DI CURA VILLA DEI GERANI SrL	VIII[STRADA ZONA INDUSTRIALE	95121	CATANIA	PRIVATE
1404	190	SICILIA	190518	CASA DI CURA S. VITO SRL	VIA S. VITO 19	95121	CATANIA	PRIVATE
1405	190	SICILIA	190519	HUMANITAS CENTRO CATANESE DI ONCOLOGIA	VIA V.E. DA BORMIDA 64	95126	CATANIA	PRIVATE
1406	190	SICILIA	190520	CASA CURA CENTRO CATANESE MED. E CH.	VIA BATTELLO 48	95126	CATANIA	PRIVATE
1407	190	SICILIA	190521	CASA DI CURA CARMIDE	VIA FEUDO GRANDE 13	95126	CATANIA	PRIVATE
1408	190	SICILIA	190522	CASA DI CURA LANTERI VILLA FIORITA	VIA REGINA BIANCA 75	95126	CATANIA	PRIVATE
1409	190	SICILIA	190523	CASA DI CURA GIBIINO SRL	VIALE ODORICO DA PORDENONE 25	95128	CATANIA	PRIVATE
1410	190	SICILIA	190553	CASA DI CURA RIABILITATIVA VILLA SOFIA	VIE DELLE TERME , 80	95024	ACIREALE	PRIVATE
1411	190	SICILIA	190554	ISTITUTO ONCOLOGICO DEL MEDITERRANEO SPA	VIA PENNINAZZO 7	95029	VIAGRANDE	PRIVATE
1412	190	SICILIA	190703	CASA DI CURA CAUDULLO SRL	VIALE MARIO RAPISARDI N. 560	95100	CATANIA	PRIVATE
1413	190	SICILIA	190709	CASA DI CURA CLIN.BASILE GEST.TIGANO SRL	V.LE ODORICO DA PORDENONE 1	95128	CATANIA	PRIVATE
1414	190	SICILIA	190710	CASA DI CURA PROF.E.FALCIDIA SrL	V.LE ODORICO DA PORDENONE 32/34	95126	CATANIA	PRIVATE
1415	190	SICILIA	190711	CASA DI CURA LUCINA	VIA FEDERICO DE ROBERTO 30	95121	CATANIA	PRIVATE
1416	190	SICILIA	190712	CASA DI CURA MUSUMECI GECAS SRL	CORSO ITALIA 127	95127	CATANIA	PRIVATE
1417	190	SICILIA	190715	CASA DI CURA VILLA S.FRANCESCO	V.LE ODORICO DA PORDENONE 46/48	95126	CATANIA	PRIVATE
1418	190	SICILIA	190719	CASA DI CURA GRETTER GEST.C.M.C SRL	VIA MANCINI BATTAGLIA 5	95100	CATANIA	PRIVATE
1419	190	SICILIA	190723	CASA DI CURA VALSALVA AURORA SrL	V.LE A. USODIMARE	95123	CATANIA	PRIVATE
1420	190	SICILIA	190724	ISCAS MORGAGNI NORD SRL	VIA DELLA RESISTENZA 31	95030	PEDARA	PRIVATE
1421	190	SICILIA	190725	CASA DI CURA ARGENTO	VIA OTTAVIO D`ARCANGELO 16	95123	CATANIA	PRIVATE
1422	190	SICILIA	190126	OSPEDALE 'CARLO BASILOTTA'	VIA S.GIOVANNI	94014	NICOSIA	PUBLIC
1423	190	SICILIA	190128	OSPEDALE 'FERRO-CAPRA-BRANCIFORTE'	CONTRADA S. GIOVANNI	94012	LEONFORTE	PUBLIC
1424	190	SICILIA	190129	OSPEDALE 'CHIELLO'	C.DA BELLIA	94015	PIAZZA ARMERINA	PUBLIC
1425	190	SICILIA	190167	P.O. 'SAN VINCENZO' TAORMINA	CONTRADA SIRINA	98039	TAORMINA	PUBLIC
1426	190	SICILIA	190171	P. O. 'G. FOGLIANI' MILAZZO	VILLAGGIO GRAZIA	98057	MILAZZO	PUBLIC
1427	190	SICILIA	190172	OSPEDALE LIPARI	VIA S.ANNA	98055	LIPARI	PUBLIC
1428	190	SICILIA	190174	P.O.'NUOVO CUTRONI ZODDA' BARCELLONA	CTR.DA S. ANDREA	98051	BARCELLONA POZZO DI GOTTO	PUBLIC

1429	190	SICILIA	190175	P.O. ' BARONE ROMEO' PATTI	VIA MAZZINI	98066	PATTI	PUBLIC
1430	190	SICILIA	190177	OSPEDALE SS. SALVATORE - MISTRETTA	VIA ANNA SALAMONE	98073	MISTRETTA	PUBLIC
1431	190	SICILIA	190178	P.O. S. AGATA MILITELLO	VIA MEDICI	98076	SANT`AGATA DI MILITELLO	PUBLIC
1432	190	SICILIA	190524	I.O.M.I. F.SCALABRINO GANZIRRI	VIA CONS POMPEA 360 GANZIRRI MESSINA	98165	MESSINA	PRIVATE
1433	190	SICILIA	190525	CASA DI CURA S.CAMILLO	VIALE P.UMBERTO N.71 MESSINA	98122	MESSINA	PRIVATE
1434	190	SICILIA	190526	CASA DI CURA CRISTO RE	VIALE P.UMBERTO N.89 MESSINA	98122	MESSINA	PRIVATE
1435	190	SICILIA	190527	CASA DI CURA CARMONA SRL	VIALE P.UMBERTO N.75/G	98122	MESSINA	PRIVATE
1436	190	SICILIA	190528	CASA DI CURA VILLA SALUS S.A.S.	VIALE REG.MARGHERITA N. 15/B MESSINA	98121	MESSINA	PRIVATE
1437	190	SICILIA	190529	CASA DI CURA S.RITA ATI HOSPITAL SRL	VIA COLAPESCE N.32	98121	MESSINA	PRIVATE
1438	190	SICILIA	190530	C.O.T. S.P.A. (CURE ORTOPED. TRAUM.)	VIA DUCEZIO N.1	98124	MESSINA	PRIVATE
1439	190	SICILIA	190531	CASA DI CURA VILLA IGEA SRL	VIA CONSOLARE VALERIA N 47 MESSINA	98124	MESSINA	PRIVATE
1440	190	SICILIA	190601	CENTRO CHIRURGICO VILLA MARIA S.R.L.	VIA NAZIONALE - ORTOLIUZZO KM. 32	98100	MESSINA	PRIVATE
1441	190	SICILIA	190716	CASA DI CURA CAPPELLANI S.R.L.	VIALE REG.ELENA N.335	98121	MESSINA	PRIVATE
1442	190	SICILIA	190179	FOND. IST. S. RAFFAELE - GIGLIO	C.DA PIETRAPOLLASTRA	90015	CEFALU`	PUBLIC
1443	190	SICILIA	190180	P.O. MADONNA DELL` ALTO	C.DA S. ELIA	90027	PETRALIA SOTTANA	PUBLIC
1444	190	SICILIA	190181	OSPEDALE PUBBLICO S. CIMINO	VIA SALVATORE CIMINO	90018	TERMINI IMERESE	PUBLIC
1445	190	SICILIA	190182	OSPEDALE DEI BIANCHI V. EMANUELE	VIA DON G.COLLETTO	90034	CORLEONE	PUBLIC
1446	190	SICILIA	190183	OSP.REGINA MARGHERITA	VIA FRANCESCO CRISPI 99	90030	PALAZZO ADRIANO	PUBLIC
1447	190	SICILIA	190184	OSPEDALE CIVICO - PARTINICO	VIA CIRCONVALLAZIONE, 1 PARTINICO	90047	PARTINICO	PUBLIC
1448	190	SICILIA	190190	OSPEDALE G. F. INGRASSIA	CORSO CALATAFIMI 1002	90142	PALERMO	PUBLIC
1449	190	SICILIA	190193	CASA DEL SOLE LANZA DI TRABIA	VIA UR3 N.19	90136	PALERMO	PUBLIC
1450	190	SICILIA	190195	OSPEDALE ORTOPEDICO ENRICO ALBANESE	VIA PAPA SERGIO I[, N.5	90142	PALERMO	PUBLIC
1451	190	SICILIA	190198	EX P.O. GUADAGNA	VIA VILLAGRAZIA,46	90146	PALERMO	PUBLIC
1452	190	SICILIA	190199	OSP. BUCCHERI LA FERLA FATEBENEFRATELLI	VIA M. MARINE 197	90123	PALERMO	PUBLIC
1453	190	SICILIA	190532	CASA DI CURA IGEA S.N.C.	VIA ROMA 193	90047	PARTINICO	PRIVATE
1454	190	SICILIA	190533	CASA DI CURA CANDELA SPA	VIA VILLAREALE 54	90141	PALERMO	PRIVATE
1455	190	SICILIA	190534	CASA DI CURE ORESTANO S.R.L.	VIA PIETRO D`ASARO, N 48	90130	PALERMO	PRIVATE
1456	190	SICILIA	190536	CASA DI CURA 'TRIOLO ZANCLA' S.P.A.	P.ZZA FONDERIA N.23	90133	PALERMO	PRIVATE
1457	190	SICILIA	190537	CASA DI CURA SERENA S.P.A.	VIA DELLA REGIONE SICILIANA 1470	90135	PALERMO	PRIVATE
1458	190	SICILIA	190538	CASA DI CURA NOTO PASQUALINO S.R.L.	VIA DANTE 330	90141	PALERMO	PRIVATE
1459	190	SICILIA	190539	CASA DI CURA D`ANNA - PALERMO	VIA ALTOFONTE PORRAZZI, 81	90129	PALERMO	PRIVATE
1460	190	SICILIA	190540	NUOVA CASA DI CURA DEMMA	VIALE REGINA MARGHERITA 5	90138	PALERMO	PRIVATE
1461	190	SICILIA	190541	CASA DI CURA MACCHIARELLA S.P.A.	VIALE REGINA MARGHERITA 25	90138	PALERMO	PRIVATE
1462	190	SICILIA	190542	CASA DI CURA TORINA	VIA F. SPALLITTA N.18	90145	PALERMO	PRIVATE
1463	190	SICILIA	190543	CASA DI CURA VILLA MARGHERITA	VIA MARCHESE DI VILLABIANCA, 6	90143	PALERMO	PRIVATE
1464	190	SICILIA	190544	CASA DI CURA STAGNO S.R.L.	VIA SAN LORENZO COLLI, 316	90146	PALERMO	PRIVATE
1465	190	SICILIA	190545	CASA DI CURE COSENTINO S.R.L.	CORSO DEI MILLE 56	90123	PALERMO	PRIVATE
1466	190	SICILIA	190551	CASA DI CURA LA MADDALENA S.P.A.	VIA S. LORENZO COLLI, 312/D	90146	PALERMO	PRIVATE
1467	190	SICILIA	190602	CENTRO ANDROS S.R.L.	VIA AUSONIA 43/45	90144	PALERMO	PRIVATE
1468	190	SICILIA	190603	CENTRO DI CHIRURGIA GENESI SRL	VIA P. P. VASTA , 2	90144	PALERMO	PRIVATE
1469	190	SICILIA	190718	CASA DI CURA LATTERI S.R.L.	VIA F. CORDOVA, 62	90143	PALERMO	PRIVATE
1470	190	SICILIA	190720	CASA DI CURA VILLA MARIA ELEONORA	VIALE REGIONE SICILIANA, 1571	90135	PALERMO	PRIVATE
1471	190	SICILIA	190131	OSPEDALE R. GUZZARDI VITTORIA	VIA PAPA GIOVANNI XXIII	97019	VITTORIA	PUBLIC
1472	190	SICILIA	190132	OSPEDALE REGINA MARGHERITA COMISO	C/DA MASTRELLA	97013	COMISO	PUBLIC
1473	190	SICILIA	190136	OSPEDALE MAGGIORE	VIA RESISTENZA PARTIGIANA	97015	MODICA	PUBLIC
1474	190	SICILIA	190137	OSPEDALE BUSACCA - SCICLI	VIA OSPEDALE	97018	SCICLI	PUBLIC
1475	190	SICILIA	190552	CLINICA DEL MEDITERRANEO S.R.L.	VIA ETTORE FIERAMOSCA N[100	97100	RAGUSA	PRIVATE
1476	190	SICILIA	190138	OSPEDALE 'G.DI MARIA'	CONTRADA CHIUSA DI CARLO	96012	AVOLA	PUBLIC
1477	190	SICILIA	190139	OSPEDALE 'TRIGONA'	VIA DEI MILLE 98	96017	NOTO	PUBLIC
1478	190	SICILIA	190142	OSPEDALE E MUSCATELLO	CTR. GRANATELLO	96011	AUGUSTA	PUBLIC
1479	190	SICILIA	190143	OSPEDALE GENERALE DI ZONA	VIA OSPEDALE	96016	LENTINI	PUBLIC
1480	190	SICILIA	190507	CASA DI CURA SANTA LUCIA GLEF	VIA LOMBARDIA 1	96100	SIRACUSA	PRIVATE
1481	190	SICILIA	190508	CASA DI CURA VILLA MAURITIUS ARC	VIA FRANCOFONTE 5	96100	SIRACUSA	PRIVATE
1482	190	SICILIA	190509	IST.ORT. VILLA SALUS DI I. GALATIOTO SRL	VIA PROVINC. PER BRUCOLI 507/A	96011	AUGUSTA	PRIVATE
1483	190	SICILIA	190550	C. DI CURA 'VILLA AZZURRA'	TRAV. VIA SCALA GRECA, 24	96100	SIRACUSA	PRIVATE
1484	190	SICILIA	190701	NUOVA CLINICA VILLA RIZZO	VIA S.AGATI N 3	96100	SIRACUSA	PRIVATE
1485	190	SICILIA	190103	P.O. 'B. NAGAR' PANTELLERIA	PIAZZALE NICOLO` ALMANZA	91017	PANTELLERIA	PUBLIC
1486	190	SICILIA	190104	P.O. SAN BIAGIO MARSALA	PIAZZA SAN FRANCESCO, 1	91025	MARSALA	PUBLIC
1487	190	SICILIA	190105	P.O.ABELE AJELLO	VIA SALEMI n° 175	91026	MAZARA DEL VALLO	PUBLIC
1488	190	SICILIA	190106	P.O. VITTORIO EMANUELE III SALEMI	VIA DANTE ALIGHIERI	91018	SALEMI	PUBLIC
1489	190	SICILIA	190107	P:O. CIVILE V.EMANUELE II CASTELVETRANO	VIA MARINELLA - CASTELVETRANO	91022	CASTELVETRANO	PUBLIC
1490	190	SICILIA	190108	OSPEDALE SAN VITO E SANTO SPIRITO	VIA FRANCESCO CRISPI 116	91011	ALCAMO	PUBLIC
1491	190	SICILIA	190501	CASA DI CURA VILLA DEI GERANI	VIA A. MANZONI, 83 CASA SANTA ERICE	91016	ERICE	PRIVATE

1492	190	SICILIA	190502	CASA DI CURA SANT`ANNA SRL.	VIA SANT`ANNA, 38 ERICE C.S.	91016	ERICE	PRIVATE
1493	190	SICILIA	190549	CASA DI CURA MORANA SRL	CONTRADA DARA 744/D	91025	MARSALA	PRIVATE
1494	190	SICILIA	190901	A.R.N.A.S. OSPED CIVICO DI CRISTINA ASCO	PIAZZA NICOLA LEOTTA, 2	90127	PALERMO	PUBLIC
1495	190	SICILIA	190901	A.R.N.A.S. OSPED CIVICO DI CRISTINA ASCO	PIAZZA NICOLA LEOTTA, 2	90127	PALERMO	PUBLIC
1496	190	SICILIA	190901	A.R.N.A.S. OSPED CIVICO DI CRISTINA ASCO	PIAZZA NICOLA LEOTTA, 2	90127	PALERMO	PUBLIC
1497	190	SICILIA	190901	A.R.N.A.S. OSPED CIVICO DI CRISTINA ASCO	PIAZZA NICOLA LEOTTA, 2	90127	PALERMO	PUBLIC
1498	190	SICILIA	190902	AZIENDA OSPEDALIERA GARIBALDI	PIAZZA SANTA MARIA DI GESU', 5/7	95124	CATANIA	PUBLIC
1499	190	SICILIA	190902	AZIENDA OSPEDALIERA GARIBALDI	PIAZZA SANTA MARIA DI GESU', 5/7	95124	CATANIA	PUBLIC
1500	190	SICILIA	190902	AZIENDA OSPEDALIERA GARIBALDI	PIAZZA SANTA MARIA DI GESU', 5/7	95124	CATANIA	PUBLIC
1501	190	SICILIA	190902	AZIENDA OSPEDALIERA GARIBALDI	PIAZZA SANTA MARIA DI GESU', 5/7	95124	CATANIA	PUBLIC
1502	190	SICILIA	190903	AZ. OSP. UNIV. V. EMAN. FERR. S.BAMBINO	VIA G. CLEMENTI N. 36	95124	CATANIA	PUBLIC
1503	190	SICILIA	190903	AZ. OSP. UNIV. V. EMAN. FERR. S.BAMBINO	VIA G. CLEMENTI N. 36	95124	CATANIA	PUBLIC
1504	190	SICILIA	190904	AZIENDA OSPEDALIERA S. ELIA	VIA L.RUSSO N[6	93100	CALTANISSETTA	PUBLIC
1505	190	SICILIA	190905	AZIENDA OSPEDALIERA CANNIZZARO - CATANIA	VIA MESSINA 829	95126	CATANIA	PUBLIC
1506	190	SICILIA	190906	AZIENDA OSPEDALIERA PAPARDO	CONTRADA SPERONE	98158	MESSINA	PUBLIC
1507	190	SICILIA	190907	AZIENDA OSPEDALIARA VILLA SOFIA CTO	VIALE STRASBURGO, 233	90146	PALERMO	PUBLIC
1508	190	SICILIA	190907	AZIENDA OSPEDALIARA VILLA SOFIA CTO	VIALE STRASBURGO, 233	90146	PALERMO	PUBLIC
1509	190	SICILIA	190907	AZIENDA OSPEDALIARA VILLA SOFIA CTO	VIALE STRASBURGO, 233	90146	PALERMO	PUBLIC
1510	190	SICILIA	190908	AZ.OSPEDALIERA S.GIOVANNI DI DIO	C.DA CONSOLIDA	92100	AGRIGENTO	PUBLIC
1511	190	SICILIA	190909	AZ. OSP.'GRAVINA E S.PIETRO' CALTAGIRONE	VIA PORTOSALVO N[7	95041	CALTAGIRONE	PUBLIC
1512	190	SICILIA	190910	AZIENDA OSPEDALIERA UMBERTO I ENNA	c.da ferrante	94100	ENNA	PUBLIC
1513	190	SICILIA	190911	AZIENDA OSPEDALIERA VITTORIO EMANUELE	VIA PALAZZI 171	93012	GELA	PUBLIC
1514	190	SICILIA	190912	AZIENDA OSPEDALIERA PIEMONTE	VIALE EUROPA	98100	MESSINA	PUBLIC
1515	190	SICILIA	190913	A.O. 'V. CERVELLO'	VIA TRABUCCO, 180	90146	PALERMO	PUBLIC
1516	190	SICILIA	190914	OSPEDALE 'CIVILE MARIA PATERNO` AREZZO	VIA G.D.VITTORIO 51	97100	RAGUSA	PUBLIC
1517	190	SICILIA	190915	AZIENDA OSPEDALIERA UMBERTO I	VIA TESTAFERRATA 1	96100	SIRACUSA	PUBLIC
1518	190	SICILIA	190915	AZIENDA OSPEDALIERA UMBERTO I	VIA TESTAFERRATA 1	96100	SIRACUSA	PUBLIC
1519	190	SICILIA	190916	A.O. S.ANTONIO ABATE	VIA COSENZA - RAGANZILI	91016	ERICE	PUBLIC
1520	190	SICILIA	190917	A.O.'OSPEDALI CIVILI RIUNITI'SCIACCA	VIA POMPEI CONTRADA SENIAZZA SCIACC	92019	SCIACCA	PUBLIC
1521	190	SICILIA	190920	AZIENDA OSPEDALIERA UNIVERSITARIA PALERM	VIA DEL VESPRO	90127	PALERMO	PUBLIC
1522	190	SICILIA	190930	AZIENDA OSP. UNIV. DI MESSINA	VIALE GAZZI	98124	MESSINA	PUBLIC
1523	190	SICILIA	190940	AZIENDA POLICLINICO UNIV. DI CATANIA	VIA S. SOFIA , 78	95123	CATANIA	PUBLIC
1524	190	SICILIA	190950	ASSOCIAZIONE OASI MARIA SS	VIA CONTE RUGGERO 73	94018	TROINA	PUBLIC
1525	190	SICILIA	190960	IRCCS Centro Neurolesi 'Bonino Pulejo'	s.s. 113 Contrada Casazza	98124	MESSINA	PUBLIC
1526	200	SARDEGNA	200001	OSPEDALE CIVILE SASSARI	VIA DE NICOLA 14	07100	SASSARI	PUBLIC
1527	200	SARDEGNA	200002	OSPEDALE CIVILE ALGHERO	VIA DON MINZONI	07041	ALGHERO	PUBLIC
1528	200	SARDEGNA	200003	OSPEDALE A. SEGNI OZIERI	VIA CAPUCCINI	07014	OZIERI	PUBLIC
1529	200	SARDEGNA	200004	OSPEDALE CIVILE G.A. ALIVESI ITTIRI	VIA OSPEDALE	07044	ITTIRI	PUBLIC
1530	200	SARDEGNA	200005	OSPEDALE CIVILE THIESI	VIALE SEUNIS	07041	THIESI	PUBLIC
1531	200	SARDEGNA	200008	OSPEDALE MARINO REGINA MARGHERITA ALGHER	VIALE I MAGGIO 1	07041	ALGHERO	PUBLIC
1532	200	SARDEGNA	200013	POLICLINICO SASSARESE SPA	VIALE ITALIA 11	07100	SASSARI	PRIVATE
1533	200	SARDEGNA	200006	P.O. PAOLO DETTORI - TEMPIO P.	VIA GRAZIA DELEDDA	07019	TEMPIO PAUSANIA	PUBLIC
1534	200	SARDEGNA	200011	P.O. PAOLO MERLO LA MADDALENA	VIA AMM. MAGNAGHI	07024	LA MADDALENA	PUBLIC
1535	200	SARDEGNA	200012	P.O. SAN GIOVANNI DI DIO OLBIA	VIA CADUTI DEL LAVORO, 35	07026	OLBIA	PUBLIC
1536	200	SARDEGNA	200017	P.O. SAN FRANCESCO	VIA MANNIRONI	08100	NUORO	PUBLIC
1537	200	SARDEGNA	200018	P.O. C. Zonchello	Piazza Sardegna 1	08100	NUORO	PUBLIC
1538	200	SARDEGNA	200020	P.O. San Camillo	Corso IV Novembre	08038	SORGONO	PUBLIC
1539	200	SARDEGNA	200019	P.O. 'NOSTRA SIGNORA DELLA MERCEDE'	VIA OSPEDALE 1	08045	LANUSEI	PUBLIC
1540	200	SARDEGNA	200023	CASA DI CURA M. TOMMASINI s.r.l.	Via Ospedale Ierzu	08044	JERZU	PRIVATE
1541	200	SARDEGNA	200021	P. OSPEDALIERO 'A.G. MASTINO' - BOSA	VIA G.A. PISCHEDDA	08013	BOSA	PUBLIC
1542	200	SARDEGNA	200052	P. OSPEDALIERO 'SAN MARTINO' - ORISTANO	VIA ROCKEFELLER	09170	ORISTANO	PUBLIC
1543	200	SARDEGNA	200053	P. OSPEDALIERO 'G.P. DELOGU' - GHILARZA	CORSO UMBERTO N° 176	09074	GHILARZA	PUBLIC
1544	200	SARDEGNA	200054	C.C. 'MADONNA DEL RIMEDIO' - ORISTANO	VIA GIOTTO N° 6	09170	ORISTANO	PRIVATE
1545	200	SARDEGNA	200034	PRESIDIO OSPEDALIERO N.S. DI BONARIA	VIA ROMA SNC	09038	SAN GAVINO MONREALE	PUBLIC
1546	200	SARDEGNA	200028	P.O.SIRAI	LOCALITA' SIRAI	09013	CARBONIA	PUBLIC
1547	200	SARDEGNA	200029	P.O.SANTA BARBARA	VIA SAN LEONARDO 1	09016	IGLESIAS	PUBLIC
1548	200	SARDEGNA	200030	P.O.CTO	VIA CATTANEO	09016	IGLESIAS	PUBLIC
1549	200	SARDEGNA	200033	P.O.F.LLI CROBU	LOCALITA' CANONICA	09016	IGLESIAS	PUBLIC
1550	200	SARDEGNA	200022	P.O. S.GIUSEPPE	VIA EMILIA 1	08033	ISILI	PUBLIC
1551	200	SARDEGNA	200025	P.O. R. BINAGHI	VIA IS GUADAZZONIS 2	09126	CAGLIARI	PUBLIC
1552	200	SARDEGNA	200026	P.O. MARINO	VIALE POETTO	09126	CAGLIARI	PUBLIC
1553	200	SARDEGNA	200031	P.O. SS. TRINITA'	VIA IS MIRRIONIS 92	09121	CAGLIARI	PUBLIC
1554	200	SARDEGNA	200035	P.O. SAN MARCELLINO	VIALE RINASCITA 2	09043	MURAVERA	PUBLIC

1555	200	SARDEGNA	200036	P.O. A. BUSINCO	VIA JENNER	09121	CAGLIARI	PUBLIC
1556	200	SARDEGNA	200038	P.O. MICROCITEMICO	VIA JENNER	09121	CAGLIARI	PUBLIC
1557	200	SARDEGNA	200039	CASA DI CURA LAY S.p.A.	VIALE S. IGNAZIO DA LACONI 34	09123	CAGLIARI	PRIVATE
1558	200	SARDEGNA	200040	CASA DI CURA S.ANNA s.r.l.	VIA LA VEGA 9	09127	CAGLIARI	PRIVATE
1559	200	SARDEGNA	200041	CASA DI CURA CITTA' DI QUARTU s.r.l.	VIA SILESU 10	09045	QUARTU SANT`ELENA	PRIVATE
1560	200	SARDEGNA	200042	CASA DI CURA S. ANTONIO S.p.A.	VIA CHIRONI 3	09125	CAGLIARI	PRIVATE
1561	200	SARDEGNA	200044	CASA DI CURA VILLA ELENA s.r.l.	VIA DANTE 133	09128	CAGLIARI	PRIVATE
1562	200	SARDEGNA	200045	CASA DI CURA NUOVA	PIAZZA VIRGILIO LOI N.1	09033	DECIMOMANNU	PRIVATE
1563	200	SARDEGNA	200046	CASA DI CURA SAN SALVATORE s.r.l.	VIA SCANO 85	09129	CAGLIARI	PRIVATE
1564	200	SARDEGNA	200048	CASA DI CURA M. AUSILIATRICE S.p.A.	VIA DON BOSCO 4	09123	CAGLIARI	PRIVATE
1565	200	SARDEGNA	200055	CASA DI CURA SANT'ELENA S.p.A.	VIALE MARCONI 160	09045	QUARTU SANT`ELENA	PRIVATE
1566	200	SARDEGNA	200903	I.N.R.C.A.	VIA DELLE CICALE 11	09134	CAGLIARI	PUBLIC
1567	200	SARDEGNA	200904	AZIENDA OSPEDALIERA G.BROTZU	PIAZZALE A. RICCHI N. 1	09134	CAGLIARI	PUBLIC
1568	200	SARDEGNA	200905	AZIENDA OSPEDALIERO UNIVERSITARIA SASSAR	VIA COPPINO 26	07100	SASSARI	PUBLIC
1569	200	SARDEGNA	200906	A.O.U. Cagliari	via ospedale	09124	CAGLIARI	PUBLIC
1570	200	SARDEGNA	200906	A.O.U. Cagliari	via ospedale	09124	CAGLIARI	PUBLIC
