# Supplementary material for: Italian hospitals on the web: a cross-sectional analysis of official websites
Source: BMC Med Inform Decis Mak. 2010 Apr 1;10:17. doi: 10.1186/1472-6947-10-17 (PMC2868065; doi:10.1186/1472-6947-10-17)
Supplement: Additional file 2 — Codebook. List of the eighty-nine items included in the study. [file 1472-6947-10-17-S2.PDF]

Appendix 2: Codebook

a) Technical items

1. Site name appears on browser title bar
2. Active part of the site appears on the browser title bar
3. Name of the hospital at the head of the website
4. Hospital logo at the head of the website
5. Any animation or visual displays can be bypassed
6. Access to the website in foreign languages
7. Website map available
8. Website searcher available
9. Date of last website update
10. Website has HON (Health On the Net foundation) code certification
11. Website has certification of accessibility to people with disabilities (at least W3C WAI-A logo)
12. Website has certification of Cascading Style Sheets validation (W3C CSS logo)
13. Website has certification of the Markup Validation Service (W3C HTML logo)
14. Website has certification of accessibility to people with disabilities provided by the Italian authority on informatics in the public administration (CNIPA, Centro Nazionale per l'Informatica nella Pubblica Amministrazione)
15. Links with other useful websites provided (hospitals, scientific associations, institutions)
16. General disclaimers provided
17. Copyright notice
18. Treatment of surfer personal data statement
19. Website pages can be printed

b) Hospital information and facilities

20. Hospital history
21. Contact details on the homepage or available at a click: hospital postal address
22. Contact details on the homepage or available at a click: telephone and/or fax number
23. Contact details on the homepage or available at a click: e-mail address
24. Contact details on the homepage or available at a click: VAT number
25. Statement of purpose

26. ISO certification on the home-page
27. Organisation chart
28. Information regarding patient privacy
29. Ways of reaching the hospital: car, public transport
30. Map of the hospital
31. Virtual visit to the hospital
32. Public relations office: work hours
33. Public relations office: location
34. Public relations office: telephone and/or fax number
35. Public relations office: e-mail address
36. Services charter
37. Patient’s rights and obligations
38. The results of surveys regarding patient satisfaction are provided
39. Information for General Practitioners is provided
40. Information for foreigners is provided
41. Complementary services: press, cafeteria, television, telephone

c) Admissions and medical services

42. Admission guide: different types of admissions are disclosed
43. Admission guide: information and rules to be followed on admission
44. Admission guide: information and rules to be followed during the hospital stay
45. Admission guide: information and rules to be followed on discharge
46. Admission guide: information and rules to be followed regarding visits by relatives
47. Admission guide: information and procedure for obtaining a copy of the medical documentation
48. Details of how to pay prescription charges or fees
49. Departments or units providing user services: complete list
50. Departments or units providing user services: location
51. Departments or units providing user services: telephone and/or fax number and/or e-mail address
52. Detailed list of outpatient hospital services available (consultation, diagnostic services)
53. Number of hospital beds disclosed
54. Waiting list disclosed
55. Date of last monitoring of the waiting list disclosed

56. Hospital report of the number of admissions in the previous year
57. Doctors’ curricula disclosed
58. Hospital quality indicator: nosocomial infection rate disclosed
59. Hospital quality indicator: inpatient mortality rate disclosed
60. Hospital quality indicator: surgical mortality rate disclosed
61. Hospital quality indicator: others
62. List of employed doctors in alphabetical order
63. List of employed doctors by specialisation
64. Information about private consultations/services and fees
65. List of consultations/services with fees available
66. Cost of consultation/services with fees available

d) Interactive on-line services

67. Appointments for consultation via the Internet
68. Appointments for services/admission via the Internet
69. Other facilities available via the Internet (e.g. documentation)
70. Appointments for consultation/services/admission via the Internet: link on the homepage
71. Possibility to communicate with the hospital via the Internet or e-mail
72. Possibility to ask a specialist a health-related question via the Internet or e-mail
73. Information request form via the Internet or e-mail
74. Suggestions/complaints form via the Internet or e-mail
75. Possibility to sign up for a newsletter
76. A health-related forum is present

e) External activities

77. Possibility to read online or to download health-care booklets
78. Medical glossary available
79. Scientific studies that the hospital promotes or is involved in
80. Undergraduate or postgraduate courses that are held at the hospital
81. Presence of a library
82. Schedule of activities that take place at the hospital: courses, congresses and conferences
83. Publication of the hospital itself
84. Details of job opportunities at the hospital
85. Associations that work at the hospital: voluntary associations

86. Associations that work at the hospital: patient associations
87. Associations that work at the hospital: associations for the defence of patients’ rights
88. Information on how to make a donation to the hospital
89. The hospital in the media: press review
